# Transcriptomic Characterization of Tambaqui (*Colossoma macropomum*, Cuvier, 1818) Exposed to Three Climate Change Scenarios

**DOI:** 10.1371/journal.pone.0152366

**Published:** 2016-03-28

**Authors:** Marcos Prado-Lima, Adalberto Luis Val

**Affiliations:** 1 Brazilian National Institute for Research of the Amazon, Laboratory of Ecophysiology and Molecular Evolution, Manaus, Amazonas, Brazil; 2 Federal University of Western Pará, Institute of Water Science and Technology, Santarém, Pará, Brazil; Hospital Universitario LA FE, SPAIN

## Abstract

Climate change substantially affects biodiversity around the world, especially in the Amazon region, which is home to a significant portion of the world’s biodiversity. Freshwater fishes are susceptible to increases in water temperature and variations in the concentrations of dissolved gases, especially oxygen and carbon dioxide. It is important to understand the mechanisms underlying the physiological and biochemical abilities of fishes to survive such environmental changes. In the present study, we applied RNA-Seq and de novo transcriptome sequencing to evaluate transcriptome alterations in tambaqui when exposed to five or fifteen days of the B1, A1B and A2 climate scenarios foreseen by the IPCC. The generated ESTs were assembled into 54,206 contigs. Gene ontology analysis and the STRING tool were then used to identify candidate protein domains, genes and gene families potentially responsible for the adaptation of tambaqui to climate changes. After sequencing eight RNA-Seq libraries, 32,512 genes were identified and mapped using the *Danio rerio* genome as a reference. In total, 236 and 209 genes were differentially expressed at five and fifteen days, respectively, including chaperones, energetic metabolism-related genes, translation initiation factors and ribosomal genes. Gene ontology enrichment analysis revealed that mitochondrion, protein binding, protein metabolic process, metabolic processes, gene expression, structural constituent of ribosome and translation were the most represented terms. In addition, 1,202 simple sequence repeats were detected, 88 of which qualified for primer design. These results show that cellular response to climate change in tambaqui is complex, involving many genes, and it may be controlled by different cues and transcription/translation regulation mechanisms. The data generated from this study provide a valuable resource for further studies on the molecular mechanisms involved in the adaptation of tambaqui and other closely related teleost species to climate change.

## Introduction

Climate change, resulting mainly from increases in the concentration of greenhouse gases (GHG) in the atmosphere, will affect all human activities and different ecosystems [1–3]. Various climate change scenarios have been proposed based on the intensity of human activities causing environmental degradation. These scenarios provide plausible predictions in several key areas, such as the emissions of GHG and aerosols and environmental and socioeconomic conditions [2,3].

When applied to climate change research, the various climate scenarios help provide a preview of how Earth’s systems will respond to different levels of greenhouse emissions as well as aid the design of strategies to reduce the resulting impacts on organisms [4]. According to the Fourth Assessment Report of the Intergovernmental Panel on Climate Change (IPCC), three main scenarios of climate are foreseen for the year 2100: B1 (soft), A1B (intermediate) and A2 (extreme). These scenarios may vary according to population growth, socioeconomic development and the use of fossil fuels or renewable energy [2].

Several studies have used climate change scenarios foreseen by the IPCC to predict the qualitative and quantitative responses of marine and freshwater ecosystems to environmental changes associated with the accumulation of GHG in the atmosphere [5,6]. These studies suggest that climate change will cause severe disturbances to both marine and freshwater ecosystems, impairing the distribution of species and fishery in various countries [7,8].

The Amazon basin harbors a significant portion of the world’s biodiversity and exhibits the highest diversity of freshwater fishes in the world, with approximately 3,000 fish species [9–11]. The Amazon River and its tributaries are home to species that are still unknown to the scientific community. Despite this, the Amazon region has been subjected to environmental pressures in recent decades, including pressures of anthropogenic origin, such as deforestation, fires and growth of urban centers, and those resulting from global warming [11,12].

Climate change may affect many organisms, ranging from bacteria to mammals [3]. Fishes are especially susceptible to climate change, due mainly to increases in water temperature and variations in the concentrations of dissolved gases, particularly oxygen and carbon dioxide (CO_2_) [6]. Poikilothermic aquatic organisms, such as fishes, can face a new challenge in warm water because it holds less oxygen, resulting in a hypoxic environment. Because each fish species has a specific thermal tolerance, a species may face death and extinction if the water temperature exceeds its thermal tolerance [13–15]. This is an issue of increasing concern because temperatures may continue to increase as a result of global climate change [16].

Tambaqui (*Colossoma macropomum*) is a native fish species of the Amazon basin and member of the family Serrasalmidae [9] that plays an important economic role in the Amazon region [17]. In Brazil, it is the second most commonly raised fish in aquaculture [17]. This species exhibits a high tolerance for environmental changes in dissolved oxygen, temperature and pH [18,19]. Therefore, in the face of global climate change, it is important to understand the molecular mechanisms that confer tolerance in this species.

Molecular methods such as high-throughput RNA sequencing (RNA-Seq) provide the opportunity to investigate the transcriptional response of various organisms, including fishes, to climate change, allowing the identification of mechanisms underlying adaptation to environmental stress [20,21]. Furthermore, RNA-Seq enables the correlation of the number of times that a transcript is sequenced with its abundance in a tissue or organism, which provides evidence of the quantitative gene expression profile [21,22].

To combat the adverse effects elicited by fluctuations in temperature and dissolved gases, fishes have developed the ability to adapt to a wide range of environmental challenges to maintain normal cellular functions [19,23,24]. The biochemical changes induced by stress are attributed to modulation of gene expression, including controlling the cell cycle, DNA and chromatin stabilization, protein folding and repair, the removal of damaged proteins, and energy metabolism [25–27]. While stress response genes have been well characterized in many species [28], there have been relatively few studies of Amazonian organisms, such as tambaqui, particularly in relation to issues of ecological and evolutionary significance, such as climate change [29]. Thus, RNA-Seq provides the possibility to investigate differential expression and identify biological pathways involved in the response of fishes to climate change.

Therefore, the present study aimed to analyze alterations of gene expression in tambaqui when exposed to the B1, A1B and A2 future climate scenarios foreseen by the IPCC for the year 2100.

## Materials and Methods

### Ethics statement

All experimental procedures were carried out in accordance with Brazilian legislation and approved by the Ethics Committee on Animal Use of the Brazilian National Institute for Research in the Amazon (CEUA-INPA) (Protocol 056/2012).

### Climate change exposure

Tambaqui juveniles (15.5±1.9 g and 8.3±0.3 cm) were obtained from a local fish farm (Fazenda Tajá, Amazonas, Brazil) and transported to the Laboratory of Ecophysiology and Molecular Evolution of the Brazilian Institute for Amazonian Research (INPA), Manaus, Amazonas state, Brazil. The fish were maintained in 500 L fiberglass outdoor tanks for one month, for local acclimatization.

Three future climate scenarios provided by the Fourth Assessment Report of the IPCC for the year 2100 [2] were simulated: B1 (soft scenario): 1.5°C and 200 ppm CO_2_ above current levels; A1B (intermediate scenario): 2.5°C and 400 ppm CO_2_ above current levels; and A2 (extreme scenario): 4.5°C and 850 ppm CO_2_ above current levels. The control scenario was the current real-time temperatures and CO_2_ levels (Table 1) in a near-forested area. Sensors measure these parameters every other minute and transmit the data to laboratory computers that control environmental rooms according to the above scenarios.

**Table 1:** Physicochemical parameters of water and air in the control climate environment where the specimens of tambaqui were kept for five and fifteen days while being exposed to the B1, A1B and A2 climate scenarios. The data are reported as the mean ± standard error of the mean.

		pH	Water O_2_ (mg.L^-1^)	Water CO_2_ (ppm)	Water temperature (°C)	Environment CO_2_ (ppm)	Environment temperature (°C)
5 days	Current	6.2±0.3	7.4±0.1	8.8±0.9	24.7±0.2	416±11.7	30.2±0.7
	B1	5.4±0.2	7.3±0.1	11±0.8	25.7±0.2	622.1±17.5	32±0.8
	A1B	5.3±0.2	7.3±0.2	12.3±0.7	26±0.2	830.6±23.4	33±0.8
	A2	5.0±0.1	7.4±0.1	13.8±0.8	27.3±0.3	1257.1±35.4	34.3±0.9
15 days	Current	6.1±0.2	7.4±0.1	8.9±0.8	24.9±0.3	441.1±10.4	30.1±0.7
	B1	5.5±0.2	7.4±0.1	11.9±0.9	25.8±0.2	641.3±15.1	31.7±0.8
	A1B	5.3±0.1	7.5±0.1	15.3±0.6	26.1±0.1	856.6±20.2	32.8±0.8
	A2	5.1±0.1	7.5±0.1	19±0.9	27.6±0.3	1280.9±30.2	34.2±0.9

Tambaqui juveniles were transferred to the conditions of the above climate scenarios two days prior to beginning the experiments. Six 60-L PVC tanks containing four fish per tank were maintained in each climate room. To avoid ammonia accumulation, 30% of the water was replaced every other day using environmentally stabilized water. The pH, O_2_ and CO_2_ levels and temperature of the water were measured daily (Table 1). The fish were fed *ad libitum* once a day using commercial pelleted feed (Nutripeixe, Purina). After each exposure period, one fish was removed from each tank, with a total of six fish per scenario being collected after five days and another six after fifteen days. The fish were subsequently euthanized by rapidly severing their spinal cord with a scalpel, and white muscle samples were collected and immediately stored in liquid nitrogen until RNA isolation.

### RNA purification

Total RNA was isolated from the white muscle specimens from 48 tambaqui using TRIzol (Invitrogen, Carlsbad, CA, USA) according to the manufacturer’s instructions. Total RNA samples were then digested with DNase I to remove potentially remaining genomic DNA, and ribosomal RNA was depleted using the RiboMinus^TM^ Eukaryote Kit for RNA-Seq (Invitrogen, Carlsbad, CA, USA). RNA yields and quality were checked using both an Agilent 2100 Bioanalyzer (Agilent Technologies, Waldbronn, Germany) and a Qubit 2.0 Fluorometer (Invitrogen, Carlsbad, CA, USA). None of the samples showed signs of degradation or impurities (260/280 and 260/230 >1.8, RIN >8.5). Using the total RNA (gDNA-free) from each sample, mRNA was isolated with the Poly(A)Purist^TM^ Kit (Ambion, Austin, TX, USA) according to the manufacturer's protocol.

For each group, equal amounts of mRNA from six fish were pooled for RNA-Seq library construction, with a total of eight RNA-Seq libraries being generated. Using this method, we aimed to increase transcript diversity and to detect rare transcripts in both the control and experimental libraries

### RNA-Seq library construction and sequencing

Approximately 500 ng of mRNA from each pool was used to construct eight RNA-Seq libraries [four for animals after five days and four for animals after fifteen days of exposure to the climate scenarios (Current, B1, A1B and A2)] with the SOLiD™ Total RNA-Seq Kit for Whole Transcriptome Libraries (ABI, Foster, CA, USA), following the manufacturer’s instructions. To improve the statistical analyses, one replicate of each of the eight libraries was constructed, resulting in a total of 16 libraries. Barcodes were used to identify each library individually. The libraries were deposited on two full slides and sequenced using a SOLiD (v.4) sequencer (ABI, Foster, CA, USA) to generate fifty-base sequences.

### Processing of sequence data

The raw reads of 50 bp generated by the SOLiD sequencer from each sample were trimmed and analyzed in CLC Genomics Workbench (CLC Bio, v. 7.5.1). First, adapter sequences and low-quality reads were removed based on default parameters. High-quality reads were then mapped to the *D*. *rerio* genome (the closest species with an available genome). The *D*. *rerio* gene annotations were then used for expression analyses. The expression profiles for each library were analyzed using the EdgeR method implemented in CLC Genomics Workbench, with default parameters. Statistical tests were conducted to identify the differentially expressed genes between the control and experimental samples, subjected to five or fifteen days of exposure to the B1, A1B and A2 climate scenarios. Genes with false discovery rate (FDR)-corrected *P* values ≤0.05 and absolute fold change values ≥2.0 were included in the analysis as differentially expressed genes. Contigs with gene annotations were used for further analysis.

### Gene ontology

All of the differentially expressed genes identified only under the A2 scenario after both five and fifteen days of exposure were split into two groups: up-regulated and down-regulated genes. Then, each group was mapped separately to gene ontology terms using the AmiGO (v. 2.0) program (http://www.geneontology.org/), against the database of annotated genes for *D*. *rerio*. The Gene Ontology (GO) program employs three general categories: molecular function (MF), biological process (BP), and cellular component (CC). For each category, there is a structure of terms and/or more specific levels for categorizing genes.

### Protein interactions

To generate the protein functional interaction network, all of the differentially expressed genes were submitted together, using their gene symbols, to STRING (Search Tool for the Retrieval of Interacting Genes) software (v.10) and subjected to searches against the *D*. *rerio* database to look for known interactions among the genes. The STRING database (http://string-db.org/) is a collection of known and predicted protein associations obtained from the mining of databases and the literature, high-throughput experimental data, and predictions based on genomic analyses of more than 2000 organisms [30]. From the STRING interaction data, we extracted conserved genomic neighborhoods, gene fusion events, phylogenetic profiles or gene co-occurrences across multiple genomes, text mining, experiments, and other databases with the highest confidence scores (0.9) allowed by the STRING schemes.

### Identification of SSR motifs

To identify all repetitive elements in the assembled contigs from tambaqui, all sequences were searched for SSR motifs using the Msatcommander software (v. 0.8.2, with default settings). To be considered a dinucleotide SSR, the sequence must have a minimum of six repeats. All other types of SSRs should have a minimum of five repetitions. To be considered a compound SSR, the interruption between two neighboring SSRs cannot surpass 100 nucleotides. The Primer3 software [31] was used to design PCR primers for flanking all identified SSR motifs.

## Results

To characterize the transcriptional responses of genes affected by climate change in tambaqui, eight RNA-Seq libraries were built after five and fifteen days of exposure to the B1, A1B and A2 climate scenarios. A total of 776,301,503 reads were sequenced, with an average of 97 million of reads per library (Table 2). Each raw read was sequenced from one end, and the length was 50 bp. After filtering to remove adaptors and low-quality sequences, we obtained a total of 116,176,912 trimmed reads. After clustering and *de novo* assembly, 54,206 contigs of high quality, showing lengths ranging from 74 to 1,094 bp were generated. The redundant and ribosomal protein sequences were excluded, and 32,512 genes were identified based on comparison with the *D*. *rerio* database. The raw reads generated in this study have been deposited in the National Center for Biotechnology Sequence Read Archive (NCBI-SRA) database (SRP062336).

**Table 2:** Overview of the RNA-Seq reads acquired from tambaqui after five and fifteen days of exposure to the B1, A1B and A2 climate scenarios.

	5 days				15 days				Total
	Current	B1	A1B	A2	Current	B1	A1B	A2	
Total Reads	83.673.651	116.529.116	96.692.812	79.983.888	93.479.183	98.635.208	112.023.018	95.284.627	776.301.503
Trimmed reads	16.996.336	16.594.828	16.531.446	17.909.708	19.706.164	16.328.332	13.701.912	15.404.522	133.173.248
All uniquely mapped reads to *Danio rerio* genes	4.280.731	4.543.412	4.082.882	4.344.590	4.785.282	3.946.647	3.659.181	3.931.801	33.574.526
Uniquely mapped reads to *Danio rerio* exons	1.031.276	752.684	1.035.552	1.179.861	1.301.147	1.090.509	723.738	953.959	8.068.726

**Fig 1:**
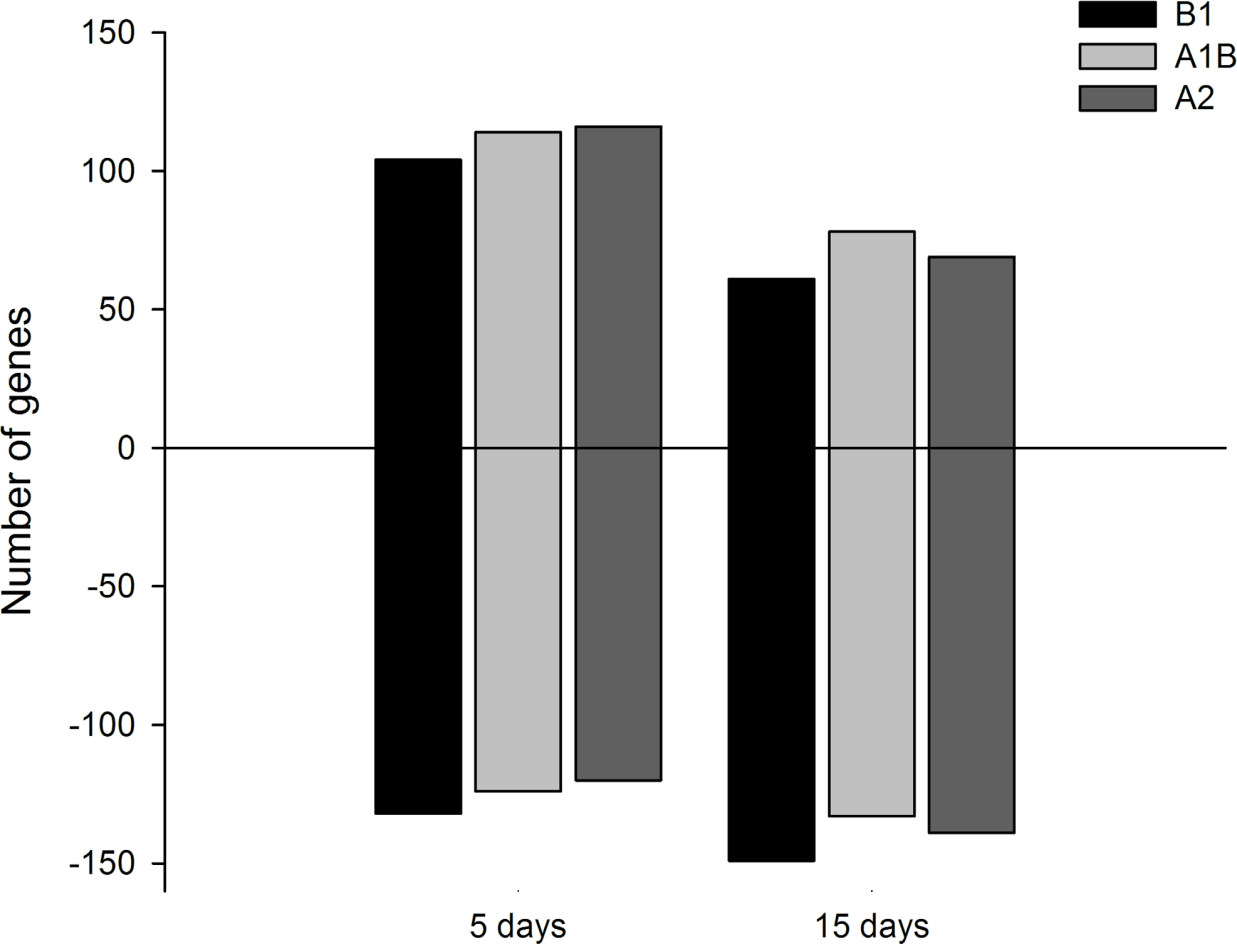
Comparison of the number and direction of differentially expressed transcripts identified in tambaqui after five and fifteen days of exposure to different climate scenarios. The bar chart shows the number of differentially expressed transcripts identified after five days (left bars) and fifteen days (right bars) under the B1, A1B and A2 climate scenarios. Positive and negative values on the Y-axis represent the number of up-regulated and down-regulated transcripts, respectively.

Considering only those genes with a p-value ≤0.05 and a ≥2-fold change in expression across the control and treated libraries, we identified a total of 236 and 209 differentially expressed genes after five and fifteen days, respectively, of exposure to the B1, A1B and A2 climate scenarios (Fig 1). After five days, 104, 114 and 116 up-regulated genes were observed in animals exposed to the B1, A1B and A2 climate scenarios, respectively. The corresponding numbers of down-regulated genes were 132, 124 and 120, respectively. After fifteen days, 61, 78 and 69 up-regulated genes were identified in animals exposed to B1, A1B and A2, respectively. The corresponding numbers of down-regulated genes were 149, 133 and 138, respectively. A complete list of the differentially expressed genes, including the level of expression, can be found in the Supplementary material (S1 and S2 Tables).

The list of differentially expressed genes showed several genes that play roles in energy production, protein folding and maintaining cellular homeostasis, among other functions. Genes encoding aldolases (*aldoaa*, *aldoab* and *aldocb*), enolases (*eno1*, *eno3*), lactate dehydrogenase (*ldha*), pyruvate kinase (*pkmb*), cytochrome c oxidase (*cox5bs*, *cox4i2*, *cox8b*) and chaperones such as *Hsp40* (*Dnaja2*, *Dnajc7*) and Hsp90 (*hsp90aa1*.*1* and *hsp90aa1*.*2*) and genes responsible for protein folding (such as *hmgb1a* and *pfdn2*) were identified. The main genes responsible for the adaptation of tambaqui to climate change were grouped according to the current literature, metabolic routes and the GO database (Table 3).

**Table 3:** Tambaqui genes^1^ affected by exposure to climate change scenarios.

Gene symbol	Gene name	Gene Ontology^2^
dnaja2^3^	DnaJ (Hsp40) homolog, subfamily A, member 2	response to temperature stimulus (BP) and heat shock protein binding (MF)
dnajc7^3^	DnaJ (Hsp40) homolog, subfamily C, member 7	response to temperature stimulus (BP) and heat shock protein binding (MF)
hmgb1a^5^	high mobility group box 1a	response to temperature stimulus (BP) and chromatin remodeling (BP)
hsp90aa1.1^3^	heat shock protein 90, alpha (cytosolic), class A member 1, tandem duplicate 1	chaperone-mediated protein (BP) and complex assembly (BP)
hsp90aa1.2^4^	heat shock protein 90, alpha (cytosolic), class A member 1, tandem duplicate 2	chaperone-mediated protein (BP) and complex assembly (BP)
pfdn2^5^	prefoldin subunit 2	protein folding (BP) and unfolded protein binding (MF)

^1^Genes selected based on the stress response using Blast X and the scientific literature.

^2^Functional annotation associated with the *Danio rerio* database. Gene ontology (GO) categories: biological process (BP) and molecular function (MF).

^3^Gene showing differential expression only after five days of exposure to the B1, A1B and A2 climate scenarios.

^4^Gene showing differential expression only after fifteen days of exposure to the B1, A1B and A2 climate scenarios.

^5^Gene showing differential expression after both five and fifteen days of exposure to the B1, A1B and A2 climate scenarios.

**Fig 2:**
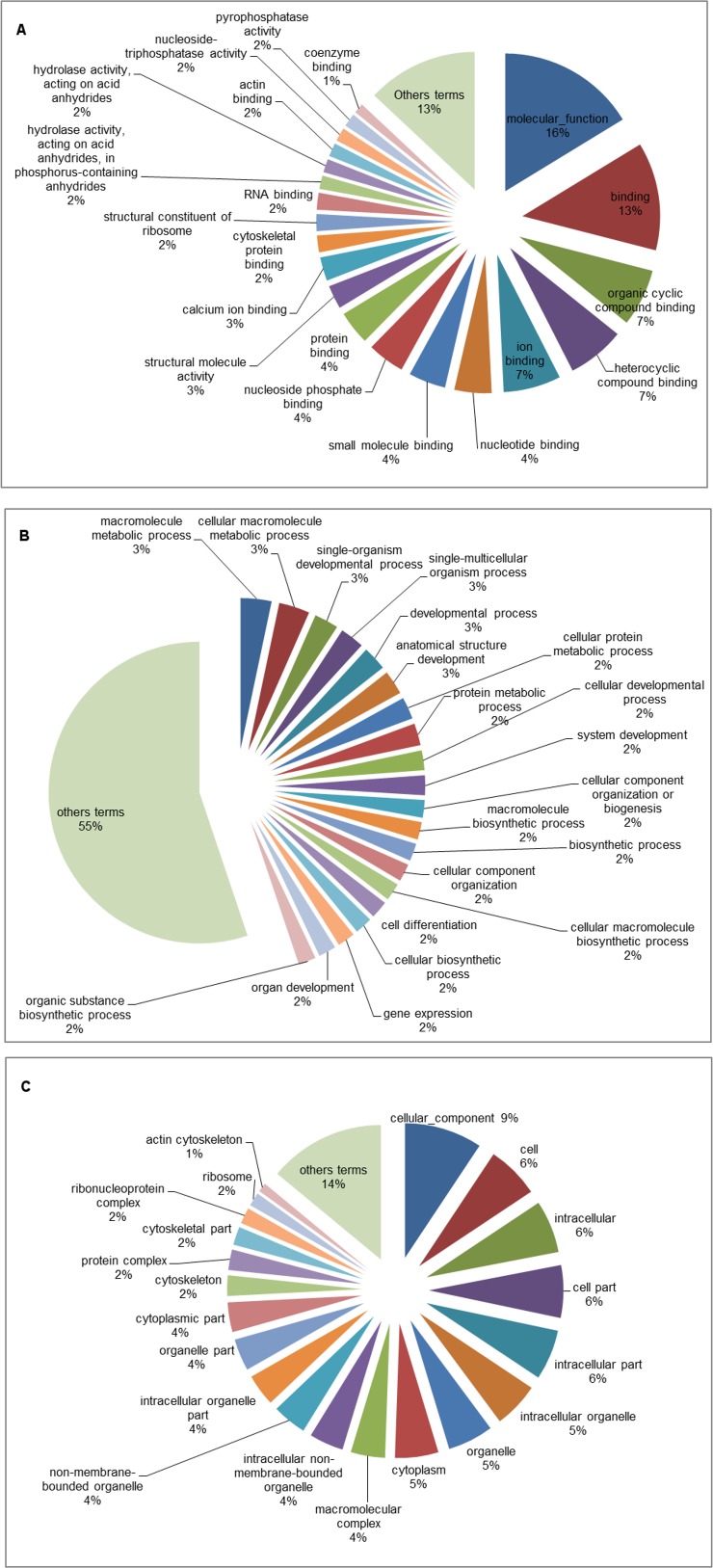
Pie chart of the top 20 GO terms associated with up-regulated genes identified in tambaqui after five days of exposure to the B1, A1B and A2 climate scenarios. Representation of the top 20 GO terms from the molecular function (A), biological process (B) and cellular component (C) categories in the experimental groups compared with the control. The terms other than the top 20 are represented together.

**Fig 3:**
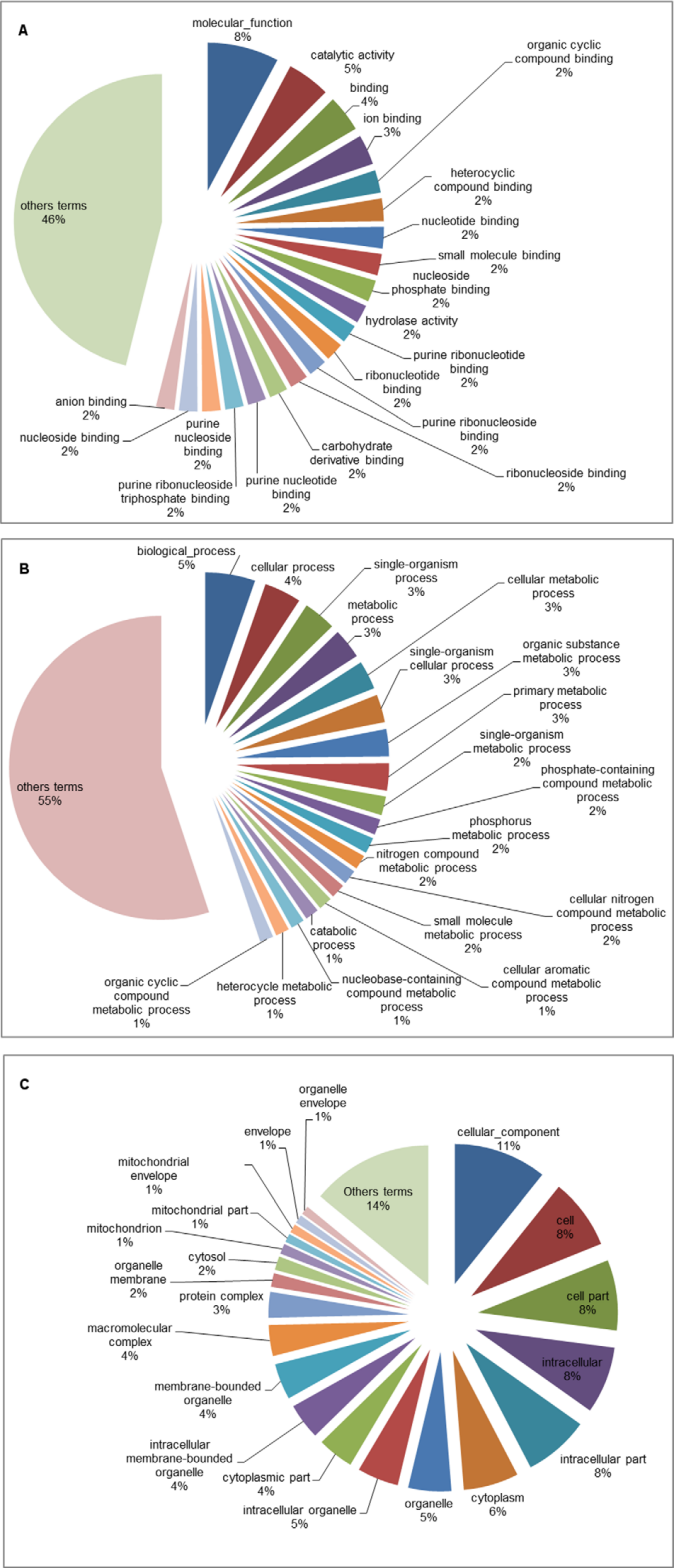
Pie chart of the top 20 GO terms associated with down-regulated genes identified in tambaqui after five days of exposure to the B1, A1B and A2 climate scenarios. Representation of the top 20 GO terms from the molecular function (A), biological process (B) and cellular component (C) categories in the experimental groups compared with the control. The terms other than the top 20 are represented together.

**Fig 4:**
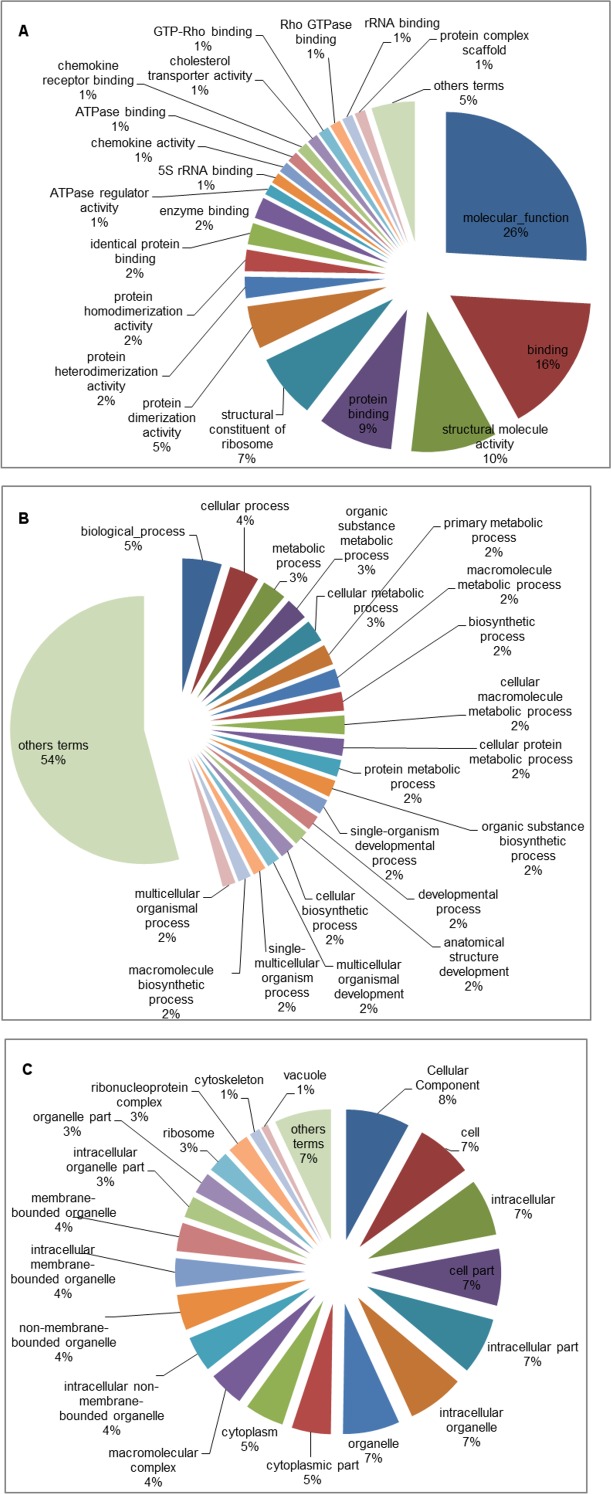
Pie chart of the top 20 GO terms associated with up-regulated genes identified in tambaqui after fifteen days of exposure to the B1, A1B and A2 climate scenarios. Representation of the top 20 GO terms from the molecular function (A), biological process (B) and cellular component (C) categories in the experimental groups compared with the control. The terms other than the top 20 are represented together.

**Fig 5:**
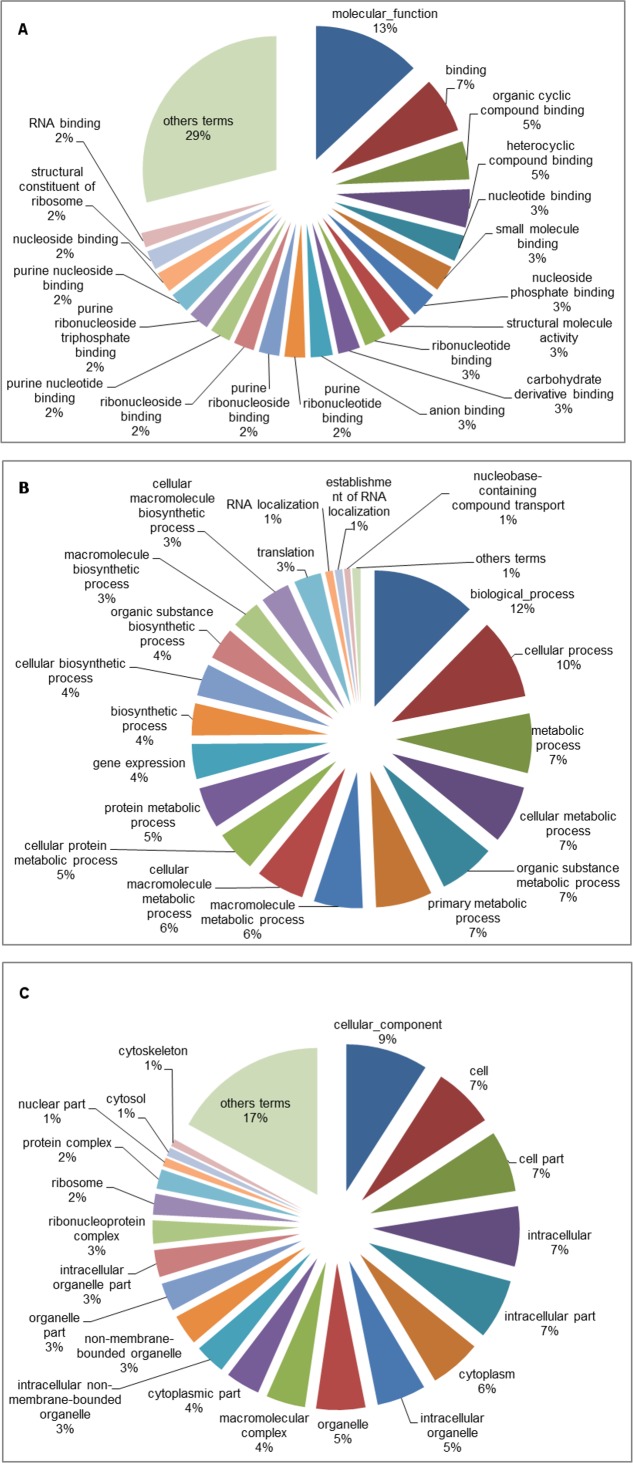
Pie chart of the top 20 GO terms associated with down-regulated genes identified in tambaqui after fifteen days of exposure to the B1, A1B and A2 climate scenarios. Representation of the top 20 GO terms from the molecular function (A), biological process (B) and cellular component (C) categories in the experimental groups compared with the control. The terms other than the top 20 are represented together.

The GO database was used to categorize the differentially expressed genes identified only under the A2 scenario after five and fifteen days of exposure. The A2 scenario was chosen because it was the most extreme scenario considered in the present study. Among all the genes identified, 241 were successfully categorized, 82 of which were up-regulated, while 159 were down-regulated. Analysis of the GO term distribution of the up-regulated genes at five days revealed 235 terms (MF: 48, BP: 138 and CC: 49) (Fig 2), while there were 263 terms associated with down-regulated genes (MF: 118, BP: 91 and CC: 54) (Fig 3). The GO analysis using the fifteen-day library revealed 257 terms for the up-regulated genes (MF: 25, BP: 194 and CC: 38) (Fig 4) and 180 terms for the down-regulated genes (MF: 83, BP: 23 and CC: 74) (Fig 5). For three broad categories (MF, BP and CC), four pie chart graphs were generated: two containing the corresponding top 20 terms linked to up-regulated genes after five and fifteen days and another two containing the top 20 terms linked to down-regulated genes identified after five and fifteen days under the A2 climate scenario. In both cases, the top 20 terms were those with the highest percentage of representation within each of the three general categories. The terms other than the top 20, with low percentages, usually ranging from 1 to 2% of the total representation, were grouped and are also represented in the pie chart.

**Fig 6:**
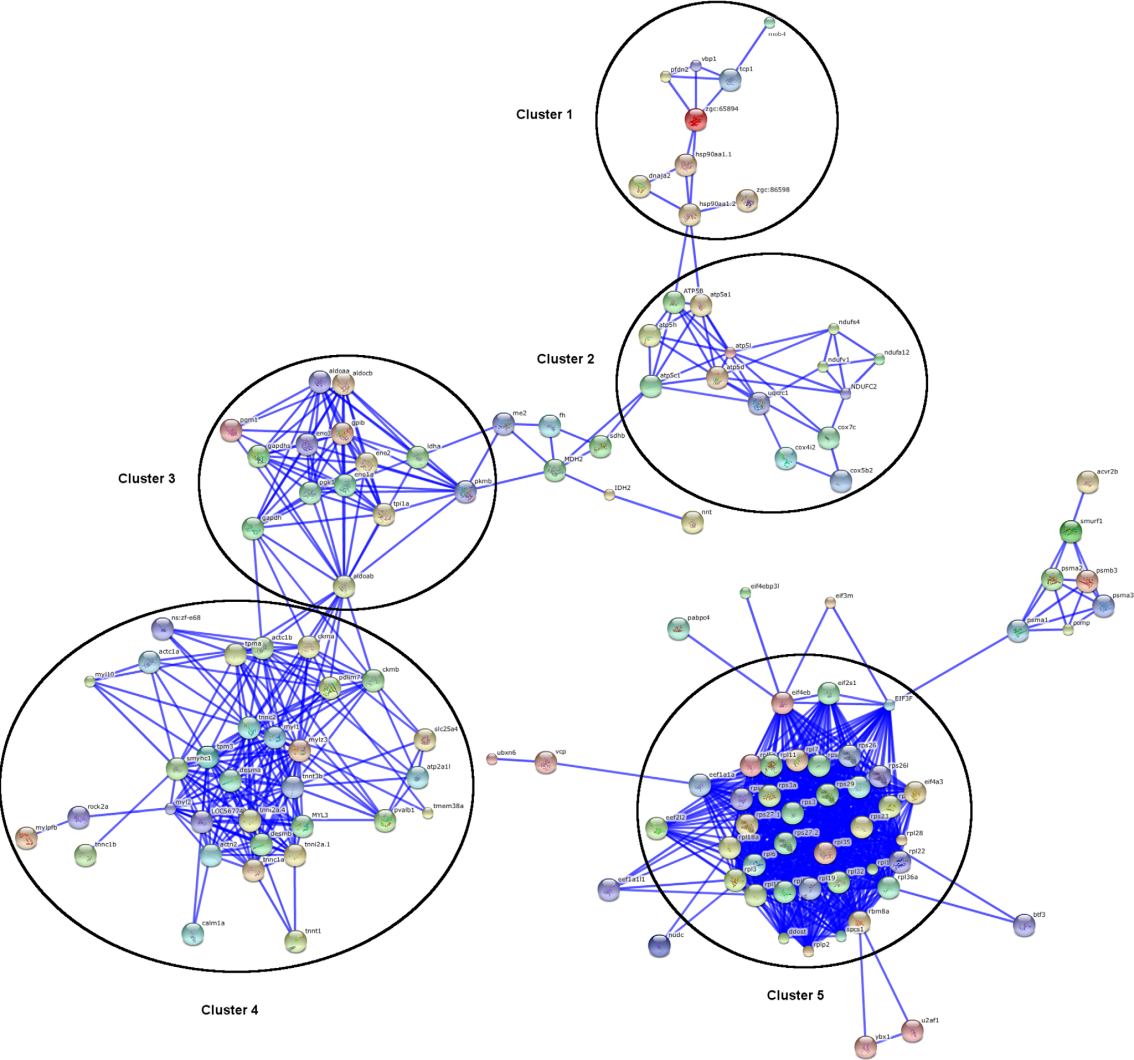
Gene network interactions identified using STRING software (v.10). **Interactions of genes (or proteins) expressed in the white muscle of tambaqui after five and fifteen days of exposure to the B1, A1B and A2 climate scenarios.** Circles with different colors represent different genes (or proteins). Blue lines represent strong interactions between genes (or proteins). Nodes with no interactions have been hidden.

STRING software was used to generate a confidence gene interaction network merging all of the differentially expressed genes identified after five and fifteen days in animals exposed to the B1, A1B and A2 climate scenarios (Fig 6). The proteins are represented with nodes. Direct protein-protein interactions are represented with continuous blue lines. Due to the high number of interactions observed, the settings were changed to only identify gene expression with the “highest confidence” (score >0.9). Nodes with no interactions were hidden. A total of 985 interactions (S3 Table) were obtained using 296 nodes among the analyzed genes. The gene network showed five major clusters. Cluster 1, Cluster 2, Cluster 3 and Cluster 4 exhibited interconnected interactions and sparsely connected sub-networks. Only Cluster 5 showed no connections with the other clusters. The lists of genes grouped in each of the five clusters are highlighted in S1–S5 Figs and S4 Table.

**Fig 7:**
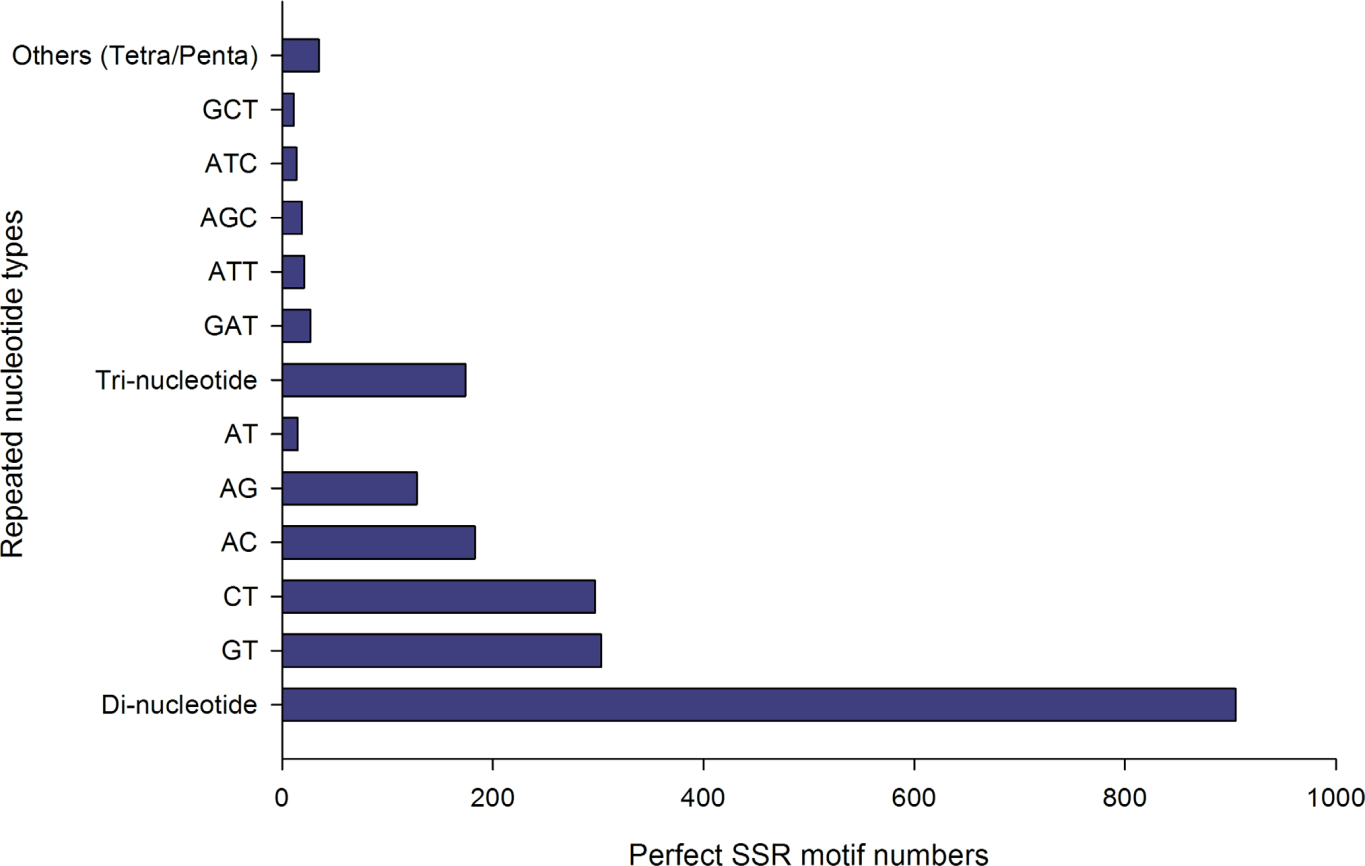
Distribution of simple sequence repeats found in tambaqui sequences.

A total of 1,201 SSRs, comprising 93% simple and 7% compound motifs, were detected among the tambaqui contigs (Fig 7). These SSRs included the following types of repeats: 81% dinucleotides, 16% trinucleotides and 3% tetra/pentanucleotides. Among the dinucleotide repeats, the GT (33.5%) and CT types (32.8%) were most abundant, while GAT was the most frequent trinucleotide repeat (15.5%). Based on the detected SSRs, 88 primer sets were successfully designed and will be tested in natural or captive populations (S5 Table).

## Discussion

Climate change is likely to endanger regions with high biodiversity, such as the Amazon. Although several studies have sought to predict the impacts of climate change in the Amazon, they are generally based on computer models that do not always take into account all of the hidden characteristics of the rainforest. Thus, we used the second most commonly farmed species in Brazil, and the most commonly farmed species in the Amazon, to investigate the main impacts of climate change on the regulation of gene expression in fish exposed to the B1, A1B and A2 climate scenarios foreseen by the IPCC for the year 2100.

In the present study, a transcriptome-level analysis was conducted to determine how tambaqui will respond to exposure to climate change using the NGS and RNA-Seq methods. Two critical factors for fish survival were tested at different levels during five and fifteen days of exposure to the B1, A1B and A2 climate scenarios: temperature and CO_2_ levels. Because thermal stress and CO_2_ elevation have broad biological effects on organisms, the transcriptional responses to these parameters are expected to be highly diverse across a number of genes found in ectothermic species such as tambaqui. This study confirms that numerous genes are differentially expressed in tambaqui under the B1, A1B and A2 climate scenarios, and several pathways are involved (S1 and S2 Tables). However, according to the GO tool, a few key pathways contained a high proportion of differentially expressed transcripts, including the mitochondrion, protein binding, protein metabolic process, metabolic processes, gene expression, structural constituent of ribosome and translation categories.

In the present study, SOLiD sequencing allowed the identification of 32,512 genes. Considering that no sequenced genome currently exists for tambaqui, comparison with the *D*. *rerio* transcriptome was performed. Because *D*. *rerio* has a total of 34,460 annotated genes [32], if we consider that the two species have the same number of genes, the identified genes in the present work would represent approximately 94% of the genes of the tambaqui transcriptome and can therefore be employed in various approaches to evaluate information on the transcriptome, genome and genetic breeding of tambaqui. The tool used in the present study allowed us to identify 388 genes that showed different levels of expression according to the time of exposure and the climate scenario. Evidence of acclimation was indicated by the 11.4% decrease in the number of differentially expressed transcripts from five to fifteen days (Fig 1). Despite the short period of time, this result is in accordance with theories on acclimation to heat stress under which organisms can regulate their heat shock response over time [15], as opposed to the theory in which immediate survival is a priority [33]. However, the current results demonstrate that from five to fifteen days, acclimation occurs throughout much of the transcriptome and is not limited to genes related to heat shock, such as chaperones.

Climate change may introduce a number of environmental challenges for fishes due to increased temperatures above the optimal range, leading to altered transcription of genes involved in protein folding and heat-shock responses [29,34,35], ribosomal and metabolism-related genes [27,36,37] and genes associated with cell cycle arrest and apoptosis [23,29,34]. Ectothermic animals, such as fishes, generally show temperature-dependent oxygen consumption [38]. Considering that increases in temperature may induce low-oxygen stress because oxygen solubility is reduced in warmer water [18], fishes may also experience hypoxia at elevated temperatures due to a reduced binding capacity of hemoglobin for oxygen transport [19,39].

A sufficient oxygen supply is fundamental to ensuring optimal growth, foraging and reproduction performance in fishes [13]. Under these conditions, the tambaqui have developed several adaptation mechanisms to safeguard survival ability. For example, tambaqui are able to expand their lower lip to direct the first film of the water column, which is richer in oxygen, over their gills [18]. Simultaneously, the species can reduce erythrocytic ATP and GTP to increase Hb-O_2_ affinity [18,40]. All of these biochemical and physiological adaptations are important for increasing the thermal tolerance of tambaqui and buffering the negative effects of low oxygen concentrations in water.

In the present study, at least four gene groups were identified among all of the differentially expressed genes. The first group comprised several types of heat shock proteins (Hsps), including *Hsp90* and *Hsp40* (*dnaja2*, *dnajc7*). The expression levels of these genes changed after five and fifteen days under the B1, A1B and A2 climate scenarios. *Hsps* are a well-studied group of highly conserved, ubiquitously distributed genes that are expressed upon exposure to various stress factors, including elevated temperatures [41]. *Hsps* are part of an important mechanism that helps organisms survive under different environmental conditions, such as thermal stress [41]. *Hsp90* is a highly conserved molecular chaperone that has been proposed to act as a hub for the signaling network and protein homeostasis associated with diverse physiological processes, including the heat shock response, signal transduction and stress responses, in eukaryotic cells [42]. Our findings suggest that Hsps are directly involved in the thermal tolerance of tambaqui.

The second group of differentially expressed genes were involved in ATP-derived energy production. Genes related to glycolysis, such as *aldoaa*, *aldoab*, *aldocb*, *eno1a*, *eno3*, *ldha*, and *pkmb*, were down-regulated under all three tested climate scenarios. Similarly, genes such *fh*, *ndufs4*, *cox5b2*, and *mdh2*, which play important functions in the tricarboxylic acid cycle and electron transport chain, were down-regulated in the white muscle of tambaqui under all three tested climate scenarios. These results suggest an impairment of several ATP-generating enzymes, which can cause an increase in the expression of transcription factors and target genes as well changes in the fatty acid composition of membranes to equilibrate the cellular energy balance and to maintain cellular homeostasis [43].

The third group consisted of genes that act as translation initiation factors. These genes were repressed in tambaqui after fifteen days of exposure to all three climate scenarios (*eef1a1a*, *eef1a1l1*, *eef1a2*, *eif2s1b*, *eif4a3*, *eif4eb*, and *eif4ebp3l*). These genes encode proteins that are components of the eukaryotic translation initiation factor complex, which is fundamental for several steps in the initiation of protein synthesis through association with the 40S ribosome, and facilitate the recruitment of *eif-1*, *eif-1a*, *eif-2*, and *eif-5* to form a complex known as the 43S pre-initiation complex (43S PIC), which is necessary for scanning mRNA for the translation-initiation codon AUG [44,45]. Another important translation initiation factor that was identified was the high-mobility group b1 protein, encoded by *hmgb1*. This protein plays key roles in the immune system, transcription initiation and the assembly of numerous nucleoprotein complexes that are critical to cell function and the formation of enhanceosome complexes. Through these functions, *hmgb1a* acts as a compensatory modulator of transcription in response to increased temperature, making it a global temperature sensor that reduces the expression of key genes in response to elevated temperature [46]. Hmgb1a may be also involved in the modulation of the immune system of tambaqui in response to diverse climate changes scenarios. However, additional studies are needed to confirm this hypothesis.

The fourth and last group was composed of genes that are related to the production of constituents of ribosomes or are important in the assembly and stability of ribosomal subunits. Some of these genes were significantly repressed in tambaqui, especially after fifteen days (*rpl3*, *rpl7*, *rpl13*, *rpl18a*, *rpl35*, *rps10*, *rps27*.*1* and *rpsa*), while others (*rpl5a*, *rpl14*, *rpl23*, *rpl32*, *rplp2* and *rps26*) showed varying expression levels. Similar results have been obtained following thermal stress in Arctic char [27]. These results, together with the translation initiation factors that were found to be repressed, indicate that commitment of the translational machinery after fifteen days of exposure preserves the synthesis of specific stress-responsive proteins to enhance cell survival under climate changes, which can cause disequilibrium of vital functions, such as metabolism, reproduction, growth and competitiveness [13,14].

As explained above, the list of differentially expressed genes indicates that the cellular responses to climate change in tambaqui are complex, involving a number of genes, and may be controlled by different cues and transcription/translation regulation mechanisms, as observed in other fishes [29,34,47] and various other types of organisms [48,49], including humans [50].

When exposed to thermal stress, fishes need to increase their oxygen supply in response to an elevated metabolic rate, resulting in behavioral changes [47]. Consequently, food consumption and foraging activities may be increased at higher temperatures, increasing the energy requirement of the fishes. However, if a constant search for food is not successful, fishes will be unable to maintain their growth rates [51]. Furthermore, under conditions of increased CO_2_, the effects caused by thermal stress are potentiated, impairing feeding and growth [52]. Another consequence is that increases in temperature have a significant impact on predator–prey interactions, potentially leading to ecosystem imbalances with consequences at various food chain levels [53,51].

Gene ontology is commonly used to categorize gene products and to standardize their representation across different species or environmental stress situations [54–56]. To characterize transcriptional events over time, only the differentially expressed genes related to the A2 climate scenario were subjected to GO analysis (Figs 2–5). The results showed that the up-regulated genes were related to the following categories: protein binding (MF), protein dimerization activity (MF), protein heterodimerization activity (MF), protein homodimerization activity (MF), and identical protein binding (MF). This is expected under thermal stress given that proteins such as HSPs are involved in protein folding and unfolding, providing thermo-tolerance in cells upon exposure to heat stress [41]. In the opposite situation, the down-regulated genes were related to the following categories: cellular metabolic process (BP), phosphorus metabolic process (BP), organic substance metabolic process (BP), primary metabolic process (BP), protein metabolic process (BP), macromolecule metabolic process (BP) and cellular protein metabolic process (BP). These results, together with those for the gene expression (BP) and translation (BP) categories, indicate suppression of various metabolic processes to ensure cellular homeostasis and, thus, overcome the environmental challenge. The categories mentioned above appeared at low percentages after five days, but their percentages almost doubled after fifteen days of exposure to the A2 climate scenario, indicating that these mechanisms that enable organisms to minimize some temperature-related effects were already present at five days and increased after fifteen days. The results of GO analysis followed a similar pattern to those observed in transcriptomic analyses of other fish species [34,46,57].

Protein functional interaction networks, such as the one we generated using the STRING database, are commonly employed to predict protein–protein associations derived from high-throughput experimental data or from the mining of databases and the literature as well as predictions based on genomic analyses of more than 2,000 organisms [30]. We searched the interactions among all of the differentially expressed genes obtained after five and fifteen days of exposure to the three climate scenarios and used STRING to construct an interaction network (Fig 6). The 296 nodes allowed the identification of five clusters (S1–S5 Figs, S4 Table). A central interplay cluster was not detected, although cluster 1 appeared to start a chain of interactions that extended to cluster 4. Cluster 1 included genes related to the folding and unfolding of proteins, such as *pdfn2*, *dnaja2*, *hsp90aa1*.*1* and *hsp90aa1*.*2*, which are known to control gene expression in a number of organisms under thermal stress [41,35,48]. *Hsp90* (also known as endoplasmin) is a molecular chaperone that functions in the processing and transport of secreted proteins [58]. The up-regulation of *Hsp90* is often used as a hallmark of endoplasmic reticulum stress [29]. *Hsp90* interacts with more than 100 proteins, and some of its notable partners include kinases, nuclear hormone receptors, transcription factors, and ion channels [58]. Clusters 2 and 3 comprised genes related to glycolysis and the mitochondrial respiratory chain, including *pkmb*, *ldha*, *aldoab*, *eno2*, *atp5d*, and *cox5b2*. Cluster 5 mainly consisted of translation factors (*eif2s1*, *eif4eb*, *eif3f*, and *eif1a1a*) and genes related to rRNA processing and ribosomal biogenesis (*rpl11*, *rpl7*, *rps 3*, and *rps29*) that play key roles in the regulation of gene expression. Ribosomal proteins are potential biomarkers of tolerance to thermal stress in fishes [27].

The 1201 SSRs identified and 88 primer sets designed in the current study (S5 Table) provide a valuable resource for the development of molecular tools for tambaqui and other closely related species. Given that only 41 SSR loci had previously been developed for tambaqui [59–61], the 88 primer sets designed in the present study more than double the number of SSR loci currently available. The high number of dinucleotide repeats (Fig 7) observed in tambaqui (approximately 81%) is consistent with previous studies in fishes and other aquatic organisms [62,63]. SSR markers have been efficiently used for the identification of individuals, population diversity and quantitative trait loci (QTL) analyses, and genetic breeding of stocks [64,65]. The SSR loci developed here must be validated before use in future applications.

## Conclusions

This is the first RNA-Seq-based study of the transcriptional response of tambaqui exposed to climate change. Several candidate genes that can be employed to screen for genetic markers for climate change were identified. Using GO enrichment analysis and STRING software, we identified diverse biological processes and intracellular pathways related to the functional impacts of the observed changes in transcript expression. The obtained results provide clues toward elucidating the molecular mechanisms underlying the regulatory networks of gene expression during climate change exposure. In addition, an analysis of repetitive elements was conducted, and SSRs were identified for future marker development and linkage analysis. The large number of differentially expressed genes recorded in this study suggests that a complex combination of genes has evolved in tambaqui as a response to climate change scenarios; in particular, these genes include chaperones, energetic metabolism-related genes, translation initiation factors and ribosomal genes. Overall, the results of this study on the tambaqui transcriptome should serve as a valuable resource for future genetic or genomic studies.

## Supporting Information

S1 Fig Tambaqui genes identified after five and fifteen days of exposure to the B1, A1B and A2 climate scenarios grouped in cluster 1 using STRING software (v. 10). (TIFF)

S2 Fig Tambaqui genes identified after five and fifteen days of exposure to the B1, A1B and A2 climate scenarios grouped in cluster 2 using STRING software (v. 10). (TIFF)

S3 Fig Tambaqui genes identified after five and fifteen days of exposure to the B1, A1B and A2 climate scenarios grouped in cluster 3 using STRING software (v. 10). (TIFF)

S4 Fig Tambaqui genes identified after five and fifteen days of exposure to the B1, A1B and A2 climate scenarios grouped in cluster 4 using STRING software (v. 10). (TIFF)

S5 Fig Tambaqui genes identified after five and fifteen days of exposure to the B1, A1B and A2 climate scenarios grouped in cluster 5 using STRING software (v. 10). (TIFF)

S1 Table The list of differentially expressed genes (Log_2_FC) identified in tambaqui after five days of exposure to the B1, A1B and A2 climate scenarios. Up-regulated genes are shown with positive values, and down-regulated genes are shown with negative values.
(DOCX)

S2 Table The list of differentially expressed genes (Log_2_FC) identified in tambaqui after fifteen days of exposure to the B1, A1B and A2 climate scenarios. Up-regulated genes are shown with positive values, and down-regulated genes are shown with negative values.
(DOCX)

S3 Table Protein interactions identified in tambaqui after five and fifteen days of exposure to the B1, A1B and A2 climate scenarios using STRING software (v. 10). (DOCX)

S4 Table Tambaqui genes identified after five and fifteen days of exposure to the B1, A1B and A2 climate scenarios grouped in five clusters using STRING software (v. 10). (DOCX)

S5 Table Primers and types of SSR motifs found in tambaqui sequences. (XLSX)
