# Supplementary figures and images for: Transcriptomic Characterization of Tambaqui (Colossoma macropomum, Cuvier, 1818) Exposed to Three Climate Change Scenarios
Source: PLoS One. 2016 Mar 28;11(3):e0152366. doi: 10.1371/journal.pone.0152366 (PMC4809510; doi:10.1371/journal.pone.0152366)

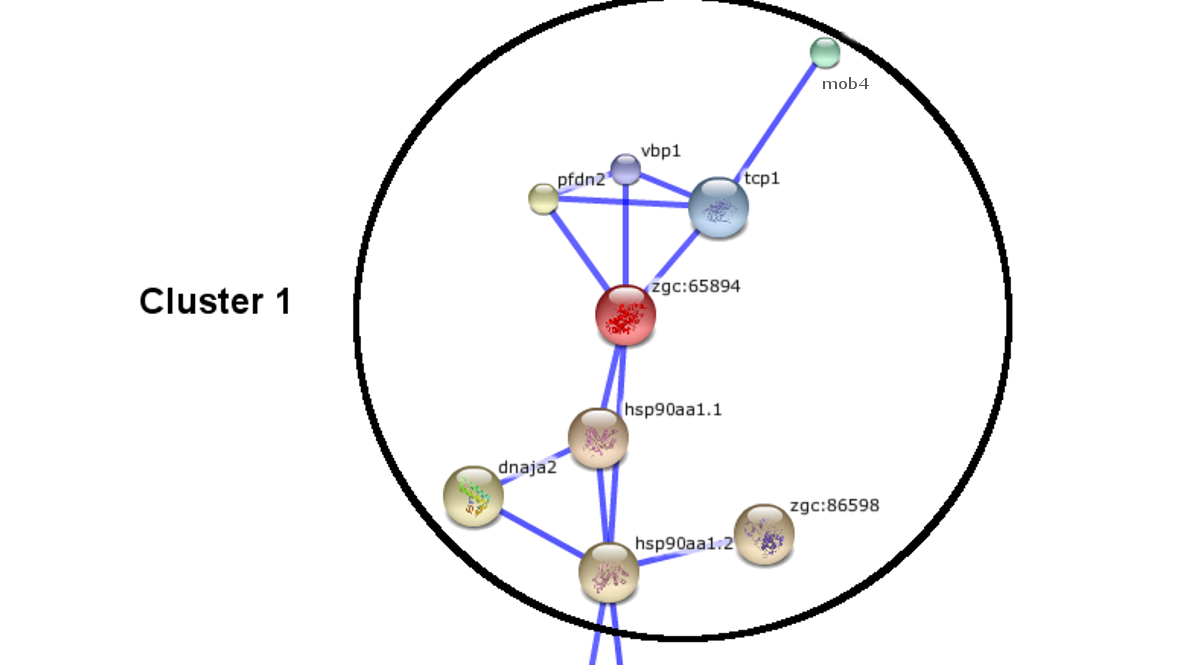

Supplement: S1 Fig — (TIFF) [file pone.0152366.s001.TIFF]

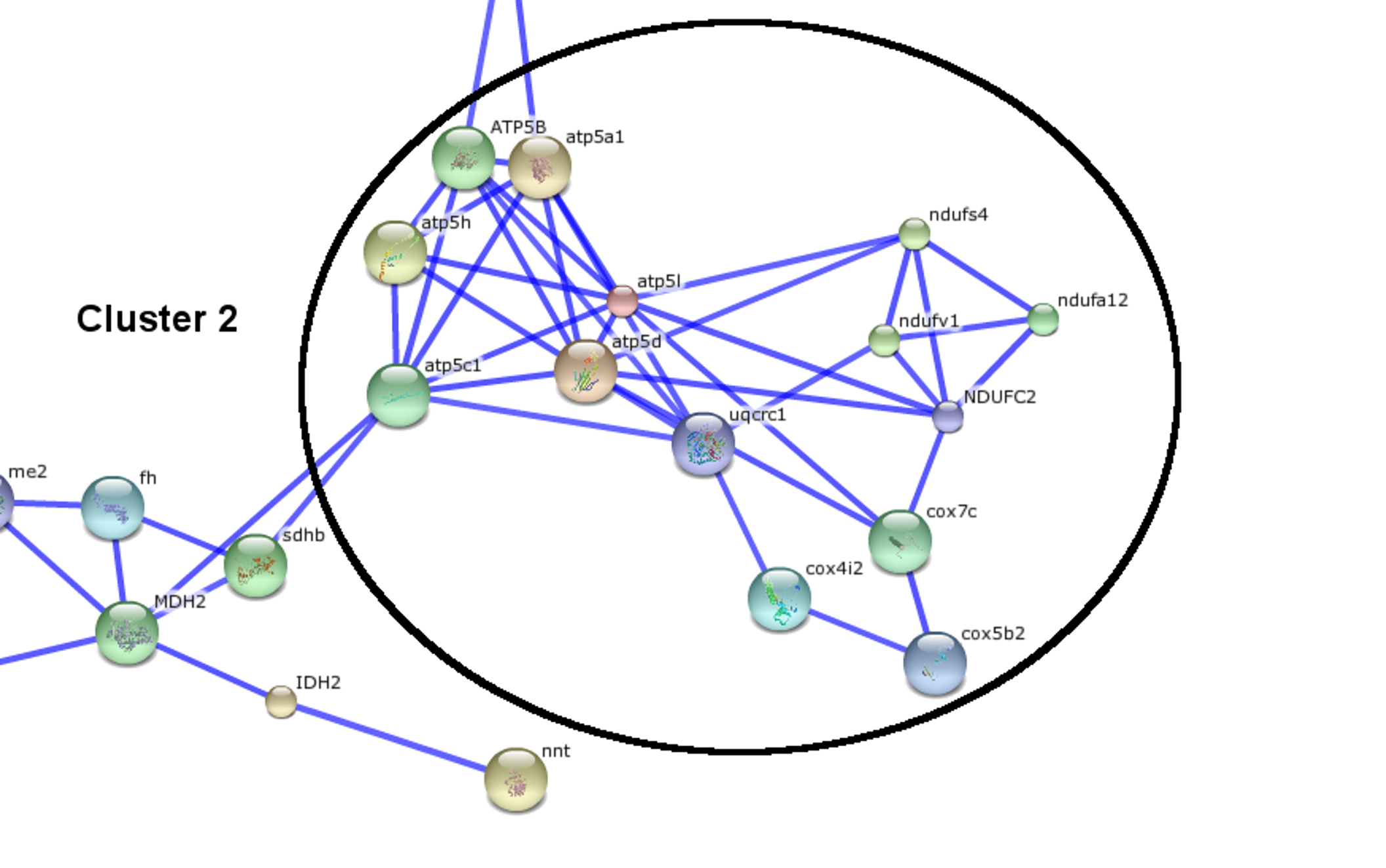

Supplement: S2 Fig — (TIFF) [file pone.0152366.s002.TIFF]

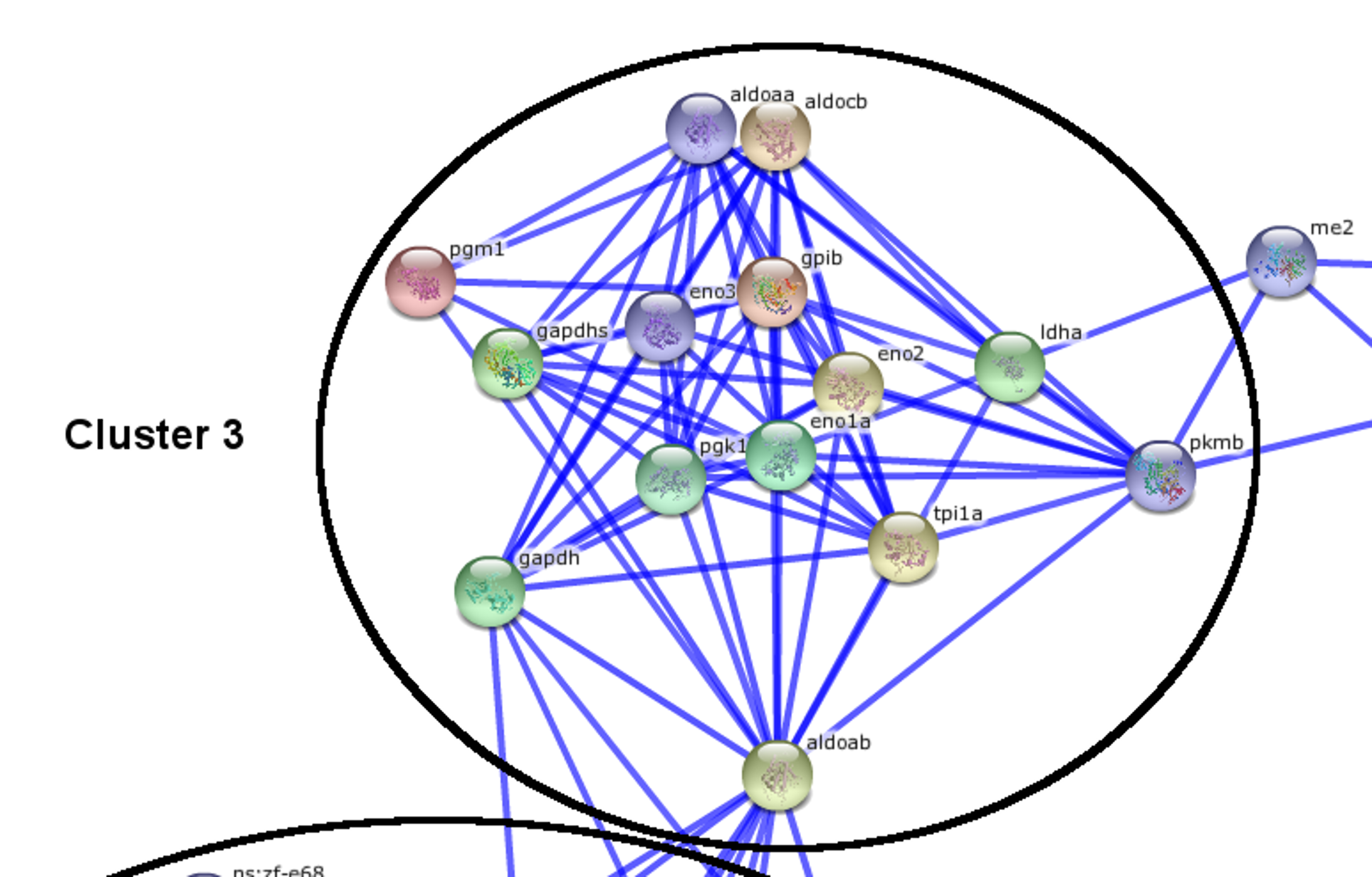

Supplement: S3 Fig — (TIFF) [file pone.0152366.s003.TIFF]

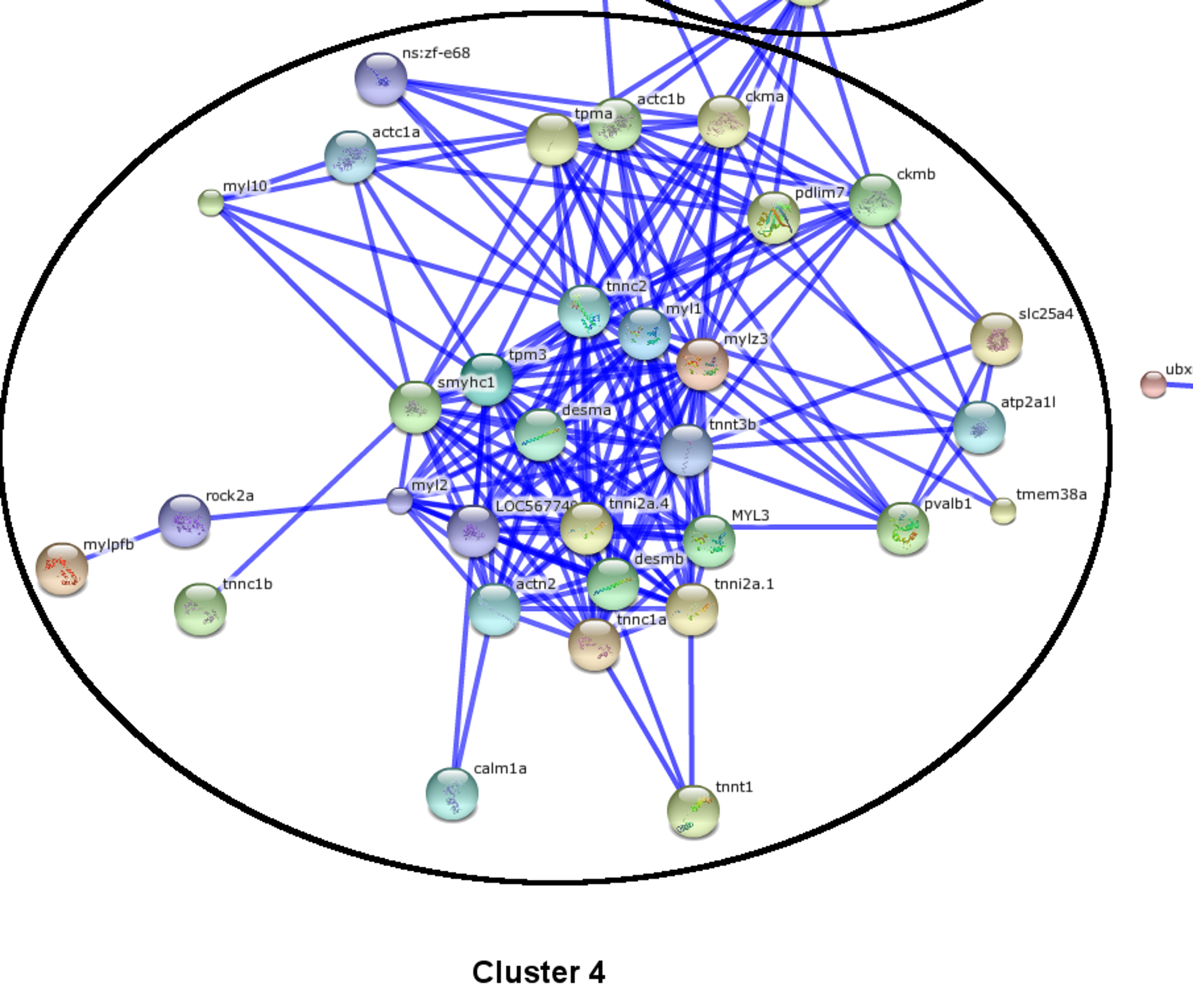

Supplement: S4 Fig — (TIFF) [file pone.0152366.s004.TIFF]

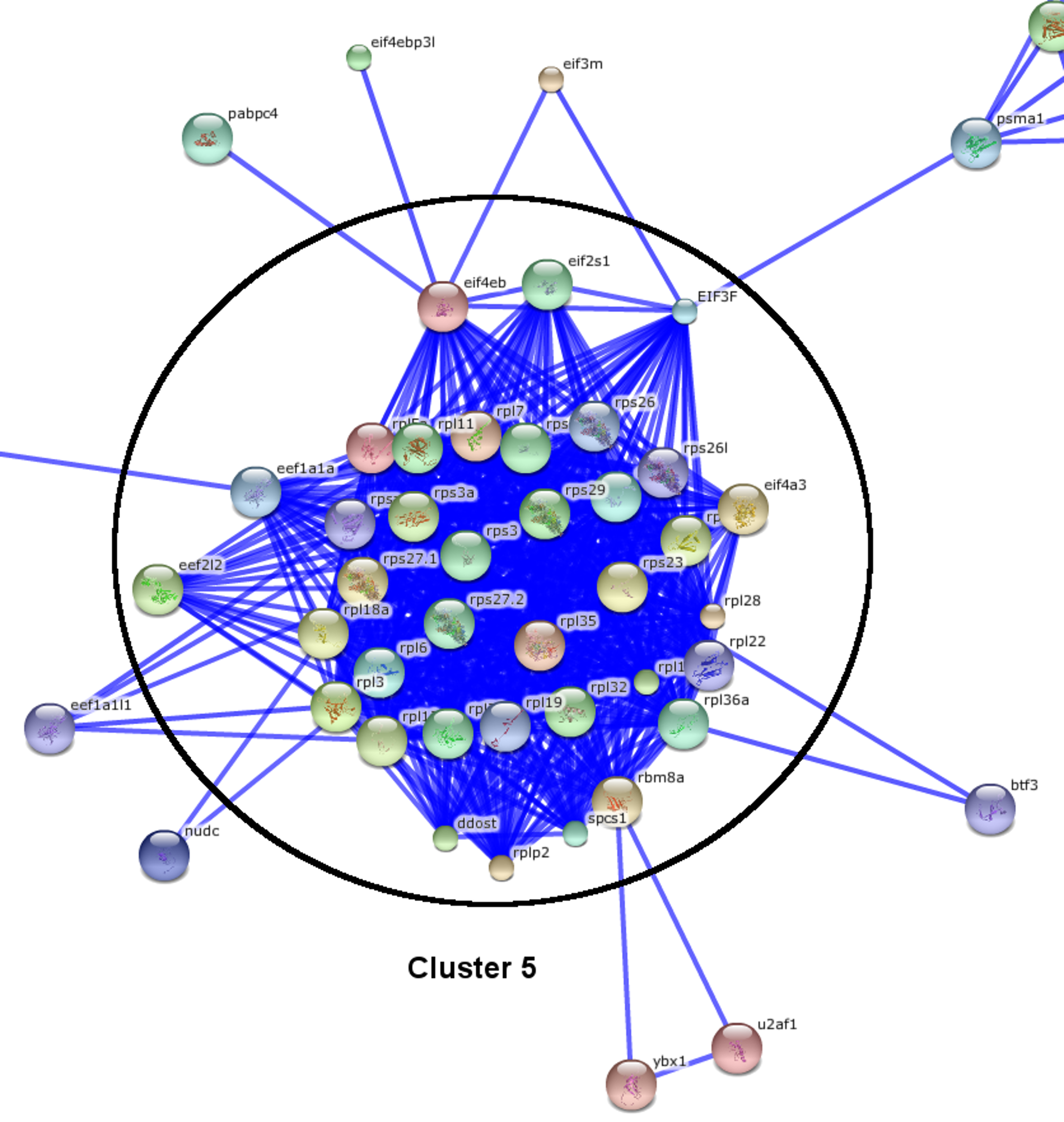

Supplement: S5 Fig — (TIFF) [file pone.0152366.s005.TIFF]
