# Supplementary material for: Transcriptomic Characterization of Tambaqui (Colossoma macropomum, Cuvier, 1818) Exposed to Three Climate Change Scenarios
Source: PLoS One. 2016 Mar 28;11(3):e0152366. doi: 10.1371/journal.pone.0152366 (PMC4809510; doi:10.1371/journal.pone.0152366)
Supplement: S1 Table — Up-regulated genes are shown with positive values, and down-regulated genes are shown with negative values. (DOCX) [file pone.0152366.s006.docx]

Table S1: The list of differentially expressed genes (Log2FC) of tambaqui after five days of exposure to B1, A1B and A2 climate scenarios. Up- regulated genes are shown with positive values and down-regulated genes are shown with negative values.

| **Five days of exposition** | | | |
| --- | --- | --- | --- |
| **Gene symbol** | **B1 Scenario** | **A1B Scenario** | **A2 Scenario** |
| A2ML1 (5 of 12) | 2.174 | 1.288 | 1.153 |
| AC024175.1 | -1.415 | -4.791 | -1.642 |
| AC024175.11 | -1.428 | -1.764 | -2.903 |
| AC024175.13 | -1.237 | -1.477 | -2.283 |
| AC024175.14 | -3.587 | -93.539 | -93.539 |
| AC024175.17 | -1.328 | -1.727 | -1.98 |
| AC024175.6 | -1.112 | -2.221 | -2.282 |
| AC024175.7 | 4.638 | 4.605 | -31.24 |
| AC024175.8 | 2.303 | -97.023 | -1.131 |
| AC024175.9 | 2.637 | 1.029 | 1.208 |
| acta1b | -2.208 | -1.014 | -1.339 |
| actc1b | -1.591 | 1.095 | -1.184 |
| ACVR2B (1 of 2) | 2.616 | 4.631 | 7.597 |
| adss | -5.739 | 1.096 | -1.008 |
| AL929108.1 | 5.227 | -21.065 | -21.065 |
| AL935186.3 | -1.797 | -5.291 | -2.062 |
| aldoaa | -3.935 | -2.237 | -1.306 |
| aldoab | -4.239 | -1.361 | -2.124 |
| anp32b | 1.847 | -1.259 | -1.349 |
| arhgef9b_2 | -4.073 | -4.64 | -4.688 |
| arl8bb | -2.233 | 2.305 | 2.611 |
| arpc3 | -1.198 | -2.673 | 1.197 |
| atg4a | 2.055 | 5.596 | -1.414 |
| atp2a1l | -3.825 | -1.1 | -1.265 |
| atp2a2b | -4.553 | 1.79 | -1.161 |
| atp5a1 | -2.81 | -1.112 | -1.089 |
| ATP5B | -2.994 | -1.191 | 1.026 |
| atp5c1 | -2.585 | -1.231 | -1.684 |
| atp5d | -1.67 | -1.662 | -1.92 |
| atp5h | 1.016 | -1.21 | -1.697 |
| bhmt | -3.513 | -2.274 | -1.077 |
| btf3 | -1.598 | 1.325 | 2.251 |
| BX470224.1 | 1.147 | -1.627 | 3.412 |
| BX548011.3_1 | 2.364 | 1.135 | 1.333 |
| CABZ01055869.1 | -1.278 | -3.28 | -148.539 |
| CABZ01061343.1 | 93.445 | 1 | 1 |
| CABZ01076182.1 | -32.265 | -32.265 | -32.265 |
| CABZ01077555.1 | 6.606 | 27.918 | 15.989 |
| capn1a | -8.046 | -2.627 | -4.7 |
| ccng1 | -1.115 | 2.39 | 3.601 |
| cdh23_2 | -1.394 | -1.677 | -2.194 |
| cep89 | -2.004 | -1.293 | 2.085 |
| ckma | -1.733 | -1.475 | -1.518 |
| ckmb | -2.322 | -1.358 | -1.318 |
| ckmt1 | -2.52 | -1.109 | -1.11 |
| cmc4 | -1.249 | -5.794 | -4.401 |
| cox4i2 | -11.376 | -1.51 | -3.53 |
| cox7c | -1.282 | -3.077 | 1.315 |
| cox8b | -2.699 | -1.772 | -2.402 |
| cpn1_2 | 3.051 | 2.051 | -1.097 |
| CR354374.1 | -1.54 | 1.041 | -2.245 |
| CR356235.1 | 48.411 | 1 | 1 |
| CR381646.1 | -1.402 | -45.81 | -2.241 |
| CR450736.2 | -3.646 | 1.22 | 1.482 |
| CR854899.1 | 255.709 | 355.202 | 1 |
| CR936200.1_1 | 2.612 | 1.146 | -1.216 |
| creb3l3l | -2.466 | 1.11 | 7.259 |
| cstf2_1 | 3.568 | 1.012 | 1.209 |
| CU459159.1 | 1 | 38.331 | 1 |
| CU570683.1 | 10.145 | 19.967 | 23.423 |
| CU633479.5_1 | -3.952 | -1.253 | 2.269 |
| CU929274.1 | 1.002 | -81.378 | -81.378 |
| cxcr3.1_1 | 2.076 | 1.398 | 1.433 |
| dbi | 11.457 | 12.846 | 5.819 |
| ddost | 2.451 | 2.01 | 3.198 |
| desma | 1.289 | 2.062 | 1.941 |
| dicp1.1 | -1.347 | -3.469 | -1.259 |
| dlg3_2 | 3.646 | 1.598 | 1.654 |
| dnaja2 | -2.864 | -1.376 | -1.415 |
| dnajc7_2 | 5.065 | 5.969 | 2.234 |
| dre-let-7a-1 | 1.712 | -103.825 | -1.925 |
| dre-let-7a-4 | -1.427 | -61.669 | 2.254 |
| dre-let-7c-2 | 2.817 | 3.721 | 5.532 |
| dre-let-7d-1 | 100.024 | 1 | 49.639 |
| dre-let-7d-2 | 84.961 | 64.599 | 57.037 |
| dre-let-7f | 16.173 | 1 | 28.652 |
| dre-mir-125a-2 | 42.165 | 1 | 1 |
| dre-mir-193a-2 | 45.976 | 1 | 1 |
| dre-mir-206-2 | 1.734 | -1.104 | 1.751 |
| dre-mir-20b | -60.971 | -60.971 | -3.199 |
| dre-mir-21-2 | 1 | 15.671 | 53.275 |
| dre-mir-301c | 1 | 1 | 36.96 |
| dre-mir-363 | -3.988 | -1.794 | -59.262 |
| dre-mir-731 | 58.59 | 1 | 1 |
| edil3 | 3.538 | 10.618 | 5.203 |
| eef2l2 | -1.878 | -1.369 | 1.107 |
| efr3a_2 | 2.388 | 1.21 | 1.001 |
| eif3f | 2.113 | 1.264 | 1.135 |
| eif3m | -1.883 | 1.092 | 1.115 |
| eif4ebp3l | -1.335 | -1.432 | -2.751 |
| eno1a | -5.643 | -7.741 | -1.309 |
| eno3 | -3.388 | -2.06 | -1.611 |
| ENSDARG00000041884 | 3.247 | 2.64 | 1.9 |
| ENSDARG00000071292 | -2.688 | -1.329 | 2.461 |
| ENSDARG00000088545 | 95.301 | 14.619 | 5.694 |
| ENSDARG00000091099 | 1.436 | 5.715 | 5.346 |
| FBXL6 | 2.491 | -1.74 | -2.416 |
| FER1L6 | -15.823 | -3.65 | -8.985 |
| fh | -3.469 | -1.119 | -1.194 |
| FP102784.1 | 6.397 | 3.556 | -4.975 |
| gapdh | -3.189 | -1.321 | -1.466 |
| gapdhs | -3.272 | -1.856 | -1.463 |
| gcdhl | -5.069 | -1.45 | 1.68 |
| gpib | -10.688 | -1.203 | -1.835 |
| her9 | -1.074 | 2.041 | 1.606 |
| hip1ra | -8.784 | -2.096 | -3.488 |
| hmgb1a | -1.722 | -2.413 | -1.931 |
| HSCB | 1.76 | 2.209 | 1.844 |
| hsp90aa1.1 | 1.124 | 2.389 | 3.465 |
| ical1_1 | 1.382 | 2.116 | 1.047 |
| IDH2 | -1.85 | -1.265 | -1.647 |
| IFT43_2 | 35.882 | 1 | 19.915 |
| kcnab1_1 | 1.989 | -1.455 | -1.349 |
| kif22 | 19.187 | -4.436 | 5.065 |
| lama2_1 | 1.755 | -1.105 | -1.176 |
| lhfpl2a | 1.406 | -1.661 | -2.369 |
| lhfpl2b_2 | 15.019 | 2.976 | 4.816 |
| lsm12b | -5.189 | -1.54 | -1.35 |
| map4k2l_2 | 3.271 | -1.055 | -1.501 |
| me2_1 | 1.581 | -1.146 | -1.641 |
| Metazoa_SRP_2 | 59.604 | 14.79 | 6.115 |
| Metazoa_SRP_56 | 1 | 29.74 | 1 |
| mhc1zaa_2 | -1.089 | -1.14 | -3.138 |
| mospd1 | -9.36 | -8.567 | -3.124 |
| MTHFS | 7.442 | 4.056 | 24.842 |
| MUSTN1 | 5.369 | 14.364 | 38.182 |
| MYH13 (5 of 11) | -2.561 | 1.158 | 1.808 |
| myhz1.3 | -3.906 | 1.23 | 1.332 |
| myl1 | -1.134 | 1.901 | 1.448 |
| myl10 | -2.568 | 1.361 | 8.015 |
| MYL2 (1 of 2) | -4.99 | 2.818 | 6.899 |
| MYL3 | -3.177 | 2.62 | 11.621 |
| mylpfb | -3.199 | 1.843 | 3.341 |
| mylz3 | -2.075 | -1.106 | -1.051 |
| ndufs4 | -4.021 | -3.862 | -1.405 |
| ngfrb_1 | -1.212 | 1.242 | -2.358 |
| nipsnap3 | -5.069 | -1.767 | -1.729 |
| nmrk2 | 5.359 | 7.937 | 5.455 |
| nnt | 1.698 | 3.589 | 1.205 |
| nphp3 | -2.687 | -2.743 | -4.803 |
| nudc | 1.23 | -2.24 | 1.031 |
| OTTDARG00000019147_1 | -2.5 | -10.402 | -2.181 |
| pabpc4 | -1.928 | -1.312 | 1.077 |
| PET100 | 1.68 | 1.199 | 2.893 |
| pfdn2 | -1.378 | 1.621 | 3.999 |
| pgk1 | -3.3 | -1.019 | -1.234 |
| pgm1 | -3.206 | -1.725 | -1.509 |
| pkmb | -5.666 | -1.415 | -1.197 |
| pomp | -2.064 | -2.324 | -2.348 |
| psma1 | -2.726 | -1.704 | -4.381 |
| psma2 | 1.424 | -1.368 | -4.261 |
| psma3 | -3.814 | -2.145 | -2.055 |
| psmb3 | -3.859 | -2.404 | -2.806 |
| pthlhb_2 | -3.219 | -2.061 | -7.976 |
| ptpn9b | -5.287 | 1.078 | -1.453 |
| pvalb1 | 1.039 | 1.106 | -1.381 |
| rab10 | -2.238 | -5.857 | -2.148 |
| rbm19 | -3.354 | 1.194 | -1.393 |
| rn7sk | -1.474 | -1.728 | -2.217 |
| rpl11 | -1.605 | 1.118 | -1.449 |
| rpl22 | 1.583 | 2.034 | 1.426 |
| rpl35 | -1.181 | -1.555 | -1.083 |
| rpl6 | 3.207 | 7.783 | 35.03 |
| rpl7 | -2.356 | -1.515 | -1.314 |
| rps10 | 2.208 | 1.956 | 2.97 |
| rps27.2_1 | -1.288 | -1.015 | -1.63 |
| rps29 | 10.681 | 4.009 | 6.317 |
| rps3 | -1.629 | -1.168 | 1.403 |
| rpsa | -1.584 | -1.487 | 1.097 |
| rtn2a | -3.465 | -2.398 | -1.914 |
| scn1a_2 | -4.251 | -3.138 | -3.262 |
| sdhb | -5.083 | -2.757 | -1.641 |
| si:ch1073-140o9.2 | -2.207 | -1.129 | 3.627 |
| si:ch211-122a17.5 | 1 | 47.534 | 65.228 |
| si:ch211-122l24.5 | -5.539 | 2.846 | 6.512 |
| si:ch211-130l20.1 | 52.716 | 1 | 1 |
| si:ch211-238p8.23 | 4.216 | -1.276 | 1.783 |
| si:ch211-241d24.2 | 1.048 | -32.529 | -3.761 |
| si:ch211-242m24.8 | 1 | 1 | 26.102 |
| si:ch211-37e10.1 | 4.32 | 1.485 | 1.301 |
| si:ch211-39k3.2 | 1.087 | -3.194 | -1.596 |
| si:ch211-59c24.1 | -1.313 | -8.559 | -3.319 |
| si:ch73-191k20.4 | 34.213 | 9.716 | 4.468 |
| si:ch73-346o4.2 | 6.973 | 28.429 | 7.737 |
| si:dkey-15h8.10 | -5.996 | -1.016 | 1.325 |
| si:dkey-17m8.1 | 2.131 | -1.224 | 1.208 |
| si:dkey-186k21.2 | 37.868 | 26.195 | 7.779 |
| si:dkey-234n3.3 | -23.253 | -23.253 | -23.253 |
| si:dkey-267i17.3 | 5.092 | 36.106 | 6.167 |
| si:dkey-34e4.1 | 12.263 | 5.353 | 1.909 |
| si:dkey-52l6.2 | 26.459 | 10.276 | 12.651 |
| si:dkey-86e18.1 | 1.433 | 1.914 | 1.194 |
| slc25a4 | -1.991 | -2.015 | -1.596 |
| smarcal1 | -3.323 | -2.32 | -6.086 |
| smyhc1 | -1.886 | 1.282 | 2.772 |
| SNORA17 | 38.671 | 1 | 1 |
| SNORA9_2 | -2.294 | -78.718 | -78.718 |
| snoZ178 | 37.465 | 19.204 | 198.783 |
| SPTBN4 (1 of 2) | 10.132 | 1.757 | -1.384 |
| ssr3_2 | -1.897 | -33.199 | -3.507 |
| stxbp1a | -1.917 | 2.271 | -1.065 |
| tank_1 | -1.127 | -1.982 | -2.373 |
| TARS2 (1 of 2) | -5.619 | -3.116 | -2.525 |
| thoc7 | 4.993 | 2.051 | 3.19 |
| tmem167b_1 | 2.547 | 1.59 | 1.113 |
| tmem55a | 710.93 | -3.561 | -3.561 |
| TNFRSF9 (2 of 2) | -4.796 | 5.832 | 56.282 |
| tnnc1a | 1.565 | 2.592 | 30.773 |
| tnnc1b | -4.037 | 1.666 | 8.398 |
| TNNC2 (2 of 2) | 5.244 | 17.074 | 10.416 |
| tnni2a.1 | -1.439 | -1.703 | -1.465 |
| tnni2a.4 | -1.034 | 2.789 | 1.482 |
| tnnt1 | 4.144 | 4.107 | 3.938 |
| tomm20b | -3.047 | -2.328 | -2.057 |
| tpi1a | -5.416 | -1.344 | -1.155 |
| tpm2 | -1.133 | 2.089 | 6.497 |
| tpm3 | -1.308 | 3.024 | 3.663 |
| tpma | -2.409 | 1.374 | 1.275 |
| txndc12 | -5.319 | 1.195 | -1.248 |
| U1_101 | 24.934 | 1 | 1 |
| U3_15 | -1.449 | -9.113 | -22.542 |
| U6_2 | -3.86 | -65.905 | -65.905 |
| ubxn6 | -2.715 | 1.023 | -1.272 |
| ugt2b6_1 | 1.85 | -1.174 | -1.158 |
| ungb | 8.259 | 22.533 | 1.342 |
| v2rh25p | 29.068 | 4.745 | 11.195 |
| Vault_4 | 38.671 | 1 | 1 |
| vcp | -5.996 | -1.65 | -1.757 |
| WDR70 (1 of 2) | 36.128 | 8.439 | 4.019 |
| xrcc5_1 | -1.881 | -1.81 | -3.714 |
| zak_1 | 1.664 | 4.728 | 1.176 |
| zgc:153129 | -27.503 | -1.784 | -4.617 |
| zgc:165409_2 | -1.435 | -5.547 | -14.411 |
| zgc:65894 | 1.448 | 3.02 | 11.202 |
| zgc:86598 | -4.076 | -1.197 | -1.06 |
| zgc:91860 | 1.325 | -1.437 | -1.855 |
