# Supplementary material for: Transcriptomic Characterization of Tambaqui (Colossoma macropomum, Cuvier, 1818) Exposed to Three Climate Change Scenarios
Source: PLoS One. 2016 Mar 28;11(3):e0152366. doi: 10.1371/journal.pone.0152366 (PMC4809510; doi:10.1371/journal.pone.0152366)
Supplement: S2 Table — Up-regulated genes are shown with positive values, and down-regulated genes are shown with negative values. (DOCX) [file pone.0152366.s007.docx]

Table S2: The list of differentially expressed genes (Log2FC) of tambaqui after fifteen days of exposure to B1, A1B and A2 climate scenarios. Up- regulated genes are shown with positive values and down-regulated genes are shown with negative values.

| **Fifteen days of exposition** | | | |
| --- | --- | --- | --- |
| **Gene symbol** | **B1 Scenario** | **A1B Scenario** | **A2 Scenario** |
| abca12 | 3.537 | -1.935 | 1.567 |
| abce1 | -1.69 | -11.541 | -1.912 |
| AC024175.1 | -15.542 | 1.065 | -2.27 |
| AC024175.11 | -2.292 | 1.608 | -1.847 |
| AC024175.13 | -3.738 | -1.179 | -2.237 |
| AC024175.17 | -3.653 | -1.44 | -2.323 |
| AC024175.4 | -1.909 | -1.07 | -1.259 |
| AC024175.6 | -3.31 | 1.44 | -2.019 |
| AC024175.9 | -4.043 | 2.935 | 2.081 |
| actc1a | -1.994 | -3.548 | -1.63 |
| actn2 | -1.031 | -5.925 | -1.459 |
| actr3b | 1.056 | -5.167 | -1.749 |
| ACVR2B (1 of 2) | -1.118 | -7.484 | -3.065 |
| AL845481.1 | -2.623 | -1.028 | -58.662 |
| AL929192.1 | -7.423 | -42.884 | -42.884 |
| AL935186.3 | -1.314 | 1.367 | -5.339 |
| AL935186.6 | -1.33 | 1.919 | 1.234 |
| aldocb | -2.517 | -6.145 | -5.47 |
| ampd3b | -1.358 | -2.252 | -3.125 |
| arpc3 | 1.037 | -2.695 | -1.643 |
| atp5l | -1.855 | 1.084 | -1.148 |
| atpif1b | 2.46 | 18.894 | 14.318 |
| bcl2_2 | -1.227 | 1.658 | 1.123 |
| bin3 | 31.527 | 1 | 34.876 |
| BIN3 (2 of 2) | 33.668 | 1 | 4.962 |
| btf3 | -1.77 | -10.563 | -2.192 |
| BX005423.2 | -37.367 | -37.367 | -1.534 |
| BX322605.3 | 2.886 | 25.793 | 3.364 |
| BX548011.3_1 | -1.591 | -1.122 | -1.171 |
| BX890616.1 | -1.189 | 1.521 | 3.769 |
| CABZ01025931.1 | -5.754 | 1.713 | 2.5 |
| CABZ01041280.1 | 27.687 | 11.276 | 25.724 |
| calm2a | -1.418 | -2.376 | -2.507 |
| calm3a | -1.919 | -2.874 | -1.567 |
| casq1b | 1.184 | -2.355 | -1.571 |
| ccng1 | -2.152 | -3.466 | -8.178 |
| chchd10 | -2.673 | -6.581 | 1.006 |
| clasrp | 38.746 | 10.065 | -1.545 |
| clk4a | 1.051 | -1.673 | 3.154 |
| col5a3a_1 | -1.94 | 3.168 | 2.329 |
| cops3 | -1.927 | -5.353 | -1.653 |
| cox5b2 | -5.997 | -2.881 | -48.526 |
| CR318660.2 | 49.585 | 1 | 1 |
| CR354374.1 | -4.112 | 1.175 | -1.227 |
| CR354382.1 | 1 | 1 | 45.405 |
| CR354430.2 | -65.91 | -2.337 | -65.91 |
| CR790365.1 | 18.206 | 72.803 | 60.316 |
| CR847944.3 | 1 | 159.101 | 1 |
| CR854899.1 | 191.493 | 1 | 25.381 |
| creb3l3l | -8.451 | -6.314 | -1.816 |
| ctnnd1_2 | -1.109 | 2.237 | 1.592 |
| CU633479.5_1 | 3.09 | -1.05 | 1.361 |
| CU984584.1 | 4.135 | 6.537 | 2.41 |
| cxcl-c5c | 5.163 | 24.858 | 5.659 |
| ddx56 | -6.631 | -17.802 | -2.089 |
| desma | -1.975 | -3.991 | -1.797 |
| desmb | 1.198 | -3.035 | 1.199 |
| dicp1.1 | -2.982 | -2.414 | -4.124 |
| dre-let-7a-1 | -1.093 | -1.015 | -89.706 |
| dre-let-7a-2 | 1.349 | 2.924 | -76.928 |
| dre-let-7a-3 | -2.215 | -2.946 | -1.645 |
| dre-let-7a-4 | -3.621 | -4.957 | -139.727 |
| dre-let-7a-5 | 1.154 | -39.845 | -1.159 |
| dre-let-7d-1 | -34.74 | -1.293 | -34.74 |
| dre-mir-125b-2 | -2.519 | -56.941 | -2.22 |
| dre-mir-130b | 20.493 | 69.759 | 99.54 |
| dre-mir-142a | 1 | 64.428 | 1 |
| dre-mir-15a-1 | 1.129 | -37.736 | -37.736 |
| dre-mir-181a-1 | -39.98 | 1.483 | -39.98 |
| dre-mir-206-2 | -1.754 | -2.802 | -1.241 |
| dre-mir-210 | -1.186 | -88.136 | -88.136 |
| dre-mir-2188 | 34.612 | 1 | 20.637 |
| dre-mir-218b | 1 | 65.136 | 1 |
| dre-mir-222b | -34.746 | -1.156 | -34.746 |
| dre-mir-363 | 21.539 | -1.706 | 2.082 |
| dre-mir-454b | -2.149 | -66.921 | 114.024 |
| eef1a1a | -1.265 | -2.33 | -3.688 |
| eef1a1l1 | -1.441 | -2.164 | -1.488 |
| eef1a2 | -1.647 | -8.341 | -1.48 |
| efr3a_2 | 1.649 | 2.905 | 1.509 |
| eif2s1b | -5.704 | -1.48 | -1.299 |
| eif4a3 | -1.229 | -4.222 | -1.423 |
| eif4eb | -1.364 | -26.474 | -4.209 |
| eif4ebp3l | -1.466 | -3.262 | -1.615 |
| ENSDARG00000073911 | -1.029 | -29.118 | 1.558 |
| ENSDARG00000086253 | -6.631 | 9.189 | -6.631 |
| ENSDARG00000088545 | -6.245 | 9.845 | -6.245 |
| ENSDARG00000091099 | 1.546 | -7.45 | -2.004 |
| ENSDARG00000094466 | -1.522 | -23.501 | -2.571 |
| ENSDARG00000096531 | -1.257 | -4.726 | 1.246 |
| FER1L6 | -1.46 | -5.087 | -2.849 |
| FIS1 | -3.74 | -6.596 | -5.249 |
| GABARAP (2 of 2) | 1.375 | -3.316 | -1.028 |
| heatr5a_1 | -1.889 | 3.461 | 1.054 |
| her9 | 2.119 | 1.177 | 3.539 |
| hmgb1a | -1.842 | -4.908 | -1.181 |
| hoxd3a | 55.467 | -1.749 | -1.749 |
| hsp90aa1.2 | 1.34 | -18.356 | -1.648 |
| hypk | -2.63 | -2.581 | -2.02 |
| iars | -2.312 | -5.766 | -1.64 |
| kdm7ab | 23.681 | -1.296 | -1.077 |
| KLHDC8A | 57.146 | -2.482 | 13.435 |
| lamtor2 | 12.788 | 4.297 | 29.943 |
| ldha | -1.126 | -3.705 | -1.721 |
| map4k2l_2 | -3.067 | -3.051 | -2.419 |
| mdh2 | -1.564 | -1.499 | -1.359 |
| Metazoa_SRP_23 | -12.267 | -4.251 | -1.049 |
| Metazoa_SRP_24 | -23.171 | -2.379 | -23.171 |
| Metazoa_SRP_38 | 3.863 | 9.774 | 1.193 |
| mob4 | 1.008 | -25.959 | -2.522 |
| mpc1 | 1.177 | -4.901 | -2.446 |
| MTBP | -1.828 | 3.661 | 1.793 |
| MYH13 (10 of 11) | 1.039 | -5.111 | -3.712 |
| myl10 | -9.559 | -3.748 | -2.687 |
| MYL3 | -8.388 | -1.732 | -2.259 |
| mylpfb | -1.385 | -5.255 | -2.677 |
| naa10 | -2.792 | -5.044 | -1.239 |
| ndufa12 | -5.742 | 1.231 | -1.911 |
| ndufaf6 | -1.094 | 4.101 | 1.47 |
| NDUFC2 | -1.417 | 2.064 | 1.591 |
| ndufv1 | -1.741 | -2.859 | -1.543 |
| nwd1_2 | -3.272 | -3.055 | -1.616 |
| ostc | -4.648 | -2.55 | -1.561 |
| OXR1 (2 of 2) | -1.689 | -40.804 | -2.167 |
| parvb | -1.038 | -3.599 | -1.242 |
| pdap1b | -1.609 | -4.138 | -1.99 |
| pdlim7 | 1.861 | -4.67 | 1.109 |
| pfdn2 | -2.897 | -9.682 | -2.246 |
| prkag3b | 4.991 | -1.416 | 7.286 |
| prmt1 | -2.216 | -24.793 | -2.541 |
| rbm8a | -1.403 | -3.084 | -1.533 |
| rheb | -1.805 | -2.829 | -2.171 |
| rn7sk | -4.028 | -1.173 | -2.461 |
| rock2a | 11.765 | 1.726 | 1.192 |
| rpl13 | -3.677 | -2.207 | -1.304 |
| rpl14 | -1.774 | 1.474 | 1.28 |
| rpl18a | -2.18 | -2.574 | -1.858 |
| rpl19 | -1.934 | 1.039 | -1.398 |
| rpl23 | -2.051 | 1.253 | 1.163 |
| rpl28 | -1.897 | 1.05 | -1.12 |
| rpl3 | -2.332 | -1.16 | -1.229 |
| rpl32 | -1.71 | 1.888 | 1.152 |
| rpl35 | -4.35 | -1.042 | -1.935 |
| rpl36a | -1.792 | 1.62 | -1.017 |
| rpl5a | -1.909 | 1.749 | 1.143 |
| rpl7 | -1.993 | -1.662 | -1.163 |
| rplp2 | -1.488 | 1.702 | 1.172 |
| rps10 | -2.453 | -1.032 | -1.289 |
| rps16 | -1.286 | 1.642 | -1.098 |
| rps23 | -1.254 | 1.468 | -1.041 |
| rps24 | -3.95 | 2.206 | -1.015 |
| rps26 | -1.936 | 1.071 | 1.04 |
| rps26l | -2.053 | 1.146 | -1.511 |
| rps27.1 | -1.497 | -1.127 | -1.404 |
| rps3a | -1.777 | 1.184 | -1.136 |
| rpsa | -1.73 | -1.187 | -1.177 |
| rtn2b | -1.062 | -9.541 | -1.558 |
| scn1a_2 | 1.89 | 3.587 | 1.951 |
| SHISA7 (1 of 2) | -1.343 | -3.898 | -1.645 |
| si:ch1073-140o9.2 | 3.025 | -1.568 | -1.657 |
| si:ch211-37e10.1 | 1.001 | 3.72 | 1.363 |
| si:ch211-39k3.2 | -1.559 | -3.201 | -2.088 |
| si:ch211-59c24.1 | -8.472 | 1.457 | 2.249 |
| si:ch73-106n3.1 | -5.941 | -1.072 | 2.229 |
| si:dkey-111b14.2 | -5.253 | -1.36 | -2.48 |
| si:dkey-151g10.6 | -2.099 | 1.001 | -1.713 |
| si:dkey-153m14.1 | -1.786 | 1.146 | -1.255 |
| si:dkey-16m19.1_2 | -1.739 | -51.973 | -1.092 |
| si:dkey-1b17.10 | 5.22 | -2.277 | 2.319 |
| si:dkey-240k8.2 | 31.051 | 1 | 1 |
| si:dkey-41e15.4 | -4.831 | -2.098 | 1.263 |
| si:dkey-86e18.1 | 1.852 | 2.387 | 2.311 |
| si:dkeyp-84g1.2 | 22.863 | 1 | 9.819 |
| slc25a4 | -1.292 | -2.418 | -2.101 |
| slc4a4b | -2.735 | 1.476 | -1.077 |
| SMIM4 | 5.317 | -14.296 | -1.511 |
| SNORA3_4 | 43.599 | 1 | 1 |
| SNORD31_2 | 1.428 | 3.404 | -91.533 |
| snR56 | -564.829 | -7.481 | -564.829 |
| snrpb | -4.447 | -2.482 | -1.307 |
| soul4_1 | 2.382 | -1.406 | 7.944 |
| sparc | -1.79 | -15.228 | -1.666 |
| spcs1 | -2.833 | -2.171 | -5.309 |
| sssca1 | -8.74 | -6.637 | -1.885 |
| tcp1 | -1.859 | -3.275 | -1.738 |
| thoc7 | -2.564 | -13.11 | -1.101 |
| tmem38a | -1.324 | -5.143 | -2.999 |
| tmsb4x | -1.058 | -2.424 | 1.02 |
| tnnc1a | -10.433 | -2.413 | 1.055 |
| tnnc1b | -4.96 | -2.945 | -1.066 |
| tnnt3b | -2.307 | -1.8 | -1.26 |
| tomm20b | -1.877 | -4.951 | -2.17 |
| TSG101 (3 of 3) | 13.702 | 10.623 | 28.24 |
| U1_25 | 1 | 1 | 26.211 |
| U2_9 | 1.302 | 2.822 | 1.493 |
| u2af1 | -2.012 | -5.379 | -1.996 |
| U3_27 | 1.08 | 1.551 | 6.55 |
| U6_234 | -150.018 | 2.364 | -2.515 |
| U8_8 | -1.279 | 3.026 | -1.573 |
| ugt2b6_1 | -1.053 | 2.456 | 1.431 |
| uqcrc1 | -1.442 | -6.095 | -2.971 |
| utp18 | -3.263 | -7.462 | -1.941 |
| vbp1 | -2.723 | -3.968 | -6.275 |
| WWP1 | 1.43 | -5.171 | -2.085 |
| ybx1 | -2.909 | -5.797 | -1.78 |
| zgc:113295_1 | -1.519 | 1.851 | 1.006 |
| zgc:171759_4 | -1.307 | 3.092 | 1.413 |
| zgc:65894 | -1.613 | -12.707 | -3.023 |
| ZP2 (1 of 4) | -1.974 | -1.873 | -2.536 |
