# Supplementary material for: Transcriptomic Characterization of Tambaqui (Colossoma macropomum, Cuvier, 1818) Exposed to Three Climate Change Scenarios
Source: PLoS One. 2016 Mar 28;11(3):e0152366. doi: 10.1371/journal.pone.0152366 (PMC4809510; doi:10.1371/journal.pone.0152366)
Supplement: S3 Table — (DOCX) [file pone.0152366.s008.docx]

Table S3: Protein interactions of tambaqui after five and fifteen days of B1, A1B and A2 climate scenarios exposure using STRING software (v. 10).

| **node1** | **node2** | **node1_string_id** | **node2_string_id** | **node1_external_id** | **node2_external_id** | **neighborhood** | **fusion** | **cooccurence** | **homology** | **coexpression** | **experimental** | **knowledge** | **textmining** | **combined_score** |
| --- | --- | --- | --- | --- | --- | --- | --- | --- | --- | --- | --- | --- | --- | --- |
| eif2s1 | rps10 | 1488921 | 1485542 | ENSDARP00000068470 | ENSDARP00000045900 | 0 | 0 | 0 | 0 | 0.117 | 0 | 0.900 | 0 | 0.907 |
| rpl6 | rps27.1 | 1493092 | 1484170 | ENSDARP00000091899 | ENSDARP00000029079 | 0 | 0 | 0 | 0 | 0.977 | 0.395 | 0.900 | 0.542 | 0.999 |
| rbm8a | rpl5a | 1483952 | 1481770 | ENSDARP00000026575 | ENSDARP00000006085 | 0 | 0 | 0 | 0 | 0.098 | 0 | 0.900 | 0.064 | 0.908 |
| myl2 | smyhc1 | 1503455 | 1486922 | ENSDARP00000116241 | ENSDARP00000056852 | 0 | 0 | 0 | 0 | 0.085 | 0.288 | 0.900 | 0.388 | 0.954 |
| rps24 | rpl18a | 1493019 | 1484877 | ENSDARP00000091586 | ENSDARP00000038658 | 0 | 0 | 0 | 0 | 0.919 | 0.873 | 0.900 | 0.197 | 0.999 |
| rpl32 | rps10 | 1487450 | 1485542 | ENSDARP00000060004 | ENSDARP00000045900 | 0 | 0 | 0 | 0 | 0.961 | 0 | 0.900 | 0.118 | 0.996 |
| rpl19 | rps27.2 | 1498085 | 1489628 | ENSDARP00000105649 | ENSDARP00000072300 | 0 | 0 | 0 | 0 | 0.972 | 0.450 | 0.900 | 0.330 | 0.998 |
| rpl22 | rpl14 | 1501438 | 1486227 | ENSDARP00000111487 | ENSDARP00000052528 | 0 | 0 | 0 | 0 | 0.959 | 0.973 | 0.900 | 0.654 | 0.999 |
| tnnt3b | tpm3 | 1497962 | 1481594 | ENSDARP00000105443 | ENSDARP00000004352 | 0 | 0 | 0 | 0 | 0.436 | 0.378 | 0.900 | 0.857 | 0.994 |
| rpl22 | eif2s1 | 1501438 | 1488921 | ENSDARP00000111487 | ENSDARP00000068470 | 0 | 0 | 0 | 0 | 0.134 | 0 | 0.900 | 0.237 | 0.928 |
| rpsa | spcs1 | 1506145 | 1490634 | ENSDARP00000123183 | ENSDARP00000076814 | 0 | 0 | 0 | 0 | 0.129 | 0 | 0.900 | 0 | 0.909 |
| aldocb | gpib | 1483738 | 1482688 | ENSDARP00000024492 | ENSDARP00000014578 | 0 | 0 | 0 | 0 | 0.691 | 0 | 0.800 | 0.460 | 0.963 |
| rpsa | rps27.2 | 1506145 | 1489628 | ENSDARP00000123183 | ENSDARP00000072300 | 0 | 0 | 0 | 0 | 0.995 | 0.797 | 0.900 | 0.330 | 0.999 |
| tnnc1a | tpm3 | 1483843 | 1481594 | ENSDARP00000025541 | ENSDARP00000004352 | 0 | 0 | 0 | 0 | 0 | 0.127 | 0.900 | 0.159 | 0.920 |
| rpl6 | rps10 | 1493092 | 1485542 | ENSDARP00000091899 | ENSDARP00000045900 | 0 | 0 | 0 | 0 | 0.883 | 0.254 | 0.900 | 0.390 | 0.994 |
| rps24 | eif4a3 | 1493019 | 1484026 | ENSDARP00000091586 | ENSDARP00000027276 | 0 | 0 | 0 | 0 | 0.147 | 0.470 | 0.900 | 0 | 0.950 |
| rps3 | rplp2 | 1488779 | 1483852 | ENSDARP00000067802 | ENSDARP00000025616 | 0 | 0 | 0 | 0 | 0.999 | 0.582 | 0 | 0.605 | 0.999 |
| rps3a | rbm8a | 1486104 | 1483952 | ENSDARP00000051762 | ENSDARP00000026575 | 0 | 0 | 0 | 0 | 0.118 | 0 | 0.900 | 0 | 0.908 |
| rps3 | rps23 | 1488779 | 1484618 | ENSDARP00000067802 | ENSDARP00000035273 | 0.493 | 0 | 0 | 0 | 0.999 | 0.978 | 0.900 | 0.369 | 0.999 |
| rps3a | rps23 | 1486104 | 1484618 | ENSDARP00000051762 | ENSDARP00000035273 | 0 | 0 | 0 | 0 | 0.998 | 0.978 | 0.900 | 0.360 | 0.999 |
| acvr2b | smurf1 | 1484100 | 1481527 | ENSDARP00000028046 | ENSDARP00000003721 | 0 | 0 | 0 | 0 | 0 | 0.072 | 0.900 | 0.238 | 0.924 |
| rps26 | rpl5a | 1497897 | 1481770 | ENSDARP00000105328 | ENSDARP00000006085 | 0 | 0 | 0 | 0 | 0.995 | 0.141 | 0.900 | 0.934 | 0.999 |
| EIF3F | eif3m | 1495369 | 1483698 | ENSDARP00000099664 | ENSDARP00000024129 | 0 | 0 | 0 | 0 | 0.738 | 0.978 | 0 | 0.542 | 0.997 |
| LOC567740 | mylz3 | 1505894 | 1483092 | ENSDARP00000122502 | ENSDARP00000018197 | 0 | 0 | 0 | 0 | 0 | 0.202 | 0.900 | 0.209 | 0.931 |
| spcs1 | rpl3 | 1490634 | 1481524 | ENSDARP00000076814 | ENSDARP00000003700 | 0 | 0 | 0 | 0 | 0.108 | 0 | 0.900 | 0 | 0.906 |
| EIF3F | rpl28 | 1495369 | 1483702 | ENSDARP00000099664 | ENSDARP00000024189 | 0 | 0 | 0 | 0 | 0.865 | 0 | 0.900 | 0 | 0.986 |
| aldoab | eno2 | 1485195 | 1484405 | ENSDARP00000042199 | ENSDARP00000032456 | 0 | 0 | 0 | 0 | 0.750 | 0.070 | 0.800 | 0.459 | 0.971 |
| hsp90aa1.1 | zgc:65894 | 1483481 | 1481340 | ENSDARP00000022302 | ENSDARP00000002175 | 0 | 0 | 0 | 0 | 0.069 | 0.073 | 0.900 | 0.238 | 0.925 |
| atp2a1l | pvalb1 | 1494243 | 1486619 | ENSDARP00000096674 | ENSDARP00000055061 | 0 | 0 | 0 | 0 | 0.894 | 0.079 | 0 | 0.362 | 0.932 |
| rpl18a | rps27.1 | 1484877 | 1484170 | ENSDARP00000038658 | ENSDARP00000029079 | 0 | 0 | 0 | 0 | 0.997 | 0 | 0.900 | 0.166 | 0.999 |
| rbm19 | mphosph10 | 1487837 | 1483869 | ENSDARP00000062458 | ENSDARP00000025759 | 0 | 0 | 0 | 0 | 0.763 | 0.788 | 0 | 0.150 | 0.953 |
| rpl22 | rpl3 | 1501438 | 1481524 | ENSDARP00000111487 | ENSDARP00000003700 | 0 | 0 | 0 | 0 | 0.815 | 0.958 | 0.900 | 0.587 | 0.999 |
| rbm8a | rpl7 | 1483952 | 1483164 | ENSDARP00000026575 | ENSDARP00000018980 | 0 | 0 | 0 | 0 | 0.099 | 0 | 0.900 | 0 | 0.906 |
| rpl23 | rpl3 | 1489208 | 1481524 | ENSDARP00000069977 | ENSDARP00000003700 | 0.493 | 0 | 0 | 0 | 0.998 | 0.966 | 0.900 | 0.330 | 0.999 |
| ns:zf-e68 | actc1b | 1501354 | 1486630 | ENSDARP00000111326 | ENSDARP00000055135 | 0 | 0 | 0 | 0 | 0.770 | 0.294 | 0 | 0.492 | 0.912 |
| myl2 | desmb | 1503455 | 1488347 | ENSDARP00000116241 | ENSDARP00000065355 | 0 | 0 | 0 | 0 | 0 | 0.071 | 0.900 | 0 | 0.903 |
| eif4a3 | rpl3 | 1484026 | 1481524 | ENSDARP00000027276 | ENSDARP00000003700 | 0 | 0 | 0 | 0 | 0.428 | 0.212 | 0.900 | 0.151 | 0.957 |
| rpl11 | rpl14 | 1488101 | 1486227 | ENSDARP00000063869 | ENSDARP00000052528 | 0.098 | 0 | 0 | 0 | 0.976 | 0.972 | 0.900 | 0.347 | 0.999 |
| rps26 | eef1a1a | 1497897 | 1497414 | ENSDARP00000105328 | ENSDARP00000104468 | 0 | 0 | 0 | 0 | 0.305 | 0 | 0.900 | 0 | 0.927 |
| actn2 | smyhc1 | 1494007 | 1486922 | ENSDARP00000095652 | ENSDARP00000056852 | 0 | 0 | 0 | 0 | 0.156 | 0.268 | 0.900 | 0.367 | 0.955 |
| rpl14 | rpl5a | 1486227 | 1481770 | ENSDARP00000052528 | ENSDARP00000006085 | 0.167 | 0 | 0 | 0 | 0.990 | 0.867 | 0.900 | 0.464 | 0.999 |
| rpl19 | rpl36a | 1498085 | 1490292 | ENSDARP00000105649 | ENSDARP00000075363 | 0 | 0 | 0 | 0 | 0.998 | 0.965 | 0.900 | 0.468 | 0.999 |
| rps26 | rbm8a | 1497897 | 1483952 | ENSDARP00000105328 | ENSDARP00000026575 | 0 | 0 | 0 | 0 | 0.102 | 0 | 0.900 | 0 | 0.906 |
| rps24 | rpl7 | 1493019 | 1483164 | ENSDARP00000091586 | ENSDARP00000018980 | 0 | 0 | 0 | 0 | 0.786 | 0.873 | 0.900 | 0.491 | 0.998 |
| actn2 | calm1a | 1494007 | 1493196 | ENSDARP00000095652 | ENSDARP00000092307 | 0 | 0 | 0 | 0.762 | 0 | 0.079 | 0.900 | 0.121 | 0.905 |
| rps26l | rps16 | 1501604 | 1488590 | ENSDARP00000111782 | ENSDARP00000066897 | 0 | 0 | 0 | 0 | 0.998 | 0.858 | 0.900 | 0.121 | 0.999 |
| rps10 | rpl5a | 1485542 | 1481770 | ENSDARP00000045900 | ENSDARP00000006085 | 0 | 0 | 0 | 0 | 0.937 | 0.184 | 0.900 | 0.788 | 0.998 |
| ckmb | actc1b | 1487353 | 1486630 | ENSDARP00000059365 | ENSDARP00000055135 | 0 | 0 | 0 | 0 | 0.979 | 0.111 | 0 | 0.097 | 0.981 |
| rps3 | rps27.1 | 1488779 | 1484170 | ENSDARP00000067802 | ENSDARP00000029079 | 0 | 0 | 0 | 0 | 0.999 | 0.800 | 0.900 | 0.330 | 0.999 |
| vbp1 | zgc:65894 | 1501624 | 1481340 | ENSDARP00000111810 | ENSDARP00000002175 | 0 | 0 | 0 | 0 | 0 | 0.339 | 0.900 | 0.095 | 0.934 |
| EIF3F | rps3a | 1495369 | 1486104 | ENSDARP00000099664 | ENSDARP00000051762 | 0 | 0 | 0 | 0 | 0.950 | 0 | 0.900 | 0 | 0.994 |
| rps24 | rbm8a | 1493019 | 1483952 | ENSDARP00000091586 | ENSDARP00000026575 | 0 | 0 | 0 | 0 | 0.094 | 0 | 0.900 | 0 | 0.905 |
| rpl23 | rpl13 | 1489208 | 1485678 | ENSDARP00000069977 | ENSDARP00000047390 | 0 | 0 | 0 | 0 | 0.998 | 0.975 | 0.900 | 0.201 | 0.999 |
| rpl7 | eif4eb | 1483164 | 1481876 | ENSDARP00000018980 | ENSDARP00000007117 | 0 | 0 | 0 | 0 | 0.151 | 0 | 0.900 | 0 | 0.911 |
| eno3 | aldoab | 1505202 | 1485195 | ENSDARP00000120742 | ENSDARP00000042199 | 0 | 0 | 0 | 0 | 0.774 | 0.070 | 0.800 | 0.459 | 0.974 |
| aldoab | ckma | 1485195 | 1484819 | ENSDARP00000042199 | ENSDARP00000037871 | 0 | 0 | 0 | 0 | 0.951 | 0.069 | 0 | 0.112 | 0.957 |
| ns:zf-e68 | ckma | 1501354 | 1484819 | ENSDARP00000111326 | ENSDARP00000037871 | 0 | 0 | 0 | 0 | 0.867 | 0.114 | 0 | 0.507 | 0.938 |
| rps10 | rpl7 | 1485542 | 1483164 | ENSDARP00000045900 | ENSDARP00000018980 | 0 | 0 | 0 | 0 | 0.986 | 0.118 | 0.900 | 0.536 | 0.999 |
| rpl6 | rps24 | 1493092 | 1493019 | ENSDARP00000091899 | ENSDARP00000091586 | 0 | 0 | 0 | 0 | 0.882 | 0.831 | 0.900 | 0.163 | 0.998 |
| eif2s1 | rps27.1 | 1488921 | 1484170 | ENSDARP00000068470 | ENSDARP00000029079 | 0.493 | 0 | 0 | 0 | 0.097 | 0.080 | 0.900 | 0 | 0.952 |
| rpsa | eif4eb | 1506145 | 1481876 | ENSDARP00000123183 | ENSDARP00000007117 | 0 | 0 | 0 | 0 | 0.082 | 0 | 0.900 | 0.083 | 0.908 |
| rpl19 | rpl13 | 1498085 | 1485678 | ENSDARP00000105649 | ENSDARP00000047390 | 0 | 0 | 0 | 0 | 0.999 | 0.978 | 0.900 | 0.229 | 0.999 |
| rpsa | rps10 | 1506145 | 1485542 | ENSDARP00000123183 | ENSDARP00000045900 | 0 | 0 | 0 | 0 | 0.943 | 0.970 | 0.900 | 0.308 | 0.999 |
| rpl32 | eif4a3 | 1487450 | 1484026 | ENSDARP00000060004 | ENSDARP00000027276 | 0 | 0 | 0 | 0 | 0.157 | 0 | 0.900 | 0.177 | 0.924 |
| gapdh | eno1a | 1488083 | 1481528 | ENSDARP00000063799 | ENSDARP00000003738 | 0.493 | 0 | 0 | 0 | 0.702 | 0.475 | 0 | 0.613 | 0.966 |
| desma | desmb | 1490431 | 1488347 | ENSDARP00000075994 | ENSDARP00000065355 | 0 | 0 | 0 | 0.979 | 0 | 0 | 0.900 | 0 | 0.900 |
| spcs1 | rps3a | 1490634 | 1486104 | ENSDARP00000076814 | ENSDARP00000051762 | 0 | 0 | 0 | 0 | 0.284 | 0 | 0.900 | 0 | 0.925 |
| rpl32 | rpl18a | 1487450 | 1484877 | ENSDARP00000060004 | ENSDARP00000038658 | 0 | 0 | 0 | 0 | 0.996 | 0.972 | 0.900 | 0.230 | 0.999 |
| eef1a1a | rps29 | 1497414 | 1487533 | ENSDARP00000104468 | ENSDARP00000060443 | 0 | 0 | 0 | 0 | 0.166 | 0.070 | 0.900 | 0.115 | 0.922 |
| rps3 | rpl28 | 1488779 | 1483702 | ENSDARP00000067802 | ENSDARP00000024189 | 0 | 0 | 0 | 0 | 0.999 | 0.120 | 0.900 | 0.189 | 0.999 |
| rpl36a | rps23 | 1490292 | 1484618 | ENSDARP00000075363 | ENSDARP00000035273 | 0 | 0 | 0 | 0 | 0.999 | 0 | 0.900 | 0.178 | 0.999 |
| rpl36a | rps27.2 | 1490292 | 1489628 | ENSDARP00000075363 | ENSDARP00000072300 | 0.493 | 0 | 0 | 0 | 0.996 | 0 | 0.900 | 0.237 | 0.999 |
| rpl11 | eef2l2 | 1488101 | 1486009 | ENSDARP00000063869 | ENSDARP00000051080 | 0.493 | 0 | 0 | 0 | 0.918 | 0.663 | 0 | 0.330 | 0.989 |
| pdlim7 | tpm3 | 1485440 | 1481594 | ENSDARP00000044908 | ENSDARP00000004352 | 0 | 0 | 0 | 0 | 0.953 | 0.114 | 0 | 0.108 | 0.960 |
| rplp2 | rpl5a | 1483852 | 1481770 | ENSDARP00000025616 | ENSDARP00000006085 | 0 | 0 | 0 | 0 | 0.998 | 0.743 | 0 | 0.723 | 0.999 |
| smyhc1 | tpma | 1486922 | 1484960 | ENSDARP00000056852 | ENSDARP00000039656 | 0 | 0 | 0 | 0 | 0.706 | 0.298 | 0 | 0.586 | 0.911 |
| eif2s1 | rpl28 | 1488921 | 1483702 | ENSDARP00000068470 | ENSDARP00000024189 | 0 | 0 | 0 | 0 | 0.067 | 0 | 0.900 | 0 | 0.902 |
| MYL3 | tpm3 | 1488531 | 1481594 | ENSDARP00000066500 | ENSDARP00000004352 | 0 | 0 | 0 | 0 | 0.098 | 0.134 | 0.900 | 0.087 | 0.919 |
| rpl36a | rps3a | 1490292 | 1486104 | ENSDARP00000075363 | ENSDARP00000051762 | 0 | 0 | 0 | 0 | 0.999 | 0.456 | 0.900 | 0.625 | 0.999 |
| rpl19 | rps26 | 1498085 | 1497897 | ENSDARP00000105649 | ENSDARP00000105328 | 0 | 0 | 0 | 0 | 0.988 | 0.309 | 0.900 | 0.365 | 0.999 |
| rpl6 | rpl18a | 1493092 | 1484877 | ENSDARP00000091899 | ENSDARP00000038658 | 0 | 0 | 0 | 0 | 0.997 | 0.972 | 0.900 | 0.245 | 0.999 |
| rps26 | rps29 | 1497897 | 1487533 | ENSDARP00000105328 | ENSDARP00000060443 | 0 | 0 | 0 | 0 | 0.994 | 0.861 | 0.900 | 0.777 | 0.999 |
| cox7c | atp5d | 1489195 | 1483512 | ENSDARP00000069896 | ENSDARP00000022528 | 0 | 0 | 0 | 0 | 0.962 | 0 | 0 | 0.334 | 0.975 |
| rps24 | rpl13 | 1493019 | 1485678 | ENSDARP00000091586 | ENSDARP00000047390 | 0 | 0 | 0 | 0 | 0.859 | 0.458 | 0.900 | 0.231 | 0.993 |
| rpl6 | rpl11 | 1493092 | 1488101 | ENSDARP00000091899 | ENSDARP00000063869 | 0.098 | 0 | 0 | 0 | 0.955 | 0.975 | 0.900 | 0.241 | 0.999 |
| rpl19 | rps29 | 1498085 | 1487533 | ENSDARP00000105649 | ENSDARP00000060443 | 0.493 | 0 | 0 | 0 | 0.970 | 0 | 0.900 | 0.178 | 0.998 |
| spcs1 | rpl36a | 1490634 | 1490292 | ENSDARP00000076814 | ENSDARP00000075363 | 0 | 0 | 0 | 0 | 0.694 | 0 | 0.900 | 0 | 0.968 |
| rpl11 | rbm8a | 1488101 | 1483952 | ENSDARP00000063869 | ENSDARP00000026575 | 0 | 0 | 0 | 0 | 0.095 | 0.415 | 0.900 | 0 | 0.942 |
| myl2 | tnnc1a | 1503455 | 1483843 | ENSDARP00000116241 | ENSDARP00000025541 | 0 | 0 | 0 | 0 | 0.105 | 0.080 | 0.900 | 0.206 | 0.925 |
| rbm8a | u2af1 | 1483952 | 1481720 | ENSDARP00000026575 | ENSDARP00000005582 | 0 | 0 | 0 | 0 | 0.155 | 0 | 0.900 | 0.120 | 0.919 |
| rpl18a | rbm8a | 1484877 | 1483952 | ENSDARP00000038658 | ENSDARP00000026575 | 0 | 0 | 0 | 0 | 0.098 | 0 | 0.900 | 0.067 | 0.908 |
| rps26 | rps27.2 | 1497897 | 1489628 | ENSDARP00000105328 | ENSDARP00000072300 | 0 | 0 | 0 | 0 | 0.996 | 0.678 | 0.900 | 0.380 | 0.999 |
| rps16 | eif4eb | 1488590 | 1481876 | ENSDARP00000066897 | ENSDARP00000007117 | 0 | 0 | 0 | 0 | 0.150 | 0 | 0.900 | 0.080 | 0.914 |
| EIF3F | rpl32 | 1495369 | 1487450 | ENSDARP00000099664 | ENSDARP00000060004 | 0 | 0 | 0 | 0 | 0.278 | 0 | 0.900 | 0 | 0.924 |
| rpl14 | rps27.1 | 1486227 | 1484170 | ENSDARP00000052528 | ENSDARP00000029079 | 0 | 0 | 0 | 0 | 0.996 | 0.450 | 0.900 | 0.152 | 0.999 |
| pdlim7 | tmem38a | 1485440 | 1484755 | ENSDARP00000044908 | ENSDARP00000037150 | 0 | 0 | 0 | 0 | 0.918 | 0 | 0 | 0 | 0.918 |
| EIF3F | rpl35 | 1495369 | 1483123 | ENSDARP00000099664 | ENSDARP00000018594 | 0 | 0 | 0 | 0 | 0.753 | 0 | 0.900 | 0 | 0.974 |
| rps16 | rbm8a | 1488590 | 1483952 | ENSDARP00000066897 | ENSDARP00000026575 | 0 | 0 | 0 | 0 | 0.120 | 0.166 | 0.900 | 0.064 | 0.922 |
| rps27.1 | eif4a3 | 1484170 | 1484026 | ENSDARP00000029079 | ENSDARP00000027276 | 0 | 0 | 0 | 0 | 0.137 | 0 | 0.900 | 0.118 | 0.917 |
| eif2s1 | rpl14 | 1488921 | 1486227 | ENSDARP00000068470 | ENSDARP00000052528 | 0 | 0 | 0 | 0 | 0.184 | 0 | 0.900 | 0 | 0.914 |
| rpl22 | rps3 | 1501438 | 1488779 | ENSDARP00000111487 | ENSDARP00000067802 | 0 | 0 | 0 | 0 | 0.812 | 0.673 | 0.900 | 0.570 | 0.997 |
| rps23 | rpl7 | 1484618 | 1483164 | ENSDARP00000035273 | ENSDARP00000018980 | 0.351 | 0 | 0 | 0 | 0.974 | 0.692 | 0.900 | 0.471 | 0.999 |
| rpl36a | rps10 | 1490292 | 1485542 | ENSDARP00000075363 | ENSDARP00000045900 | 0 | 0 | 0 | 0 | 0.923 | 0 | 0.900 | 0.118 | 0.992 |
| rpl19 | rps27.1 | 1498085 | 1484170 | ENSDARP00000105649 | ENSDARP00000029079 | 0 | 0 | 0 | 0 | 0.973 | 0.450 | 0.900 | 0.330 | 0.998 |
| actc1a | myl10 | 1495696 | 1485924 | ENSDARP00000100434 | ENSDARP00000050204 | 0 | 0 | 0 | 0 | 0.929 | 0.180 | 0 | 0.231 | 0.951 |
| ndufv1 | ndufs4 | 1486296 | 1486007 | ENSDARP00000052929 | ENSDARP00000051054 | 0 | 0 | 0 | 0 | 0.456 | 0 | 0.933 | 0.716 | 0.989 |
| rps26l | rbm8a | 1501604 | 1483952 | ENSDARP00000111782 | ENSDARP00000026575 | 0 | 0 | 0 | 0 | 0.102 | 0 | 0.900 | 0 | 0.906 |
| tnnt3b | atp2a1l | 1497962 | 1494243 | ENSDARP00000105443 | ENSDARP00000096674 | 0 | 0 | 0 | 0 | 0.967 | 0 | 0 | 0.201 | 0.973 |
| rps26 | rpl18a | 1497897 | 1484877 | ENSDARP00000105328 | ENSDARP00000038658 | 0 | 0 | 0 | 0 | 0.998 | 0.363 | 0.900 | 0.120 | 0.999 |
| rpl36a | rplp2 | 1490292 | 1483852 | ENSDARP00000075363 | ENSDARP00000025616 | 0 | 0 | 0 | 0 | 0.999 | 0.782 | 0 | 0.508 | 0.999 |
| ns:zf-e68 | tpma | 1501354 | 1484960 | ENSDARP00000111326 | ENSDARP00000039656 | 0 | 0 | 0 | 0 | 0.846 | 0.298 | 0 | 0.583 | 0.953 |
| rpl19 | rpl5a | 1498085 | 1481770 | ENSDARP00000105649 | ENSDARP00000006085 | 0.493 | 0 | 0 | 0 | 0.997 | 0.874 | 0.900 | 0.358 | 0.999 |
| ndufa12 | ndufv1 | 1487803 | 1486296 | ENSDARP00000062277 | ENSDARP00000052929 | 0 | 0 | 0 | 0 | 0.197 | 0 | 0.933 | 0.177 | 0.953 |
| rps24 | eif2s1 | 1493019 | 1488921 | ENSDARP00000091586 | ENSDARP00000068470 | 0 | 0 | 0 | 0 | 0.118 | 0 | 0.900 | 0 | 0.908 |
| rpl14 | rpl28 | 1486227 | 1483702 | ENSDARP00000052528 | ENSDARP00000024189 | 0 | 0 | 0 | 0 | 0.976 | 0.896 | 0.900 | 0.232 | 0.999 |
| desma | myl1 | 1490431 | 1481643 | ENSDARP00000075994 | ENSDARP00000004932 | 0 | 0 | 0 | 0 | 0.810 | 0.113 | 0.900 | 0 | 0.981 |
| ATP5B | atp5h | 1487508 | 1484892 | ENSDARP00000060309 | ENSDARP00000038799 | 0 | 0 | 0 | 0 | 0.935 | 0.934 | 0.932 | 0.771 | 0.999 |
| rps24 | rps27.1 | 1493019 | 1484170 | ENSDARP00000091586 | ENSDARP00000029079 | 0 | 0 | 0 | 0 | 0.962 | 0.800 | 0.900 | 0.239 | 0.999 |
| rps26l | EIF3F | 1501604 | 1495369 | ENSDARP00000111782 | ENSDARP00000099664 | 0 | 0 | 0 | 0 | 0.799 | 0 | 0.900 | 0 | 0.979 |
| myl2 | tnnc2 | 1503455 | 1493861 | ENSDARP00000116241 | ENSDARP00000095111 | 0 | 0 | 0 | 0 | 0.095 | 0.080 | 0.900 | 0.114 | 0.916 |
| pgk1 | gpib | 1489379 | 1482688 | ENSDARP00000070807 | ENSDARP00000014578 | 0.098 | 0 | 0 | 0 | 0.898 | 0 | 0 | 0.610 | 0.960 |
| rpl23 | rpl28 | 1489208 | 1483702 | ENSDARP00000069977 | ENSDARP00000024189 | 0 | 0 | 0 | 0 | 0.999 | 0.957 | 0.900 | 0.202 | 0.999 |
| rpl32 | rpl28 | 1487450 | 1483702 | ENSDARP00000060004 | ENSDARP00000024189 | 0 | 0 | 0 | 0 | 0.995 | 0.956 | 0.900 | 0.201 | 0.999 |
| aldoab | tpma | 1485195 | 1484960 | ENSDARP00000042199 | ENSDARP00000039656 | 0 | 0 | 0 | 0 | 0.948 | 0.069 | 0 | 0.086 | 0.953 |
| actn2 | tnnc2 | 1494007 | 1493861 | ENSDARP00000095652 | ENSDARP00000095111 | 0 | 0 | 0 | 0 | 0.091 | 0.079 | 0.900 | 0.284 | 0.933 |
| tnnt3b | tpma | 1497962 | 1484960 | ENSDARP00000105443 | ENSDARP00000039656 | 0 | 0 | 0 | 0 | 0.910 | 0.378 | 0 | 0.819 | 0.989 |
| rps26l | rpl32 | 1501604 | 1487450 | ENSDARP00000111782 | ENSDARP00000060004 | 0 | 0 | 0 | 0 | 0.970 | 0.435 | 0.900 | 0.118 | 0.998 |
| aldoaa | gapdhs | 1504686 | 1487169 | ENSDARP00000119413 | ENSDARP00000058383 | 0.257 | 0 | 0 | 0 | 0.489 | 0.206 | 0.949 | 0.439 | 0.990 |
| eef1a1a | rps3 | 1497414 | 1488779 | ENSDARP00000104468 | ENSDARP00000067802 | 0 | 0 | 0 | 0 | 0.697 | 0.174 | 0.900 | 0.119 | 0.974 |
| rps26l | eif2s1 | 1501604 | 1488921 | ENSDARP00000111782 | ENSDARP00000068470 | 0 | 0 | 0 | 0 | 0.135 | 0 | 0.900 | 0 | 0.909 |
| rpl23 | eif4a3 | 1489208 | 1484026 | ENSDARP00000069977 | ENSDARP00000027276 | 0 | 0 | 0 | 0 | 0.263 | 0.122 | 0.900 | 0.083 | 0.934 |
| aldoaa | eno2 | 1504686 | 1484405 | ENSDARP00000119413 | ENSDARP00000032456 | 0 | 0 | 0 | 0 | 0.750 | 0.070 | 0.800 | 0.459 | 0.971 |
| atp2a1l | slc25a4 | 1494243 | 1484291 | ENSDARP00000096674 | ENSDARP00000030881 | 0 | 0 | 0 | 0 | 0.963 | 0.277 | 0 | 0.070 | 0.972 |
| pabpc4 | eif4eb | 1490616 | 1481876 | ENSDARP00000076763 | ENSDARP00000007117 | 0 | 0 | 0 | 0 | 0 | 0.485 | 0.511 | 0.718 | 0.922 |
| rps10 | rpl28 | 1485542 | 1483702 | ENSDARP00000045900 | ENSDARP00000024189 | 0 | 0 | 0 | 0 | 0.993 | 0.139 | 0.900 | 0.606 | 0.999 |
| desma | mylz3 | 1490431 | 1483092 | ENSDARP00000075994 | ENSDARP00000018197 | 0 | 0 | 0 | 0 | 0.621 | 0.113 | 0.900 | 0.375 | 0.976 |
| eef1a1a | rps10 | 1497414 | 1485542 | ENSDARP00000104468 | ENSDARP00000045900 | 0 | 0 | 0 | 0 | 0.278 | 0.081 | 0.900 | 0.064 | 0.929 |
| rps26 | rps10 | 1497897 | 1485542 | ENSDARP00000105328 | ENSDARP00000045900 | 0 | 0 | 0 | 0 | 0.940 | 0.839 | 0.900 | 0.917 | 0.999 |
| rps16 | eif4a3 | 1488590 | 1484026 | ENSDARP00000066897 | ENSDARP00000027276 | 0 | 0 | 0 | 0 | 0.285 | 0.120 | 0.900 | 0.087 | 0.936 |
| eef1a1a | rpl28 | 1497414 | 1483702 | ENSDARP00000104468 | ENSDARP00000024189 | 0 | 0 | 0 | 0 | 0.169 | 0 | 0.900 | 0 | 0.913 |
| eef1a1a | rpl18a | 1497414 | 1484877 | ENSDARP00000104468 | ENSDARP00000038658 | 0 | 0 | 0 | 0 | 0.492 | 0.281 | 0.900 | 0.096 | 0.962 |
| tnnc2 | pdlim7 | 1493861 | 1485440 | ENSDARP00000095111 | ENSDARP00000044908 | 0 | 0 | 0 | 0 | 0.983 | 0.083 | 0 | 0.326 | 0.989 |
| desma | tpm3 | 1490431 | 1481594 | ENSDARP00000075994 | ENSDARP00000004352 | 0 | 0 | 0 | 0 | 0.830 | 0 | 0.900 | 0.358 | 0.988 |
| rpsa | eif2s1 | 1506145 | 1488921 | ENSDARP00000123183 | ENSDARP00000068470 | 0 | 0 | 0 | 0 | 0.166 | 0 | 0.900 | 0 | 0.913 |
| rps27.1 | rpl5a | 1484170 | 1481770 | ENSDARP00000029079 | ENSDARP00000006085 | 0 | 0 | 0 | 0 | 0.985 | 0.345 | 0.900 | 0.359 | 0.999 |
| LOC567740 | desmb | 1505894 | 1488347 | ENSDARP00000122502 | ENSDARP00000065355 | 0 | 0 | 0 | 0 | 0 | 0 | 0.900 | 0.064 | 0.902 |
| rpsa | EIF3F | 1506145 | 1495369 | ENSDARP00000123183 | ENSDARP00000099664 | 0 | 0 | 0 | 0 | 0.697 | 0.070 | 0.900 | 0 | 0.970 |
| rpl13 | rpl3 | 1485678 | 1481524 | ENSDARP00000047390 | ENSDARP00000003700 | 0 | 0 | 0 | 0 | 0.999 | 0.962 | 0.900 | 0.819 | 0.999 |
| rpl11 | eif4a3 | 1488101 | 1484026 | ENSDARP00000063869 | ENSDARP00000027276 | 0 | 0 | 0 | 0 | 0.261 | 0.136 | 0.900 | 0.225 | 0.945 |
| ddost | rpl18a | 1486503 | 1484877 | ENSDARP00000054289 | ENSDARP00000038658 | 0 | 0 | 0 | 0 | 0.097 | 0 | 0.900 | 0 | 0.905 |
| atp5a1 | atp5d | 1484089 | 1483512 | ENSDARP00000027947 | ENSDARP00000022528 | 0.493 | 0 | 0 | 0 | 0.768 | 0.941 | 0.937 | 0.877 | 0.999 |
| rpl36a | eif4a3 | 1490292 | 1484026 | ENSDARP00000075363 | ENSDARP00000027276 | 0 | 0 | 0 | 0 | 0.099 | 0 | 0.900 | 0 | 0.906 |
| ddost | rpl28 | 1486503 | 1483702 | ENSDARP00000054289 | ENSDARP00000024189 | 0 | 0 | 0 | 0 | 0.097 | 0 | 0.900 | 0 | 0.905 |
| rps16 | rps27.1 | 1488590 | 1484170 | ENSDARP00000066897 | ENSDARP00000029079 | 0 | 0 | 0 | 0 | 0.998 | 0.800 | 0.900 | 0.217 | 0.999 |
| rps3 | rpl18a | 1488779 | 1484877 | ENSDARP00000067802 | ENSDARP00000038658 | 0 | 0 | 0 | 0 | 0.999 | 0.875 | 0.900 | 0.176 | 0.999 |
| tnnt3b | pvalb1 | 1497962 | 1486619 | ENSDARP00000105443 | ENSDARP00000055061 | 0 | 0 | 0 | 0 | 0.983 | 0.255 | 0 | 0.574 | 0.994 |
| calm1a | tpm3 | 1493196 | 1481594 | ENSDARP00000092307 | ENSDARP00000004352 | 0 | 0 | 0 | 0 | 0 | 0.127 | 0.900 | 0.159 | 0.920 |
| tnnc1a | mylz3 | 1483843 | 1483092 | ENSDARP00000025541 | ENSDARP00000018197 | 0 | 0 | 0 | 0.789 | 0 | 0 | 0.900 | 0 | 0.900 |
| ckmb | slc25a4 | 1487353 | 1484291 | ENSDARP00000059365 | ENSDARP00000030881 | 0 | 0 | 0 | 0 | 0.903 | 0.080 | 0.191 | 0.064 | 0.923 |
| LOC567740 | MYL3 | 1505894 | 1488531 | ENSDARP00000122502 | ENSDARP00000066500 | 0 | 0 | 0 | 0 | 0 | 0.202 | 0.900 | 0.209 | 0.931 |
| smyhc1 | tpm3 | 1486922 | 1481594 | ENSDARP00000056852 | ENSDARP00000004352 | 0 | 0 | 0 | 0 | 0.921 | 0.298 | 0.900 | 0.673 | 0.998 |
| rpl11 | ddost | 1488101 | 1486503 | ENSDARP00000063869 | ENSDARP00000054289 | 0 | 0 | 0 | 0 | 0.106 | 0 | 0.900 | 0 | 0.906 |
| rps26 | rpl23 | 1497897 | 1489208 | ENSDARP00000105328 | ENSDARP00000069977 | 0 | 0 | 0 | 0 | 0.998 | 0.405 | 0.900 | 0.087 | 0.999 |
| NDUFC2 | ndufv1 | 1500789 | 1486296 | ENSDARP00000110392 | ENSDARP00000052929 | 0 | 0 | 0 | 0 | 0.123 | 0 | 0.933 | 0.118 | 0.946 |
| eif4a3 | rpl7 | 1484026 | 1483164 | ENSDARP00000027276 | ENSDARP00000018980 | 0 | 0 | 0 | 0 | 0.192 | 0.291 | 0.900 | 0.151 | 0.944 |
| rps26 | rplp2 | 1497897 | 1483852 | ENSDARP00000105328 | ENSDARP00000025616 | 0 | 0 | 0 | 0 | 0.986 | 0.341 | 0 | 0.096 | 0.991 |
| MYL3 | desmb | 1488531 | 1488347 | ENSDARP00000066500 | ENSDARP00000065355 | 0 | 0 | 0 | 0 | 0 | 0.113 | 0.900 | 0 | 0.907 |
| rpl23 | rps3a | 1489208 | 1486104 | ENSDARP00000069977 | ENSDARP00000051762 | 0 | 0 | 0 | 0 | 0.999 | 0.700 | 0.900 | 0.233 | 0.999 |
| tnnc2 | ckma | 1493861 | 1484819 | ENSDARP00000095111 | ENSDARP00000037871 | 0 | 0 | 0 | 0 | 0.983 | 0.079 | 0 | 0.371 | 0.989 |
| mylz3 | tpm3 | 1483092 | 1481594 | ENSDARP00000018197 | ENSDARP00000004352 | 0 | 0 | 0 | 0 | 0.696 | 0.134 | 0.900 | 0.315 | 0.980 |
| rps26l | rps27.1 | 1501604 | 1484170 | ENSDARP00000111782 | ENSDARP00000029079 | 0 | 0 | 0 | 0 | 0.998 | 0.678 | 0.900 | 0.380 | 0.999 |
| tpi1a | eno2 | 1484521 | 1484405 | ENSDARP00000033907 | ENSDARP00000032456 | 0.493 | 0 | 0 | 0 | 0.941 | 0.286 | 0 | 0.513 | 0.988 |
| rps26 | rpl6 | 1497897 | 1493092 | ENSDARP00000105328 | ENSDARP00000091899 | 0 | 0 | 0 | 0 | 0.981 | 0.202 | 0.900 | 0.146 | 0.998 |
| rpl11 | rps27.1 | 1488101 | 1484170 | ENSDARP00000063869 | ENSDARP00000029079 | 0 | 0 | 0 | 0 | 0.964 | 0.450 | 0.900 | 0.358 | 0.998 |
| rps26l | rpl22 | 1501604 | 1501438 | ENSDARP00000111782 | ENSDARP00000111487 | 0 | 0 | 0 | 0 | 0.961 | 0.382 | 0.900 | 0.489 | 0.998 |
| dnaja2 | hsp90aa1.1 | 1484139 | 1483481 | ENSDARP00000028641 | ENSDARP00000022302 | 0 | 0 | 0 | 0 | 0.690 | 0.476 | 0 | 0.768 | 0.960 |
| rps24 | rps16 | 1493019 | 1488590 | ENSDARP00000091586 | ENSDARP00000066897 | 0 | 0 | 0 | 0 | 0.904 | 0.975 | 0.900 | 0.177 | 0.999 |
| aldoaa | gapdh | 1504686 | 1488083 | ENSDARP00000119413 | ENSDARP00000063799 | 0.257 | 0 | 0 | 0 | 0.278 | 0.206 | 0.949 | 0.439 | 0.986 |
| rpl13 | rpl5a | 1485678 | 1481770 | ENSDARP00000047390 | ENSDARP00000006085 | 0 | 0 | 0 | 0 | 0.997 | 0.875 | 0.900 | 0.509 | 0.999 |
| tnni2a.4 | tpm3 | 1484806 | 1481594 | ENSDARP00000037759 | ENSDARP00000004352 | 0 | 0 | 0 | 0 | 0.293 | 0.285 | 0.900 | 0.733 | 0.985 |
| rps26 | ddost | 1497897 | 1486503 | ENSDARP00000105328 | ENSDARP00000054289 | 0 | 0 | 0 | 0 | 0.091 | 0 | 0.900 | 0 | 0.905 |
| rps10 | rpl35 | 1485542 | 1483123 | ENSDARP00000045900 | ENSDARP00000018594 | 0 | 0 | 0 | 0 | 0.939 | 0 | 0.900 | 0.184 | 0.994 |
| rpsa | rbm8a | 1506145 | 1483952 | ENSDARP00000123183 | ENSDARP00000026575 | 0 | 0 | 0 | 0 | 0.099 | 0.125 | 0.900 | 0.097 | 0.919 |
| spcs1 | rpl28 | 1490634 | 1483702 | ENSDARP00000076814 | ENSDARP00000024189 | 0 | 0 | 0 | 0 | 0.329 | 0 | 0.900 | 0 | 0.930 |
| aldoaa | tpi1a | 1504686 | 1484521 | ENSDARP00000119413 | ENSDARP00000033907 | 0 | 0 | 0 | 0 | 0.508 | 0 | 0.952 | 0.691 | 0.992 |
| EIF3F | rpl36a | 1495369 | 1490292 | ENSDARP00000099664 | ENSDARP00000075363 | 0 | 0 | 0 | 0 | 0.810 | 0 | 0.900 | 0 | 0.980 |
| desma | smyhc1 | 1490431 | 1486922 | ENSDARP00000075994 | ENSDARP00000056852 | 0 | 0 | 0 | 0 | 0.536 | 0 | 0.900 | 0.064 | 0.952 |
| LOC567740 | tpm3 | 1505894 | 1481594 | ENSDARP00000122502 | ENSDARP00000004352 | 0 | 0 | 0 | 0 | 0.131 | 0.298 | 0.900 | 0.380 | 0.957 |
| eef1a1a | rpl3 | 1497414 | 1481524 | ENSDARP00000104468 | ENSDARP00000003700 | 0 | 0 | 0 | 0 | 0.705 | 0 | 0.900 | 0.362 | 0.979 |
| atp2a1l | actc1b | 1494243 | 1486630 | ENSDARP00000096674 | ENSDARP00000055135 | 0 | 0 | 0 | 0 | 0.901 | 0.072 | 0 | 0.201 | 0.920 |
| pgk1 | aldocb | 1489379 | 1483738 | ENSDARP00000070807 | ENSDARP00000024492 | 0.378 | 0 | 0 | 0 | 0.617 | 0.177 | 0.408 | 0.503 | 0.933 |
| eno3 | aldocb | 1505202 | 1483738 | ENSDARP00000120742 | ENSDARP00000024492 | 0 | 0 | 0 | 0 | 0.750 | 0.070 | 0.800 | 0.459 | 0.971 |
| rps16 | rpl11 | 1488590 | 1488101 | ENSDARP00000066897 | ENSDARP00000063869 | 0.246 | 0 | 0 | 0 | 0.998 | 0.874 | 0.900 | 0.330 | 0.999 |
| actc1b | tpma | 1486630 | 1484960 | ENSDARP00000055135 | ENSDARP00000039656 | 0 | 0 | 0 | 0 | 0.982 | 0.360 | 0 | 0.453 | 0.993 |
| rpl14 | eif4a3 | 1486227 | 1484026 | ENSDARP00000052528 | ENSDARP00000027276 | 0 | 0 | 0 | 0 | 0.121 | 0.160 | 0.900 | 0 | 0.919 |
| gapdh | ckma | 1488083 | 1484819 | ENSDARP00000063799 | ENSDARP00000037871 | 0 | 0 | 0 | 0 | 0.903 | 0.210 | 0 | 0.248 | 0.940 |
| rpl14 | rbm8a | 1486227 | 1483952 | ENSDARP00000052528 | ENSDARP00000026575 | 0 | 0 | 0 | 0 | 0.118 | 0 | 0.900 | 0 | 0.908 |
| tnni2a.4 | mylz3 | 1484806 | 1483092 | ENSDARP00000037759 | ENSDARP00000018197 | 0 | 0 | 0 | 0 | 0.692 | 0.187 | 0.900 | 0.449 | 0.985 |
| rpl32 | rpl5a | 1487450 | 1481770 | ENSDARP00000060004 | ENSDARP00000006085 | 0.493 | 0 | 0 | 0 | 0.996 | 0.873 | 0.900 | 0.330 | 0.999 |
| tnnt3b | ckma | 1497962 | 1484819 | ENSDARP00000105443 | ENSDARP00000037871 | 0 | 0 | 0 | 0 | 0.969 | 0.135 | 0 | 0.260 | 0.979 |
| rpl36a | rpl14 | 1490292 | 1486227 | ENSDARP00000075363 | ENSDARP00000052528 | 0 | 0 | 0 | 0 | 0.999 | 0.940 | 0.900 | 0.201 | 0.999 |
| rps16 | rps29 | 1488590 | 1487533 | ENSDARP00000066897 | ENSDARP00000060443 | 0.241 | 0 | 0 | 0 | 0.998 | 0.978 | 0.900 | 0.330 | 0.999 |
| rps26l | rps24 | 1501604 | 1493019 | ENSDARP00000111782 | ENSDARP00000091586 | 0 | 0 | 0 | 0 | 0.824 | 0.861 | 0.900 | 0.373 | 0.998 |
| rps26 | eif4eb | 1497897 | 1481876 | ENSDARP00000105328 | ENSDARP00000007117 | 0 | 0 | 0 | 0 | 0.111 | 0 | 0.900 | 0.232 | 0.925 |
| rpl28 | rpl3 | 1483702 | 1481524 | ENSDARP00000024189 | ENSDARP00000003700 | 0 | 0 | 0 | 0 | 0.967 | 0.916 | 0.900 | 0.183 | 0.999 |
| spcs1 | rpl23 | 1490634 | 1489208 | ENSDARP00000076814 | ENSDARP00000069977 | 0 | 0 | 0 | 0 | 0.179 | 0.152 | 0.900 | 0 | 0.924 |
| rpl11 | rpl3 | 1488101 | 1481524 | ENSDARP00000063869 | ENSDARP00000003700 | 0.493 | 0 | 0 | 0 | 0.999 | 0.966 | 0.900 | 0.524 | 0.999 |
| gpib | eno1a | 1482688 | 1481528 | ENSDARP00000014578 | ENSDARP00000003738 | 0.098 | 0 | 0 | 0 | 0.742 | 0.132 | 0.800 | 0.612 | 0.981 |
| tnnc2 | aldoab | 1493861 | 1485195 | ENSDARP00000095111 | ENSDARP00000042199 | 0 | 0 | 0 | 0 | 0.980 | 0.072 | 0 | 0.182 | 0.983 |
| rpl14 | rpl7 | 1486227 | 1483164 | ENSDARP00000052528 | ENSDARP00000018980 | 0.190 | 0 | 0 | 0 | 0.967 | 0.972 | 0.900 | 0.369 | 0.999 |
| rps24 | rps27.2 | 1493019 | 1489628 | ENSDARP00000091586 | ENSDARP00000072300 | 0 | 0 | 0 | 0 | 0.897 | 0.800 | 0.900 | 0.239 | 0.998 |
| desma | tnni2a.4 | 1490431 | 1484806 | ENSDARP00000075994 | ENSDARP00000037759 | 0 | 0 | 0 | 0 | 0.143 | 0 | 0.900 | 0.064 | 0.912 |
| rpl19 | eif4eb | 1498085 | 1481876 | ENSDARP00000105649 | ENSDARP00000007117 | 0 | 0 | 0 | 0 | 0.118 | 0 | 0.900 | 0 | 0.908 |
| EIF3F | rpl3 | 1495369 | 1481524 | ENSDARP00000099664 | ENSDARP00000003700 | 0 | 0 | 0 | 0 | 0.746 | 0 | 0.900 | 0 | 0.974 |
| atp5c1 | atp5a1 | 1488594 | 1484089 | ENSDARP00000066929 | ENSDARP00000027947 | 0.493 | 0 | 0.510 | 0 | 0.980 | 0.978 | 0.937 | 0.795 | 0.999 |
| rpl23 | rpl7 | 1489208 | 1483164 | ENSDARP00000069977 | ENSDARP00000018980 | 0.493 | 0 | 0 | 0 | 0.999 | 0.978 | 0.900 | 0.330 | 0.999 |
| rps29 | rps27.1 | 1487533 | 1484170 | ENSDARP00000060443 | ENSDARP00000029079 | 0 | 0 | 0 | 0 | 0.999 | 0.797 | 0.900 | 0.596 | 0.999 |
| actn2 | tpm3 | 1494007 | 1481594 | ENSDARP00000095652 | ENSDARP00000004352 | 0 | 0 | 0 | 0 | 0.280 | 0.269 | 0.900 | 0.361 | 0.961 |
| rpl22 | rps10 | 1501438 | 1485542 | ENSDARP00000111487 | ENSDARP00000045900 | 0 | 0 | 0 | 0 | 0.635 | 0 | 0.900 | 0.430 | 0.978 |
| eif2s1 | rpl7 | 1488921 | 1483164 | ENSDARP00000068470 | ENSDARP00000018980 | 0 | 0 | 0 | 0 | 0.168 | 0 | 0.900 | 0 | 0.913 |
| rpsa | eef1a1a | 1506145 | 1497414 | ENSDARP00000123183 | ENSDARP00000104468 | 0 | 0 | 0 | 0 | 0.478 | 0.157 | 0.900 | 0.067 | 0.953 |
| rpl7 | rpl3 | 1483164 | 1481524 | ENSDARP00000018980 | ENSDARP00000003700 | 0.493 | 0 | 0 | 0 | 0.994 | 0.966 | 0.900 | 0.767 | 0.999 |
| rpl11 | rps3a | 1488101 | 1486104 | ENSDARP00000063869 | ENSDARP00000051762 | 0 | 0 | 0 | 0 | 0.996 | 0.875 | 0.900 | 0.500 | 0.999 |
| rpl32 | rps27.1 | 1487450 | 1484170 | ENSDARP00000060004 | ENSDARP00000029079 | 0 | 0 | 0 | 0 | 0.996 | 0 | 0.900 | 0.330 | 0.999 |
| spcs1 | rpl35 | 1490634 | 1483123 | ENSDARP00000076814 | ENSDARP00000018594 | 0 | 0 | 0 | 0 | 0.394 | 0.175 | 0.900 | 0 | 0.945 |
| ddost | rps27.1 | 1486503 | 1484170 | ENSDARP00000054289 | ENSDARP00000029079 | 0 | 0 | 0 | 0 | 0 | 0 | 0.900 | 0 | 0.900 |
| eif2s1 | rpl11 | 1488921 | 1488101 | ENSDARP00000068470 | ENSDARP00000063869 | 0 | 0 | 0 | 0 | 0.169 | 0 | 0.900 | 0.238 | 0.931 |
| aldoab | gpib | 1485195 | 1482688 | ENSDARP00000042199 | ENSDARP00000014578 | 0 | 0 | 0 | 0 | 0.711 | 0 | 0.800 | 0.460 | 0.966 |
| rpl6 | rplp2 | 1493092 | 1483852 | ENSDARP00000091899 | ENSDARP00000025616 | 0 | 0 | 0 | 0 | 0.995 | 0.797 | 0 | 0.647 | 0.999 |
| rpsa | rpl11 | 1506145 | 1488101 | ENSDARP00000123183 | ENSDARP00000063869 | 0.080 | 0 | 0 | 0 | 0.997 | 0.278 | 0.900 | 0.330 | 0.999 |
| atp5h | atp5a1 | 1484892 | 1484089 | ENSDARP00000038799 | ENSDARP00000027947 | 0 | 0 | 0 | 0 | 0.491 | 0.939 | 0.932 | 0.607 | 0.999 |
| rpl6 | eif2s1 | 1493092 | 1488921 | ENSDARP00000091899 | ENSDARP00000068470 | 0 | 0 | 0 | 0 | 0.197 | 0 | 0.900 | 0 | 0.916 |
| spcs1 | rpl32 | 1490634 | 1487450 | ENSDARP00000076814 | ENSDARP00000060004 | 0 | 0 | 0 | 0 | 0.094 | 0 | 0.900 | 0 | 0.905 |
| eif2s1 | rpl18a | 1488921 | 1484877 | ENSDARP00000068470 | ENSDARP00000038658 | 0 | 0 | 0 | 0 | 0.178 | 0 | 0.900 | 0 | 0.914 |
| rps3 | rpl7 | 1488779 | 1483164 | ENSDARP00000067802 | ENSDARP00000018980 | 0.493 | 0 | 0 | 0 | 0.998 | 0.876 | 0.900 | 0.358 | 0.999 |
| eef1a1a | rpl23 | 1497414 | 1489208 | ENSDARP00000104468 | ENSDARP00000069977 | 0 | 0 | 0.318 | 0 | 0.427 | 0 | 0.900 | 0.064 | 0.958 |
| rplp2 | rpl28 | 1483852 | 1483702 | ENSDARP00000025616 | ENSDARP00000024189 | 0 | 0 | 0 | 0 | 0.839 | 0.633 | 0 | 0.787 | 0.986 |
| rps3 | rbm8a | 1488779 | 1483952 | ENSDARP00000067802 | ENSDARP00000026575 | 0 | 0 | 0 | 0 | 0.094 | 0 | 0.900 | 0 | 0.905 |
| tnnc2 | actc1b | 1493861 | 1486630 | ENSDARP00000095111 | ENSDARP00000055135 | 0 | 0 | 0 | 0 | 0.983 | 0.245 | 0 | 0.493 | 0.992 |
| rpl11 | rps23 | 1488101 | 1484618 | ENSDARP00000063869 | ENSDARP00000035273 | 0.493 | 0 | 0 | 0 | 0.998 | 0.637 | 0.900 | 0.330 | 0.999 |
| fh | sdhb | 1494425 | 1487800 | ENSDARP00000097494 | ENSDARP00000062263 | 0.140 | 0 | 0 | 0 | 0.452 | 0 | 0.957 | 0.503 | 0.989 |
| tnni2a.1 | tnnc1a | 1484340 | 1483843 | ENSDARP00000031650 | ENSDARP00000025541 | 0 | 0 | 0 | 0 | 0.082 | 0.477 | 0.900 | 0.530 | 0.974 |
| rps27.2 | rpl35 | 1489628 | 1483123 | ENSDARP00000072300 | ENSDARP00000018594 | 0 | 0 | 0 | 0 | 0.988 | 0.079 | 0.900 | 0.064 | 0.998 |
| rpl18a | eif4eb | 1484877 | 1481876 | ENSDARP00000038658 | ENSDARP00000007117 | 0 | 0 | 0 | 0 | 0.222 | 0 | 0.900 | 0 | 0.918 |
| aldoab | tpi1a | 1485195 | 1484521 | ENSDARP00000042199 | ENSDARP00000033907 | 0 | 0 | 0 | 0 | 0.508 | 0 | 0.952 | 0.611 | 0.990 |
| rpsa | rpl22 | 1506145 | 1501438 | ENSDARP00000123183 | ENSDARP00000111487 | 0 | 0 | 0 | 0 | 0.910 | 0.684 | 0.900 | 0.076 | 0.997 |
| rpl6 | rps3 | 1493092 | 1488779 | ENSDARP00000091899 | ENSDARP00000067802 | 0.083 | 0 | 0 | 0 | 0.998 | 0.827 | 0.900 | 0.532 | 0.999 |
| rpl23 | rps29 | 1489208 | 1487533 | ENSDARP00000069977 | ENSDARP00000060443 | 0.493 | 0 | 0 | 0 | 0.998 | 0.119 | 0.900 | 0.330 | 0.999 |
| psma3 | psma2 | 1499751 | 1487227 | ENSDARP00000108560 | ENSDARP00000058679 | 0 | 0 | 0.240 | 0.837 | 0.768 | 0.978 | 0.964 | 0.792 | 0.999 |
| ddost | rpl5a | 1486503 | 1481770 | ENSDARP00000054289 | ENSDARP00000006085 | 0 | 0 | 0 | 0 | 0.088 | 0 | 0.900 | 0 | 0.904 |
| rpl19 | rpl18a | 1498085 | 1484877 | ENSDARP00000105649 | ENSDARP00000038658 | 0 | 0 | 0 | 0 | 0.999 | 0.973 | 0.900 | 0.363 | 0.999 |
| rpl32 | rpl14 | 1487450 | 1486227 | ENSDARP00000060004 | ENSDARP00000052528 | 0.103 | 0 | 0 | 0 | 0.996 | 0.973 | 0.900 | 0.361 | 0.999 |
| rpl6 | rpl3 | 1493092 | 1481524 | ENSDARP00000091899 | ENSDARP00000003700 | 0.071 | 0 | 0 | 0 | 0.982 | 0.963 | 0.900 | 0.350 | 0.999 |
| rpl22 | eif4a3 | 1501438 | 1484026 | ENSDARP00000111487 | ENSDARP00000027276 | 0 | 0 | 0 | 0 | 0.094 | 0 | 0.900 | 0.065 | 0.907 |
| eef1a1l1 | rpl13 | 1501580 | 1485678 | ENSDARP00000111742 | ENSDARP00000047390 | 0 | 0 | 0 | 0 | 0.916 | 0 | 0 | 0 | 0.918 |
| rpsa | rpl32 | 1506145 | 1487450 | ENSDARP00000123183 | ENSDARP00000060004 | 0 | 0 | 0 | 0 | 0.996 | 0.602 | 0.900 | 0.330 | 0.999 |
| MYL3 | smyhc1 | 1488531 | 1486922 | ENSDARP00000066500 | ENSDARP00000056852 | 0 | 0 | 0 | 0 | 0 | 0.202 | 0.900 | 0.209 | 0.931 |
| ndufs4 | atp5d | 1486007 | 1483512 | ENSDARP00000051054 | ENSDARP00000022528 | 0 | 0 | 0 | 0 | 0.930 | 0 | 0 | 0.088 | 0.935 |
| tnnt3b | smyhc1 | 1497962 | 1486922 | ENSDARP00000105443 | ENSDARP00000056852 | 0 | 0 | 0 | 0 | 0.348 | 0.335 | 0.900 | 0.633 | 0.982 |
| pvalb1 | tnni2a.4 | 1486619 | 1484806 | ENSDARP00000055061 | ENSDARP00000037759 | 0 | 0 | 0 | 0 | 0.898 | 0.284 | 0 | 0.586 | 0.968 |
| eef1a1a | vcp | 1497414 | 1482419 | ENSDARP00000104468 | ENSDARP00000012048 | 0 | 0 | 0 | 0 | 0 | 0.070 | 0.900 | 0.243 | 0.923 |
| tnnt3b | ckmb | 1497962 | 1487353 | ENSDARP00000105443 | ENSDARP00000059365 | 0 | 0 | 0 | 0 | 0.967 | 0.135 | 0 | 0 | 0.971 |
| rpl36a | rpl13 | 1490292 | 1485678 | ENSDARP00000075363 | ENSDARP00000047390 | 0 | 0 | 0 | 0 | 0.997 | 0.962 | 0.900 | 0.507 | 0.999 |
| rps3 | rps3a | 1488779 | 1486104 | ENSDARP00000067802 | ENSDARP00000051762 | 0 | 0 | 0 | 0 | 0.999 | 0.977 | 0.900 | 0.699 | 0.999 |
| EIF3F | rpl23 | 1495369 | 1489208 | ENSDARP00000099664 | ENSDARP00000069977 | 0 | 0 | 0 | 0 | 0.748 | 0 | 0.900 | 0 | 0.974 |
| rps26l | rps3 | 1501604 | 1488779 | ENSDARP00000111782 | ENSDARP00000067802 | 0 | 0 | 0 | 0 | 0.993 | 0.861 | 0.900 | 0.527 | 0.999 |
| uqcrc1 | atp5d | 1499876 | 1483512 | ENSDARP00000108798 | ENSDARP00000022528 | 0 | 0 | 0 | 0 | 0.802 | 0.294 | 0 | 0.525 | 0.931 |
| smyhc1 | tnnc1b | 1486922 | 1486560 | ENSDARP00000056852 | ENSDARP00000054663 | 0 | 0 | 0 | 0 | 0 | 0.200 | 0 | 0.902 | 0.920 |
| rps27.2 | rps3 | 1489628 | 1488779 | ENSDARP00000072300 | ENSDARP00000067802 | 0 | 0 | 0 | 0 | 0.996 | 0.800 | 0.900 | 0.330 | 0.999 |
| rpl13 | rpl18a | 1485678 | 1484877 | ENSDARP00000047390 | ENSDARP00000038658 | 0 | 0 | 0 | 0 | 0.999 | 0.957 | 0.900 | 0.209 | 0.999 |
| rpl32 | eif4eb | 1487450 | 1481876 | ENSDARP00000060004 | ENSDARP00000007117 | 0 | 0 | 0 | 0 | 0.164 | 0 | 0.900 | 0.071 | 0.915 |
| desmb | smyhc1 | 1488347 | 1486922 | ENSDARP00000065355 | ENSDARP00000056852 | 0 | 0 | 0 | 0 | 0 | 0 | 0.900 | 0.064 | 0.902 |
| tnnc2 | tpm3 | 1493861 | 1481594 | ENSDARP00000095111 | ENSDARP00000004352 | 0 | 0 | 0 | 0 | 0.925 | 0.127 | 0.900 | 0.694 | 0.997 |
| rpl36a | rps27.1 | 1490292 | 1484170 | ENSDARP00000075363 | ENSDARP00000029079 | 0.493 | 0 | 0 | 0 | 0.999 | 0 | 0.900 | 0.237 | 0.999 |
| rplp2 | rpl3 | 1483852 | 1481524 | ENSDARP00000025616 | ENSDARP00000003700 | 0 | 0 | 0 | 0 | 0.907 | 0.784 | 0 | 0.145 | 0.981 |
| ckmb | pdlim7 | 1487353 | 1485440 | ENSDARP00000059365 | ENSDARP00000044908 | 0 | 0 | 0 | 0 | 0.983 | 0 | 0 | 0 | 0.983 |
| myl2 | MYL3 | 1503455 | 1488531 | ENSDARP00000116241 | ENSDARP00000066500 | 0 | 0 | 0 | 0.711 | 0.116 | 0.072 | 0.900 | 0.664 | 0.927 |
| psma2 | pomp | 1487227 | 1485465 | ENSDARP00000058679 | ENSDARP00000045123 | 0 | 0 | 0 | 0 | 0.953 | 0.839 | 0 | 0.172 | 0.993 |
| rpl19 | rpl35 | 1498085 | 1483123 | ENSDARP00000105649 | ENSDARP00000018594 | 0.493 | 0 | 0 | 0 | 0.988 | 0.965 | 0.900 | 0.150 | 0.999 |
| rps3a | rpl3 | 1486104 | 1481524 | ENSDARP00000051762 | ENSDARP00000003700 | 0 | 0 | 0 | 0 | 0.999 | 0.860 | 0.900 | 0.219 | 0.999 |
| rps29 | rpl7 | 1487533 | 1483164 | ENSDARP00000060443 | ENSDARP00000018980 | 0.493 | 0 | 0 | 0 | 0.954 | 0.457 | 0.900 | 0.731 | 0.999 |
| pkmb | pgk1 | 1505987 | 1489379 | ENSDARP00000122764 | ENSDARP00000070807 | 0.493 | 0 | 0 | 0 | 0.992 | 0 | 0 | 0.605 | 0.998 |
| actc1a | tnnc2 | 1495696 | 1493861 | ENSDARP00000100434 | ENSDARP00000095111 | 0 | 0 | 0 | 0 | 0.919 | 0.245 | 0 | 0.493 | 0.966 |
| eef1a1a | rpl7 | 1497414 | 1483164 | ENSDARP00000104468 | ENSDARP00000018980 | 0 | 0 | 0 | 0 | 0.304 | 0.113 | 0.900 | 0.201 | 0.944 |
| rpl6 | rpl36a | 1493092 | 1490292 | ENSDARP00000091899 | ENSDARP00000075363 | 0 | 0 | 0 | 0 | 0.955 | 0.962 | 0.900 | 0.510 | 0.999 |
| rps29 | rps3a | 1487533 | 1486104 | ENSDARP00000060443 | ENSDARP00000051762 | 0 | 0 | 0 | 0 | 0.997 | 0.966 | 0.900 | 0.473 | 0.999 |
| rpl18a | rps23 | 1484877 | 1484618 | ENSDARP00000038658 | ENSDARP00000035273 | 0 | 0 | 0 | 0 | 0.979 | 0.708 | 0.900 | 0.152 | 0.999 |
| rpsa | rpl5a | 1506145 | 1481770 | ENSDARP00000123183 | ENSDARP00000006085 | 0.078 | 0 | 0 | 0 | 0.999 | 0.204 | 0.900 | 0.367 | 0.999 |
| ns:zf-e68 | myl1 | 1501354 | 1481643 | ENSDARP00000111326 | ENSDARP00000004932 | 0 | 0 | 0 | 0 | 0.977 | 0.203 | 0 | 0.231 | 0.985 |
| gapdh | tpi1a | 1488083 | 1484521 | ENSDARP00000063799 | ENSDARP00000033907 | 0.493 | 0.082 | 0 | 0 | 0.608 | 0.320 | 0.957 | 0.518 | 0.996 |
| pdlim7 | tpma | 1485440 | 1484960 | ENSDARP00000044908 | ENSDARP00000039656 | 0 | 0 | 0 | 0 | 0.965 | 0.114 | 0 | 0.108 | 0.971 |
| rps29 | rpl28 | 1487533 | 1483702 | ENSDARP00000060443 | ENSDARP00000024189 | 0 | 0 | 0 | 0 | 0.997 | 0.133 | 0.900 | 0.721 | 0.999 |
| eef1a1a | rpl14 | 1497414 | 1486227 | ENSDARP00000104468 | ENSDARP00000052528 | 0 | 0 | 0 | 0 | 0.197 | 0.121 | 0.900 | 0.114 | 0.929 |
| pkmb | eno3 | 1505987 | 1505202 | ENSDARP00000122764 | ENSDARP00000120742 | 0.257 | 0 | 0 | 0 | 0.994 | 0.128 | 0.957 | 0.608 | 0.999 |
| rock2a | mylpfb | 1505939 | 1483572 | ENSDARP00000122621 | ENSDARP00000023063 | 0 | 0 | 0 | 0 | 0 | 0.072 | 0.800 | 0.723 | 0.944 |
| EIF3F | rpl6 | 1495369 | 1493092 | ENSDARP00000099664 | ENSDARP00000091899 | 0 | 0 | 0 | 0 | 0.727 | 0 | 0.900 | 0 | 0.972 |
| aldoaa | ldha | 1504686 | 1487429 | ENSDARP00000119413 | ENSDARP00000059885 | 0 | 0 | 0 | 0 | 0.449 | 0 | 0.800 | 0.641 | 0.959 |
| rpl19 | rpl11 | 1498085 | 1488101 | ENSDARP00000105649 | ENSDARP00000063869 | 0.493 | 0 | 0 | 0 | 0.994 | 0.978 | 0.900 | 0.265 | 0.999 |
| rps23 | rbm8a | 1484618 | 1483952 | ENSDARP00000035273 | ENSDARP00000026575 | 0 | 0 | 0 | 0 | 0.170 | 0 | 0.900 | 0 | 0.915 |
| uqcrc1 | cox4i2 | 1499876 | 1493898 | ENSDARP00000108798 | ENSDARP00000095260 | 0 | 0 | 0 | 0 | 0.481 | 0.474 | 0.372 | 0.590 | 0.922 |
| rpsa | rps26l | 1506145 | 1501604 | ENSDARP00000123183 | ENSDARP00000111782 | 0 | 0 | 0 | 0 | 0.994 | 0.860 | 0.900 | 0.120 | 0.999 |
| rps26l | eif4eb | 1501604 | 1481876 | ENSDARP00000111782 | ENSDARP00000007117 | 0 | 0 | 0 | 0 | 0.111 | 0 | 0.900 | 0.232 | 0.925 |
| spcs1 | rps27.2 | 1490634 | 1489628 | ENSDARP00000076814 | ENSDARP00000072300 | 0 | 0 | 0 | 0 | 0.117 | 0 | 0.900 | 0 | 0.910 |
| rpsa | eif4a3 | 1506145 | 1484026 | ENSDARP00000123183 | ENSDARP00000027276 | 0 | 0 | 0 | 0 | 0.333 | 0.165 | 0.900 | 0.388 | 0.963 |
| rpl22 | eef1a1a | 1501438 | 1497414 | ENSDARP00000111487 | ENSDARP00000104468 | 0 | 0 | 0 | 0 | 0.106 | 0 | 0.900 | 0 | 0.906 |
| rpsa | eef2l2 | 1506145 | 1486009 | ENSDARP00000123183 | ENSDARP00000051080 | 0.083 | 0 | 0 | 0 | 0.842 | 0.837 | 0 | 0.330 | 0.982 |
| rpsa | rps23 | 1506145 | 1484618 | ENSDARP00000123183 | ENSDARP00000035273 | 0.090 | 0 | 0 | 0 | 0.973 | 0.977 | 0.900 | 0.622 | 0.999 |
| rpl18a | eif4a3 | 1484877 | 1484026 | ENSDARP00000038658 | ENSDARP00000027276 | 0 | 0 | 0 | 0 | 0.320 | 0.202 | 0.900 | 0.247 | 0.954 |
| pfdn2 | zgc:65894 | 1484617 | 1481340 | ENSDARP00000035261 | ENSDARP00000002175 | 0 | 0 | 0 | 0 | 0 | 0.318 | 0.900 | 0 | 0.928 |
| rps26 | rps3a | 1497897 | 1486104 | ENSDARP00000105328 | ENSDARP00000051762 | 0 | 0 | 0 | 0 | 0.999 | 0.861 | 0.900 | 0.225 | 0.999 |
| atp5h | atp5l | 1484892 | 1481947 | ENSDARP00000038799 | ENSDARP00000007716 | 0 | 0 | 0 | 0 | 0.866 | 0.360 | 0.918 | 0.364 | 0.995 |
| spcs1 | rpl13 | 1490634 | 1485678 | ENSDARP00000076814 | ENSDARP00000047390 | 0 | 0 | 0 | 0 | 0.350 | 0 | 0.900 | 0 | 0.932 |
| rps10 | rps23 | 1485542 | 1484618 | ENSDARP00000045900 | ENSDARP00000035273 | 0 | 0 | 0 | 0 | 0.943 | 0.951 | 0.900 | 0.563 | 0.999 |
| rps26l | rpl35 | 1501604 | 1483123 | ENSDARP00000111782 | ENSDARP00000018594 | 0 | 0 | 0 | 0 | 0.997 | 0 | 0.900 | 0.196 | 0.999 |
| rpl23 | rps16 | 1489208 | 1488590 | ENSDARP00000069977 | ENSDARP00000066897 | 0.243 | 0 | 0 | 0 | 0.999 | 0.692 | 0.900 | 0.526 | 0.999 |
| rpl36a | rps3 | 1490292 | 1488779 | ENSDARP00000075363 | ENSDARP00000067802 | 0 | 0 | 0 | 0 | 0.998 | 0 | 0.900 | 0.242 | 0.999 |
| myl2 | tnni2a.1 | 1503455 | 1484340 | ENSDARP00000116241 | ENSDARP00000031650 | 0 | 0 | 0 | 0 | 0.167 | 0.187 | 0.900 | 0.334 | 0.948 |
| rpl6 | eif4eb | 1493092 | 1481876 | ENSDARP00000091899 | ENSDARP00000007117 | 0 | 0 | 0 | 0 | 0.156 | 0 | 0.900 | 0 | 0.911 |
| rpl19 | rpl23 | 1498085 | 1489208 | ENSDARP00000105649 | ENSDARP00000069977 | 0.493 | 0 | 0 | 0 | 0.998 | 0.978 | 0.900 | 0.330 | 0.999 |
| tnnt3b | myl1 | 1497962 | 1481643 | ENSDARP00000105443 | ENSDARP00000004932 | 0 | 0 | 0 | 0 | 0.875 | 0.176 | 0.900 | 0.150 | 0.990 |
| rpsa | rps27.1 | 1506145 | 1484170 | ENSDARP00000123183 | ENSDARP00000029079 | 0 | 0 | 0 | 0 | 0.998 | 0.797 | 0.900 | 0.330 | 0.999 |
| eef2l2 | rpl7 | 1486009 | 1483164 | ENSDARP00000051080 | ENSDARP00000018980 | 0.257 | 0 | 0 | 0 | 0.815 | 0.482 | 0 | 0.234 | 0.938 |
| rps24 | rpl32 | 1493019 | 1487450 | ENSDARP00000091586 | ENSDARP00000060004 | 0 | 0 | 0 | 0 | 0.911 | 0.793 | 0.900 | 0.118 | 0.998 |
| rpl19 | rpl3 | 1498085 | 1481524 | ENSDARP00000105649 | ENSDARP00000003700 | 0.493 | 0 | 0 | 0 | 0.978 | 0.966 | 0.900 | 0.330 | 0.999 |
| dnaja2 | hsp90aa1.2 | 1484139 | 1483899 | ENSDARP00000028641 | ENSDARP00000026065 | 0 | 0 | 0 | 0 | 0.690 | 0.849 | 0 | 0.812 | 0.990 |
| rpl18a | rpl3 | 1484877 | 1481524 | ENSDARP00000038658 | ENSDARP00000003700 | 0 | 0 | 0 | 0 | 0.997 | 0.961 | 0.900 | 0.193 | 0.999 |
| rpl36a | rps16 | 1490292 | 1488590 | ENSDARP00000075363 | ENSDARP00000066897 | 0 | 0 | 0 | 0 | 0.999 | 0.456 | 0.900 | 0 | 0.999 |
| pkmb | gpib | 1505987 | 1482688 | ENSDARP00000122764 | ENSDARP00000014578 | 0.095 | 0 | 0 | 0 | 0.798 | 0 | 0.800 | 0.573 | 0.982 |
| rpl22 | rpl6 | 1501438 | 1493092 | ENSDARP00000111487 | ENSDARP00000091899 | 0 | 0 | 0 | 0 | 0.744 | 0.974 | 0.900 | 0.241 | 0.999 |
| LOC567740 | myl2 | 1505894 | 1503455 | ENSDARP00000122502 | ENSDARP00000116241 | 0 | 0 | 0 | 0 | 0.085 | 0.288 | 0.900 | 0.388 | 0.954 |
| rps26l | rpl13 | 1501604 | 1485678 | ENSDARP00000111782 | ENSDARP00000047390 | 0 | 0 | 0 | 0 | 0.997 | 0 | 0.900 | 0.470 | 0.999 |
| ddost | rps23 | 1486503 | 1484618 | ENSDARP00000054289 | ENSDARP00000035273 | 0 | 0 | 0 | 0 | 0 | 0 | 0.900 | 0 | 0.900 |
| eef2l2 | rps23 | 1486009 | 1484618 | ENSDARP00000051080 | ENSDARP00000035273 | 0.493 | 0 | 0 | 0 | 0.853 | 0.861 | 0 | 0.330 | 0.992 |
| rpl14 | rpl3 | 1486227 | 1481524 | ENSDARP00000052528 | ENSDARP00000003700 | 0.071 | 0 | 0 | 0 | 0.965 | 0.961 | 0.900 | 0.238 | 0.999 |
| eif2s1 | rps29 | 1488921 | 1487533 | ENSDARP00000068470 | ENSDARP00000060443 | 0 | 0 | 0 | 0 | 0.093 | 0 | 0.900 | 0 | 0.905 |
| rps26 | rpl35 | 1497897 | 1483123 | ENSDARP00000105328 | ENSDARP00000018594 | 0 | 0 | 0 | 0 | 0.997 | 0 | 0.900 | 0.196 | 0.999 |
| rpl32 | rpl35 | 1487450 | 1483123 | ENSDARP00000060004 | ENSDARP00000018594 | 0.493 | 0 | 0 | 0 | 0.996 | 0.965 | 0.900 | 0.201 | 0.999 |
| rps27.1 | nudc | 1484170 | 1481667 | ENSDARP00000029079 | ENSDARP00000005181 | 0 | 0 | 0 | 0 | 0 | 0 | 0.900 | 0 | 0.900 |
| rps26 | rpl7 | 1497897 | 1483164 | ENSDARP00000105328 | ENSDARP00000018980 | 0 | 0 | 0 | 0 | 0.949 | 0.298 | 0.900 | 0.110 | 0.996 |
| actn2 | desmb | 1494007 | 1488347 | ENSDARP00000095652 | ENSDARP00000065355 | 0 | 0 | 0 | 0 | 0 | 0.084 | 0.900 | 0.072 | 0.907 |
| psma3 | psma1 | 1499751 | 1497161 | ENSDARP00000108560 | ENSDARP00000103895 | 0 | 0 | 0.298 | 0.760 | 0.725 | 0.978 | 0.964 | 0.614 | 0.999 |
| rps3 | rps29 | 1488779 | 1487533 | ENSDARP00000067802 | ENSDARP00000060443 | 0.493 | 0 | 0 | 0 | 0.993 | 0.978 | 0.900 | 0.330 | 0.999 |
| rpl28 | eif4eb | 1483702 | 1481876 | ENSDARP00000024189 | ENSDARP00000007117 | 0 | 0 | 0 | 0 | 0.072 | 0 | 0.900 | 0 | 0.903 |
| rpl36a | rpl3 | 1490292 | 1481524 | ENSDARP00000075363 | ENSDARP00000003700 | 0 | 0 | 0 | 0 | 0.948 | 0.964 | 0.900 | 0.518 | 0.999 |
| rps26l | rplp2 | 1501604 | 1483852 | ENSDARP00000111782 | ENSDARP00000025616 | 0 | 0 | 0 | 0 | 0.989 | 0.341 | 0 | 0.096 | 0.993 |
| rps26 | rps3 | 1497897 | 1488779 | ENSDARP00000105328 | ENSDARP00000067802 | 0 | 0 | 0 | 0 | 0.997 | 0.861 | 0.900 | 0.573 | 0.999 |
| LOC567740 | smyhc1 | 1505894 | 1486922 | ENSDARP00000122502 | ENSDARP00000056852 | 0 | 0 | 0 | 0.989 | 0 | 0 | 0.900 | 0 | 0.900 |
| rpsa | rps24 | 1506145 | 1493019 | ENSDARP00000123183 | ENSDARP00000091586 | 0 | 0 | 0 | 0 | 0.794 | 0.967 | 0.900 | 0.151 | 0.999 |
| rps3 | rps10 | 1488779 | 1485542 | ENSDARP00000067802 | ENSDARP00000045900 | 0 | 0 | 0 | 0 | 0.971 | 0.973 | 0.900 | 0.400 | 0.999 |
| rpl6 | rps16 | 1493092 | 1488590 | ENSDARP00000091899 | ENSDARP00000066897 | 0.068 | 0 | 0 | 0 | 0.973 | 0.822 | 0.900 | 0.370 | 0.999 |
| rpl19 | eif4a3 | 1498085 | 1484026 | ENSDARP00000105649 | ENSDARP00000027276 | 0 | 0 | 0 | 0 | 0.172 | 0.191 | 0.900 | 0 | 0.927 |
| EIF3F | rps24 | 1495369 | 1493019 | ENSDARP00000099664 | ENSDARP00000091586 | 0 | 0 | 0 | 0 | 0.546 | 0.072 | 0.900 | 0 | 0.955 |
| rps27.2 | eif2s1 | 1489628 | 1488921 | ENSDARP00000072300 | ENSDARP00000068470 | 0.493 | 0 | 0 | 0 | 0.097 | 0.080 | 0.900 | 0 | 0.952 |
| MYL3 | tnni2a.1 | 1488531 | 1484340 | ENSDARP00000066500 | ENSDARP00000031650 | 0 | 0 | 0 | 0 | 0.099 | 0.187 | 0.900 | 0.181 | 0.931 |
| tpi1a | eno1a | 1484521 | 1481528 | ENSDARP00000033907 | ENSDARP00000003738 | 0.493 | 0 | 0 | 0 | 0.944 | 0.286 | 0 | 0.628 | 0.991 |
| rpl13 | rps23 | 1485678 | 1484618 | ENSDARP00000047390 | ENSDARP00000035273 | 0 | 0 | 0 | 0 | 0.985 | 0.670 | 0.900 | 0.068 | 0.999 |
| LOC567740 | tnni2a.4 | 1505894 | 1484806 | ENSDARP00000122502 | ENSDARP00000037759 | 0 | 0 | 0 | 0 | 0.117 | 0.188 | 0.900 | 0.513 | 0.960 |
| psma1 | smurf1 | 1497161 | 1481527 | ENSDARP00000103895 | ENSDARP00000003721 | 0 | 0 | 0 | 0 | 0 | 0.133 | 0.900 | 0.068 | 0.912 |
| aldoab | myl1 | 1485195 | 1481643 | ENSDARP00000042199 | ENSDARP00000004932 | 0 | 0 | 0 | 0 | 0.917 | 0.070 | 0 | 0.096 | 0.924 |
| rps24 | rpl11 | 1493019 | 1488101 | ENSDARP00000091586 | ENSDARP00000063869 | 0 | 0 | 0 | 0 | 0.892 | 0.861 | 0.900 | 0.618 | 0.999 |
| rpl14 | rps3a | 1486227 | 1486104 | ENSDARP00000052528 | ENSDARP00000051762 | 0 | 0 | 0 | 0 | 0.995 | 0.875 | 0.900 | 0.217 | 0.999 |
| rps16 | rplp2 | 1488590 | 1483852 | ENSDARP00000066897 | ENSDARP00000025616 | 0 | 0 | 0 | 0 | 0.999 | 0.796 | 0 | 0.114 | 0.999 |
| tnnc2 | tnnc1a | 1493861 | 1483843 | ENSDARP00000095111 | ENSDARP00000025541 | 0 | 0 | 0 | 0.946 | 0.105 | 0 | 0.900 | 0.334 | 0.908 |
| tnni2a.4 | tnnc1a | 1484806 | 1483843 | ENSDARP00000037759 | ENSDARP00000025541 | 0 | 0 | 0 | 0 | 0.079 | 0.284 | 0.900 | 0.210 | 0.940 |
| rpl6 | spcs1 | 1493092 | 1490634 | ENSDARP00000091899 | ENSDARP00000076814 | 0 | 0 | 0 | 0 | 0.125 | 0 | 0.900 | 0 | 0.910 |
| rpl11 | eif4eb | 1488101 | 1481876 | ENSDARP00000063869 | ENSDARP00000007117 | 0 | 0 | 0 | 0 | 0.165 | 0 | 0.900 | 0.144 | 0.922 |
| ns:zf-e68 | tnnc2 | 1501354 | 1493861 | ENSDARP00000111326 | ENSDARP00000095111 | 0 | 0 | 0 | 0 | 0.717 | 0.200 | 0 | 0.659 | 0.920 |
| tnnt3b | mylz3 | 1497962 | 1483092 | ENSDARP00000105443 | ENSDARP00000018197 | 0 | 0 | 0 | 0 | 0.982 | 0.176 | 0.900 | 0.375 | 0.999 |
| desmb | tnni2a.4 | 1488347 | 1484806 | ENSDARP00000065355 | ENSDARP00000037759 | 0 | 0 | 0 | 0 | 0 | 0 | 0.900 | 0.064 | 0.902 |
| psma2 | smurf1 | 1487227 | 1481527 | ENSDARP00000058679 | ENSDARP00000003721 | 0 | 0 | 0 | 0 | 0 | 0.070 | 0.900 | 0.084 | 0.907 |
| EIF3F | rpl7 | 1495369 | 1483164 | ENSDARP00000099664 | ENSDARP00000018980 | 0 | 0 | 0 | 0 | 0.558 | 0 | 0.900 | 0 | 0.955 |
| myl2 | myl1 | 1503455 | 1481643 | ENSDARP00000116241 | ENSDARP00000004932 | 0 | 0 | 0 | 0 | 0.116 | 0.072 | 0.900 | 0.358 | 0.941 |
| rpl23 | rps27.1 | 1489208 | 1484170 | ENSDARP00000069977 | ENSDARP00000029079 | 0 | 0 | 0 | 0 | 0.997 | 0.450 | 0.900 | 0.330 | 0.999 |
| rpl23 | ddost | 1489208 | 1486503 | ENSDARP00000069977 | ENSDARP00000054289 | 0 | 0 | 0 | 0 | 0 | 0 | 0.900 | 0 | 0.900 |
| tnnc2 | mylz3 | 1493861 | 1483092 | ENSDARP00000095111 | ENSDARP00000018197 | 0 | 0 | 0 | 0.746 | 0.980 | 0 | 0.900 | 0.486 | 0.998 |
| eno3 | pgk1 | 1505202 | 1489379 | ENSDARP00000120742 | ENSDARP00000070807 | 0.493 | 0 | 0.205 | 0 | 0.933 | 0.638 | 0 | 0.674 | 0.996 |
| rpl23 | rps10 | 1489208 | 1485542 | ENSDARP00000069977 | ENSDARP00000045900 | 0 | 0 | 0 | 0 | 0.984 | 0 | 0.900 | 0.149 | 0.998 |
| aldoaa | pgk1 | 1504686 | 1489379 | ENSDARP00000119413 | ENSDARP00000070807 | 0.378 | 0 | 0 | 0 | 0.521 | 0.177 | 0.408 | 0.577 | 0.930 |
| pkmb | aldoab | 1505987 | 1485195 | ENSDARP00000122764 | ENSDARP00000042199 | 0.069 | 0 | 0 | 0 | 0.693 | 0 | 0.800 | 0.608 | 0.974 |
| rps3a | rpl35 | 1486104 | 1483123 | ENSDARP00000051762 | ENSDARP00000018594 | 0 | 0 | 0 | 0 | 0.999 | 0.875 | 0.900 | 0.097 | 0.999 |
| rps3 | rpl14 | 1488779 | 1486227 | ENSDARP00000067802 | ENSDARP00000052528 | 0.083 | 0 | 0 | 0 | 0.993 | 0.874 | 0.900 | 0.719 | 0.999 |
| aldoab | mylz3 | 1485195 | 1483092 | ENSDARP00000042199 | ENSDARP00000018197 | 0 | 0 | 0 | 0 | 0.899 | 0.070 | 0 | 0.096 | 0.907 |
| rpl22 | spcs1 | 1501438 | 1490634 | ENSDARP00000111487 | ENSDARP00000076814 | 0 | 0 | 0 | 0 | 0.446 | 0 | 0.900 | 0 | 0.942 |
| rps26l | rps26 | 1501604 | 1497897 | ENSDARP00000111782 | ENSDARP00000105328 | 0 | 0 | 0.534 | 0.988 | 0.961 | 0 | 0.900 | 0 | 0.995 |
| rpl19 | EIF3F | 1498085 | 1495369 | ENSDARP00000105649 | ENSDARP00000099664 | 0 | 0 | 0 | 0 | 0.505 | 0.139 | 0.900 | 0 | 0.954 |
| snrpc | snrpb | 1499628 | 1483602 | ENSDARP00000108355 | ENSDARP00000023337 | 0 | 0 | 0 | 0 | 0.800 | 0.952 | 0 | 0.601 | 0.995 |
| rps16 | rpl3 | 1488590 | 1481524 | ENSDARP00000066897 | ENSDARP00000003700 | 0.186 | 0 | 0 | 0 | 0.997 | 0.859 | 0.900 | 0.330 | 0.999 |
| tnnt3b | actn2 | 1497962 | 1494007 | ENSDARP00000105443 | ENSDARP00000095652 | 0 | 0 | 0 | 0 | 0.486 | 0.140 | 0.900 | 0.359 | 0.967 |
| eef1a1a | rpl32 | 1497414 | 1487450 | ENSDARP00000104468 | ENSDARP00000060004 | 0 | 0 | 0 | 0 | 0.358 | 0 | 0.900 | 0.088 | 0.936 |
| rpl13 | rbm8a | 1485678 | 1483952 | ENSDARP00000047390 | ENSDARP00000026575 | 0 | 0 | 0 | 0 | 0.103 | 0 | 0.900 | 0 | 0.906 |
| rps26 | eif4a3 | 1497897 | 1484026 | ENSDARP00000105328 | ENSDARP00000027276 | 0 | 0 | 0 | 0 | 0.262 | 0.111 | 0.900 | 0.071 | 0.930 |
| rps26l | rps27.2 | 1501604 | 1489628 | ENSDARP00000111782 | ENSDARP00000072300 | 0 | 0 | 0 | 0 | 0.995 | 0.678 | 0.900 | 0.380 | 0.999 |
| rpl19 | rps3a | 1498085 | 1486104 | ENSDARP00000105649 | ENSDARP00000051762 | 0 | 0 | 0 | 0 | 0.999 | 0.841 | 0.900 | 0.347 | 0.999 |
| tnnc2 | tnni2a.1 | 1493861 | 1484340 | ENSDARP00000095111 | ENSDARP00000031650 | 0 | 0 | 0 | 0 | 0.691 | 0.788 | 0.900 | 0.462 | 0.996 |
| rps27.2 | rpl14 | 1489628 | 1486227 | ENSDARP00000072300 | ENSDARP00000052528 | 0 | 0 | 0 | 0 | 0.995 | 0.450 | 0.900 | 0.152 | 0.999 |
| rpl13 | rpl28 | 1485678 | 1483702 | ENSDARP00000047390 | ENSDARP00000024189 | 0 | 0 | 0 | 0 | 0.999 | 0.955 | 0.900 | 0.151 | 0.999 |
| pkmb | eno2 | 1505987 | 1484405 | ENSDARP00000122764 | ENSDARP00000032456 | 0.257 | 0 | 0 | 0 | 0.994 | 0.128 | 0.957 | 0.608 | 0.999 |
| rps16 | rpl7 | 1488590 | 1483164 | ENSDARP00000066897 | ENSDARP00000018980 | 0.234 | 0 | 0 | 0 | 0.963 | 0.874 | 0.900 | 0.609 | 0.999 |
| tpma | myl1 | 1484960 | 1481643 | ENSDARP00000039656 | ENSDARP00000004932 | 0 | 0 | 0 | 0 | 0.984 | 0.134 | 0 | 0.220 | 0.988 |
| rpsa | rpl6 | 1506145 | 1493092 | ENSDARP00000123183 | ENSDARP00000091899 | 0 | 0 | 0 | 0 | 0.995 | 0.245 | 0.900 | 0 | 0.999 |
| vbp1 | pfdn2 | 1501624 | 1484617 | ENSDARP00000111810 | ENSDARP00000035261 | 0 | 0 | 0 | 0 | 0.242 | 0.975 | 0.900 | 0.612 | 0.999 |
| actn2 | MYL3 | 1494007 | 1488531 | ENSDARP00000095652 | ENSDARP00000066500 | 0 | 0 | 0 | 0 | 0 | 0.070 | 0.900 | 0.308 | 0.931 |
| rpl14 | rpl35 | 1486227 | 1483123 | ENSDARP00000052528 | ENSDARP00000018594 | 0.075 | 0 | 0 | 0 | 0.991 | 0.973 | 0.900 | 0.201 | 0.999 |
| tnnc2 | desma | 1493861 | 1490431 | ENSDARP00000095111 | ENSDARP00000075994 | 0 | 0 | 0 | 0 | 0.949 | 0.071 | 0.900 | 0.269 | 0.996 |
| rpl6 | rbm8a | 1493092 | 1483952 | ENSDARP00000091899 | ENSDARP00000026575 | 0 | 0 | 0 | 0 | 0.098 | 0 | 0.900 | 0 | 0.905 |
| atp5c1 | atp5h | 1488594 | 1484892 | ENSDARP00000066929 | ENSDARP00000038799 | 0 | 0 | 0 | 0 | 0.993 | 0.938 | 0.932 | 0.365 | 0.999 |
| rps26l | ddost | 1501604 | 1486503 | ENSDARP00000111782 | ENSDARP00000054289 | 0 | 0 | 0 | 0 | 0.091 | 0 | 0.900 | 0 | 0.905 |
| gapdh | aldocb | 1488083 | 1483738 | ENSDARP00000063799 | ENSDARP00000024492 | 0.257 | 0 | 0 | 0 | 0.278 | 0.206 | 0.949 | 0.384 | 0.985 |
| aldocb | pgm1 | 1483738 | 1481818 | ENSDARP00000024492 | ENSDARP00000006510 | 0 | 0 | 0 | 0 | 0.610 | 0 | 0.800 | 0.317 | 0.944 |
| eif4a3 | rpl35 | 1484026 | 1483123 | ENSDARP00000027276 | ENSDARP00000018594 | 0 | 0 | 0 | 0 | 0.067 | 0 | 0.900 | 0.122 | 0.910 |
| EIF3F | rps10 | 1495369 | 1485542 | ENSDARP00000099664 | ENSDARP00000045900 | 0 | 0 | 0 | 0 | 0.734 | 0.101 | 0.900 | 0 | 0.974 |
| rps3a | rpl5a | 1486104 | 1481770 | ENSDARP00000051762 | ENSDARP00000006085 | 0 | 0 | 0 | 0 | 0.999 | 0.201 | 0.900 | 0.589 | 0.999 |
| tnnc2 | ckmb | 1493861 | 1487353 | ENSDARP00000095111 | ENSDARP00000059365 | 0 | 0 | 0 | 0 | 0.984 | 0.079 | 0 | 0 | 0.985 |
| rpl14 | eif4eb | 1486227 | 1481876 | ENSDARP00000052528 | ENSDARP00000007117 | 0 | 0 | 0 | 0 | 0.164 | 0 | 0.900 | 0 | 0.912 |
| tpi1a | aldocb | 1484521 | 1483738 | ENSDARP00000033907 | ENSDARP00000024492 | 0 | 0 | 0 | 0 | 0.508 | 0 | 0.952 | 0.611 | 0.990 |
| rpl13 | eif4eb | 1485678 | 1481876 | ENSDARP00000047390 | ENSDARP00000007117 | 0 | 0 | 0 | 0 | 0.105 | 0 | 0.900 | 0 | 0.906 |
| rpl19 | rps16 | 1498085 | 1488590 | ENSDARP00000105649 | ENSDARP00000066897 | 0 | 0 | 0 | 0 | 0.999 | 0.873 | 0.900 | 0.244 | 0.999 |
| rpl6 | rpl5a | 1493092 | 1481770 | ENSDARP00000091899 | ENSDARP00000006085 | 0.167 | 0 | 0 | 0 | 0.993 | 0.870 | 0.900 | 0.562 | 0.999 |
| eef1a1a | rps24 | 1497414 | 1493019 | ENSDARP00000104468 | ENSDARP00000091586 | 0 | 0 | 0 | 0 | 0.200 | 0.164 | 0.900 | 0 | 0.927 |
| rpl5a | rpl3 | 1481770 | 1481524 | ENSDARP00000006085 | ENSDARP00000003700 | 0.493 | 0 | 0.315 | 0 | 0.999 | 0.865 | 0.900 | 0.644 | 0.999 |
| rps29 | rpl14 | 1487533 | 1486227 | ENSDARP00000060443 | ENSDARP00000052528 | 0.098 | 0 | 0 | 0 | 0.997 | 0.278 | 0.900 | 0.088 | 0.999 |
| rps27.1 | rpl7 | 1484170 | 1483164 | ENSDARP00000029079 | ENSDARP00000018980 | 0 | 0 | 0 | 0 | 0.971 | 0.450 | 0.900 | 0.643 | 0.999 |
| eef1a1a | rps3a | 1497414 | 1486104 | ENSDARP00000104468 | ENSDARP00000051762 | 0 | 0 | 0 | 0 | 0.514 | 0.149 | 0.900 | 0.374 | 0.970 |
| tnnt3b | desma | 1497962 | 1490431 | ENSDARP00000105443 | ENSDARP00000075994 | 0 | 0 | 0 | 0 | 0.323 | 0 | 0.900 | 0 | 0.931 |
| rpl36a | eif2s1 | 1490292 | 1488921 | ENSDARP00000075363 | ENSDARP00000068470 | 0.493 | 0 | 0 | 0 | 0.116 | 0 | 0.900 | 0.200 | 0.959 |
| rpsa | rps26 | 1506145 | 1497897 | ENSDARP00000123183 | ENSDARP00000105328 | 0 | 0 | 0 | 0 | 0.998 | 0.860 | 0.900 | 0.195 | 0.999 |
| rpl22 | rps27.2 | 1501438 | 1489628 | ENSDARP00000111487 | ENSDARP00000072300 | 0 | 0 | 0 | 0 | 0.788 | 0.452 | 0.900 | 0.214 | 0.989 |
| rps26 | rps16 | 1497897 | 1488590 | ENSDARP00000105328 | ENSDARP00000066897 | 0 | 0 | 0 | 0 | 0.999 | 0.858 | 0.900 | 0.121 | 0.999 |
| rps29 | rpl18a | 1487533 | 1484877 | ENSDARP00000060443 | ENSDARP00000038658 | 0 | 0 | 0 | 0 | 0.995 | 0 | 0.900 | 0.369 | 0.999 |
| rps26l | rpl6 | 1501604 | 1493092 | ENSDARP00000111782 | ENSDARP00000091899 | 0 | 0 | 0 | 0 | 0.986 | 0.202 | 0.900 | 0.146 | 0.998 |
| rps24 | rpl23 | 1493019 | 1489208 | ENSDARP00000091586 | ENSDARP00000069977 | 0 | 0 | 0 | 0 | 0.841 | 0.830 | 0.900 | 0.141 | 0.997 |
| pvalb1 | mylz3 | 1486619 | 1483092 | ENSDARP00000055061 | ENSDARP00000018197 | 0 | 0 | 0 | 0 | 0.980 | 0 | 0 | 0 | 0.980 |
| rps10 | rpl3 | 1485542 | 1481524 | ENSDARP00000045900 | ENSDARP00000003700 | 0 | 0 | 0 | 0 | 0.977 | 0 | 0.900 | 0.236 | 0.998 |
| eef1a1l1 | rpl3 | 1501580 | 1481524 | ENSDARP00000111742 | ENSDARP00000003700 | 0 | 0 | 0.222 | 0 | 0.993 | 0 | 0 | 0.362 | 0.996 |
| NDUFC2 | cox7c | 1500789 | 1489195 | ENSDARP00000110392 | ENSDARP00000069896 | 0 | 0 | 0 | 0 | 0.976 | 0 | 0 | 0.213 | 0.981 |
| tnni2a.4 | tnni2a.1 | 1484806 | 1484340 | ENSDARP00000037759 | ENSDARP00000031650 | 0 | 0 | 0 | 0.974 | 0 | 0 | 0.900 | 0.655 | 0.901 |
| rpl14 | rps23 | 1486227 | 1484618 | ENSDARP00000052528 | ENSDARP00000035273 | 0 | 0 | 0 | 0 | 0.995 | 0.692 | 0.900 | 0.126 | 0.999 |
| EIF3F | eif2s1 | 1495369 | 1488921 | ENSDARP00000099664 | ENSDARP00000068470 | 0 | 0 | 0 | 0 | 0.168 | 0 | 0.900 | 0.072 | 0.916 |
| rpsa | rpl14 | 1506145 | 1486227 | ENSDARP00000123183 | ENSDARP00000052528 | 0 | 0 | 0 | 0 | 0.981 | 0.465 | 0.900 | 0.068 | 0.998 |
| rps27.1 | rpl28 | 1484170 | 1483702 | ENSDARP00000029079 | ENSDARP00000024189 | 0 | 0 | 0 | 0 | 0.998 | 0 | 0.900 | 0.118 | 0.999 |
| rps3a | rplp2 | 1486104 | 1483852 | ENSDARP00000051762 | ENSDARP00000025616 | 0 | 0 | 0 | 0 | 0.995 | 0.796 | 0 | 0.706 | 0.999 |
| ddost | rps10 | 1486503 | 1485542 | ENSDARP00000054289 | ENSDARP00000045900 | 0 | 0 | 0 | 0 | 0.093 | 0 | 0.900 | 0 | 0.905 |
| rpl19 | rpl14 | 1498085 | 1486227 | ENSDARP00000105649 | ENSDARP00000052528 | 0.135 | 0 | 0 | 0 | 0.980 | 0.972 | 0.900 | 0.363 | 0.999 |
| ckma | mylz3 | 1484819 | 1483092 | ENSDARP00000037871 | ENSDARP00000018197 | 0 | 0 | 0 | 0 | 0.982 | 0.071 | 0 | 0.507 | 0.991 |
| rpl18a | rpl28 | 1484877 | 1483702 | ENSDARP00000038658 | ENSDARP00000024189 | 0 | 0 | 0 | 0 | 0.999 | 0.896 | 0.900 | 0.209 | 0.999 |
| rpl35 | rpl5a | 1483123 | 1481770 | ENSDARP00000018594 | ENSDARP00000006085 | 0.493 | 0 | 0 | 0 | 0.999 | 0.867 | 0.900 | 0.330 | 0.999 |
| rpl22 | rps26 | 1501438 | 1497897 | ENSDARP00000111487 | ENSDARP00000105328 | 0 | 0 | 0 | 0 | 0.986 | 0.382 | 0.900 | 0.607 | 0.999 |
| spcs1 | rps29 | 1490634 | 1487533 | ENSDARP00000076814 | ENSDARP00000060443 | 0 | 0 | 0 | 0 | 0.707 | 0 | 0.900 | 0 | 0.970 |
| rps27.2 | ddost | 1489628 | 1486503 | ENSDARP00000072300 | ENSDARP00000054289 | 0 | 0 | 0 | 0 | 0 | 0 | 0.900 | 0 | 0.900 |
| actn2 | tnni2a.4 | 1494007 | 1484806 | ENSDARP00000095652 | ENSDARP00000037759 | 0 | 0 | 0 | 0 | 0.090 | 0.080 | 0.900 | 0.118 | 0.916 |
| eef2l2 | rpl3 | 1486009 | 1481524 | ENSDARP00000051080 | ENSDARP00000003700 | 0.493 | 0 | 0 | 0 | 0.874 | 0.633 | 0 | 0.330 | 0.982 |
| rpl22 | rpl13 | 1501438 | 1485678 | ENSDARP00000111487 | ENSDARP00000047390 | 0 | 0 | 0 | 0 | 0.939 | 0.976 | 0.900 | 0.501 | 0.999 |
| rpl13 | rps10 | 1485678 | 1485542 | ENSDARP00000047390 | ENSDARP00000045900 | 0 | 0 | 0 | 0 | 0.982 | 0 | 0.900 | 0.234 | 0.998 |
| desma | tnni2a.1 | 1490431 | 1484340 | ENSDARP00000075994 | ENSDARP00000031650 | 0 | 0 | 0 | 0 | 0 | 0 | 0.900 | 0.068 | 0.902 |
| ddost | rpl35 | 1486503 | 1483123 | ENSDARP00000054289 | ENSDARP00000018594 | 0 | 0 | 0 | 0 | 0.091 | 0 | 0.900 | 0 | 0.905 |
| rps27.2 | rpl7 | 1489628 | 1483164 | ENSDARP00000072300 | ENSDARP00000018980 | 0 | 0 | 0 | 0 | 0.789 | 0.450 | 0.900 | 0.185 | 0.989 |
| rps26 | eif2s1 | 1497897 | 1488921 | ENSDARP00000105328 | ENSDARP00000068470 | 0 | 0 | 0 | 0 | 0.135 | 0 | 0.900 | 0 | 0.909 |
| rbm19 | ddx56 | 1487837 | 1484656 | ENSDARP00000062458 | ENSDARP00000035846 | 0 | 0 | 0 | 0 | 0.909 | 0 | 0 | 0 | 0.911 |
| NDUFC2 | ndufa12 | 1500789 | 1487803 | ENSDARP00000110392 | ENSDARP00000062277 | 0 | 0 | 0 | 0 | 0.698 | 0 | 0.933 | 0.717 | 0.993 |
| EIF3F | eif4eb | 1495369 | 1481876 | ENSDARP00000099664 | ENSDARP00000007117 | 0 | 0 | 0 | 0 | 0.098 | 0.827 | 0.900 | 0.138 | 0.984 |
| rpl22 | rplp2 | 1501438 | 1483852 | ENSDARP00000111487 | ENSDARP00000025616 | 0 | 0 | 0 | 0 | 0.976 | 0.638 | 0 | 0.088 | 0.991 |
| smyhc1 | actc1b | 1486922 | 1486630 | ENSDARP00000056852 | ENSDARP00000055135 | 0 | 0 | 0 | 0 | 0.864 | 0.294 | 0 | 0.492 | 0.948 |
| tnnt3b | tnni2a.4 | 1497962 | 1484806 | ENSDARP00000105443 | ENSDARP00000037759 | 0 | 0 | 0 | 0 | 0.999 | 0.324 | 0.900 | 0.823 | 0.999 |
| aldocb | eno1a | 1483738 | 1481528 | ENSDARP00000024492 | ENSDARP00000003738 | 0 | 0 | 0 | 0 | 0.991 | 0.070 | 0.800 | 0.459 | 0.999 |
| desma | ckma | 1490431 | 1484819 | ENSDARP00000075994 | ENSDARP00000037871 | 0 | 0 | 0 | 0 | 0.877 | 0 | 0 | 0.293 | 0.909 |
| ldha | aldocb | 1487429 | 1483738 | ENSDARP00000059885 | ENSDARP00000024492 | 0 | 0 | 0 | 0 | 0.387 | 0 | 0.800 | 0.364 | 0.917 |
| gapdhs | eno2 | 1487169 | 1484405 | ENSDARP00000058383 | ENSDARP00000032456 | 0.493 | 0 | 0 | 0 | 0.702 | 0.475 | 0 | 0.603 | 0.964 |
| rps3 | rps16 | 1488779 | 1488590 | ENSDARP00000067802 | ENSDARP00000066897 | 0.231 | 0 | 0 | 0 | 0.999 | 0.978 | 0.900 | 0.535 | 0.999 |
| rpl22 | rps29 | 1501438 | 1487533 | ENSDARP00000111487 | ENSDARP00000060443 | 0 | 0 | 0 | 0 | 0.993 | 0 | 0.900 | 0.352 | 0.999 |
| EIF3F | rpl13 | 1495369 | 1485678 | ENSDARP00000099664 | ENSDARP00000047390 | 0 | 0 | 0 | 0 | 0.750 | 0.070 | 0.900 | 0 | 0.975 |
| hsp90aa1.2 | hsp90aa1.1 | 1483899 | 1483481 | ENSDARP00000026065 | ENSDARP00000022302 | 0 | 0 | 0.532 | 0.985 | 0.323 | 0.477 | 0.900 | 0.907 | 0.963 |
| rps3 | rpl13 | 1488779 | 1485678 | ENSDARP00000067802 | ENSDARP00000047390 | 0 | 0 | 0 | 0 | 0.999 | 0.877 | 0.900 | 0.157 | 0.999 |
| rpl22 | rpl23 | 1501438 | 1489208 | ENSDARP00000111487 | ENSDARP00000069977 | 0 | 0 | 0 | 0 | 0.952 | 0.976 | 0.900 | 0.347 | 0.999 |
| rpl23 | eif2s1 | 1489208 | 1488921 | ENSDARP00000069977 | ENSDARP00000068470 | 0 | 0 | 0 | 0 | 0.121 | 0 | 0.900 | 0 | 0.908 |
| rps3a | rpl28 | 1486104 | 1483702 | ENSDARP00000051762 | ENSDARP00000024189 | 0 | 0 | 0 | 0 | 0.999 | 0.136 | 0.900 | 0.660 | 0.999 |
| tnnc2 | tnni2a.4 | 1493861 | 1484806 | ENSDARP00000095111 | ENSDARP00000037759 | 0 | 0 | 0 | 0 | 0.288 | 0.284 | 0.900 | 0.553 | 0.975 |
| actc1b | myl10 | 1486630 | 1485924 | ENSDARP00000055135 | ENSDARP00000050204 | 0 | 0 | 0 | 0 | 0.940 | 0.180 | 0 | 0.231 | 0.958 |
| gapdh | actc1b | 1488083 | 1486630 | ENSDARP00000063799 | ENSDARP00000055135 | 0 | 0 | 0 | 0 | 0.830 | 0.079 | 0 | 0.558 | 0.924 |
| pgk1 | tpi1a | 1489379 | 1484521 | ENSDARP00000070807 | ENSDARP00000033907 | 0.493 | 0.813 | 0.231 | 0 | 0.964 | 0.326 | 0 | 0.701 | 0.999 |
| ckmb | tpm3 | 1487353 | 1481594 | ENSDARP00000059365 | ENSDARP00000004352 | 0 | 0 | 0 | 0 | 0.906 | 0.072 | 0 | 0.086 | 0.915 |
| LOC567740 | actn2 | 1505894 | 1494007 | ENSDARP00000122502 | ENSDARP00000095652 | 0 | 0 | 0 | 0 | 0.156 | 0.268 | 0.900 | 0.367 | 0.955 |
| eif2s1 | rpl5a | 1488921 | 1481770 | ENSDARP00000068470 | ENSDARP00000006085 | 0 | 0 | 0 | 0 | 0.274 | 0 | 0.900 | 0 | 0.924 |
| rpl23 | rpl18a | 1489208 | 1484877 | ENSDARP00000069977 | ENSDARP00000038658 | 0 | 0 | 0 | 0 | 0.999 | 0.972 | 0.900 | 0.244 | 0.999 |
| me2 | ldha | 1500379 | 1487429 | ENSDARP00000109682 | ENSDARP00000059885 | 0.248 | 0 | 0 | 0 | 0.130 | 0.145 | 0.934 | 0.247 | 0.968 |
| rps16 | rps23 | 1488590 | 1484618 | ENSDARP00000066897 | ENSDARP00000035273 | 0.240 | 0 | 0 | 0 | 0.999 | 0.978 | 0.900 | 0.330 | 0.999 |
| eef1a1a | rpl11 | 1497414 | 1488101 | ENSDARP00000104468 | ENSDARP00000063869 | 0 | 0 | 0 | 0 | 0.290 | 0.150 | 0.900 | 0.175 | 0.943 |
| eef1a1a | rps27.1 | 1497414 | 1484170 | ENSDARP00000104468 | ENSDARP00000029079 | 0 | 0 | 0 | 0 | 0.249 | 0 | 0.900 | 0 | 0.921 |
| eef2l2 | rpl5a | 1486009 | 1481770 | ENSDARP00000051080 | ENSDARP00000006085 | 0.493 | 0 | 0 | 0 | 0.944 | 0.554 | 0 | 0.255 | 0.989 |
| mylz3 | myl1 | 1483092 | 1481643 | ENSDARP00000018197 | ENSDARP00000004932 | 0 | 0 | 0 | 0.983 | 0.983 | 0 | 0.900 | 0.350 | 0.998 |
| gapdhs | aldoab | 1487169 | 1485195 | ENSDARP00000058383 | ENSDARP00000042199 | 0.257 | 0 | 0 | 0 | 0.364 | 0.206 | 0.949 | 0.473 | 0.989 |
| rps27.2 | rps16 | 1489628 | 1488590 | ENSDARP00000072300 | ENSDARP00000066897 | 0 | 0 | 0 | 0 | 0.996 | 0.800 | 0.900 | 0.217 | 0.999 |
| rpl22 | rps27.1 | 1501438 | 1484170 | ENSDARP00000111487 | ENSDARP00000029079 | 0 | 0 | 0 | 0 | 0.956 | 0.452 | 0.900 | 0.214 | 0.997 |
| rps29 | rpl5a | 1487533 | 1481770 | ENSDARP00000060443 | ENSDARP00000006085 | 0.493 | 0 | 0 | 0 | 0.999 | 0 | 0.900 | 0.784 | 0.999 |
| ckmb | ckma | 1487353 | 1484819 | ENSDARP00000059365 | ENSDARP00000037871 | 0 | 0 | 0.534 | 0.988 | 0.983 | 0 | 0 | 0 | 0.983 |
| rpl11 | rps10 | 1488101 | 1485542 | ENSDARP00000063869 | ENSDARP00000045900 | 0 | 0 | 0 | 0 | 0.979 | 0.640 | 0.900 | 0.586 | 0.999 |
| rps26l | rps3a | 1501604 | 1486104 | ENSDARP00000111782 | ENSDARP00000051762 | 0 | 0 | 0 | 0 | 0.999 | 0.861 | 0.900 | 0.077 | 0.999 |
| ckmb | aldoab | 1487353 | 1485195 | ENSDARP00000059365 | ENSDARP00000042199 | 0 | 0 | 0 | 0 | 0.929 | 0.069 | 0 | 0.112 | 0.938 |
| desmb | tnnc1a | 1488347 | 1483843 | ENSDARP00000065355 | ENSDARP00000025541 | 0 | 0 | 0 | 0 | 0 | 0.071 | 0.900 | 0.138 | 0.912 |
| rpl13 | rpl7 | 1485678 | 1483164 | ENSDARP00000047390 | ENSDARP00000018980 | 0 | 0 | 0 | 0 | 0.997 | 0.976 | 0.900 | 0.649 | 0.999 |
| atp2a1l | ckmb | 1494243 | 1487353 | ENSDARP00000096674 | ENSDARP00000059365 | 0 | 0 | 0 | 0 | 0.955 | 0 | 0 | 0 | 0.956 |
| uqcrc1 | atp5a1 | 1499876 | 1484089 | ENSDARP00000108798 | ENSDARP00000027947 | 0 | 0 | 0 | 0 | 0.767 | 0.289 | 0 | 0.690 | 0.945 |
| pdlim7 | ckma | 1485440 | 1484819 | ENSDARP00000044908 | ENSDARP00000037871 | 0 | 0 | 0 | 0 | 0.971 | 0 | 0 | 0.237 | 0.976 |
| rpl32 | rplp2 | 1487450 | 1483852 | ENSDARP00000060004 | ENSDARP00000025616 | 0 | 0 | 0 | 0 | 0.995 | 0.811 | 0 | 0.097 | 0.999 |
| rps26 | rps24 | 1497897 | 1493019 | ENSDARP00000105328 | ENSDARP00000091586 | 0 | 0 | 0 | 0 | 0.759 | 0.861 | 0.900 | 0.646 | 0.998 |
| eif4a3 | rpl5a | 1484026 | 1481770 | ENSDARP00000027276 | ENSDARP00000006085 | 0 | 0 | 0 | 0 | 0.426 | 0.291 | 0.900 | 0.118 | 0.959 |
| tpi1a | gpib | 1484521 | 1482688 | ENSDARP00000033907 | ENSDARP00000014578 | 0.078 | 0 | 0 | 0 | 0.941 | 0.162 | 0.800 | 0.587 | 0.995 |
| smyhc1 | tnnc1a | 1486922 | 1483843 | ENSDARP00000056852 | ENSDARP00000025541 | 0 | 0 | 0 | 0 | 0 | 0.200 | 0.900 | 0.491 | 0.955 |
| rps24 | ddost | 1493019 | 1486503 | ENSDARP00000091586 | ENSDARP00000054289 | 0 | 0 | 0 | 0 | 0.067 | 0 | 0.900 | 0 | 0.902 |
| rps16 | rpl14 | 1488590 | 1486227 | ENSDARP00000066897 | ENSDARP00000052528 | 0.068 | 0 | 0 | 0 | 0.997 | 0.875 | 0.900 | 0.610 | 0.999 |
| rps26l | eif4a3 | 1501604 | 1484026 | ENSDARP00000111782 | ENSDARP00000027276 | 0 | 0 | 0 | 0 | 0.262 | 0.111 | 0.900 | 0.071 | 0.930 |
| rpl19 | rpl6 | 1498085 | 1493092 | ENSDARP00000105649 | ENSDARP00000091899 | 0.135 | 0 | 0 | 0 | 0.990 | 0.969 | 0.900 | 0.197 | 0.999 |
| rbm8a | eif4eb | 1483952 | 1481876 | ENSDARP00000026575 | ENSDARP00000007117 | 0 | 0 | 0 | 0 | 0.074 | 0 | 0.900 | 0 | 0.903 |
| rpl19 | rps10 | 1498085 | 1485542 | ENSDARP00000105649 | ENSDARP00000045900 | 0 | 0 | 0 | 0 | 0.964 | 0 | 0.900 | 0.182 | 0.996 |
| rps10 | rbm8a | 1485542 | 1483952 | ENSDARP00000045900 | ENSDARP00000026575 | 0 | 0 | 0 | 0 | 0.096 | 0 | 0.900 | 0 | 0.905 |
| rps26l | rpl23 | 1501604 | 1489208 | ENSDARP00000111782 | ENSDARP00000069977 | 0 | 0 | 0 | 0 | 0.997 | 0.405 | 0.900 | 0.087 | 0.999 |
| actn2 | desma | 1494007 | 1490431 | ENSDARP00000095652 | ENSDARP00000075994 | 0 | 0 | 0 | 0 | 0 | 0.084 | 0.900 | 0.072 | 0.907 |
| pkmb | MDH2 | 1505987 | 1488058 | ENSDARP00000122764 | ENSDARP00000063661 | 0.074 | 0 | 0 | 0 | 0.765 | 0.299 | 0.598 | 0.456 | 0.960 |
| rpl19 | rps24 | 1498085 | 1493019 | ENSDARP00000105649 | ENSDARP00000091586 | 0 | 0 | 0 | 0 | 0.895 | 0.817 | 0.900 | 0.144 | 0.998 |
| spcs1 | rps23 | 1490634 | 1484618 | ENSDARP00000076814 | ENSDARP00000035273 | 0 | 0 | 0 | 0 | 0.191 | 0 | 0.900 | 0 | 0.917 |
| smyhc1 | tnni2a.1 | 1486922 | 1484340 | ENSDARP00000056852 | ENSDARP00000031650 | 0 | 0 | 0 | 0 | 0.117 | 0.188 | 0.900 | 0.513 | 0.960 |
| spcs1 | rps27.1 | 1490634 | 1484170 | ENSDARP00000076814 | ENSDARP00000029079 | 0 | 0 | 0 | 0 | 0.225 | 0 | 0.900 | 0 | 0.921 |
| actc1b | tpm3 | 1486630 | 1481594 | ENSDARP00000055135 | ENSDARP00000004352 | 0 | 0 | 0 | 0 | 0.886 | 0.360 | 0 | 0.453 | 0.957 |
| rps26l | spcs1 | 1501604 | 1490634 | ENSDARP00000111782 | ENSDARP00000076814 | 0 | 0 | 0 | 0 | 0.250 | 0 | 0.900 | 0 | 0.921 |
| rps27.2 | nudc | 1489628 | 1481667 | ENSDARP00000072300 | ENSDARP00000005181 | 0 | 0 | 0 | 0 | 0 | 0 | 0.900 | 0 | 0.900 |
| rbm8a | rpl35 | 1483952 | 1483123 | ENSDARP00000026575 | ENSDARP00000018594 | 0 | 0 | 0 | 0 | 0.121 | 0 | 0.900 | 0 | 0.908 |
| actc1b | myl1 | 1486630 | 1481643 | ENSDARP00000055135 | ENSDARP00000004932 | 0 | 0 | 0 | 0 | 0.980 | 0.180 | 0 | 0.231 | 0.986 |
| rpl32 | ddost | 1487450 | 1486503 | ENSDARP00000060004 | ENSDARP00000054289 | 0 | 0 | 0 | 0 | 0.065 | 0 | 0.900 | 0 | 0.902 |
| EIF3F | rps27.1 | 1495369 | 1484170 | ENSDARP00000099664 | ENSDARP00000029079 | 0 | 0 | 0 | 0 | 0.358 | 0.070 | 0.900 | 0 | 0.936 |
| eef1a1l1 | rps3a | 1501580 | 1486104 | ENSDARP00000111742 | ENSDARP00000051762 | 0 | 0 | 0 | 0 | 0.886 | 0.149 | 0 | 0.374 | 0.936 |
| rps16 | rpl35 | 1488590 | 1483123 | ENSDARP00000066897 | ENSDARP00000018594 | 0.098 | 0 | 0 | 0 | 0.999 | 0.457 | 0.900 | 0.330 | 0.999 |
| atp5c1 | MDH2 | 1488594 | 1488058 | ENSDARP00000066929 | ENSDARP00000063661 | 0 | 0 | 0 | 0 | 0.969 | 0.073 | 0 | 0.241 | 0.977 |
| tnnt1 | tnnc1a | 1485382 | 1483843 | ENSDARP00000044153 | ENSDARP00000025541 | 0 | 0 | 0 | 0 | 0.086 | 0.847 | 0 | 0.967 | 0.995 |
| desma | MYL3 | 1490431 | 1488531 | ENSDARP00000075994 | ENSDARP00000066500 | 0 | 0 | 0 | 0 | 0 | 0.113 | 0.900 | 0 | 0.907 |
| rps3 | ddost | 1488779 | 1486503 | ENSDARP00000067802 | ENSDARP00000054289 | 0 | 0 | 0 | 0 | 0.083 | 0 | 0.900 | 0 | 0.904 |
| rps3a | eef2l2 | 1486104 | 1486009 | ENSDARP00000051762 | ENSDARP00000051080 | 0 | 0 | 0 | 0 | 0.941 | 0.860 | 0 | 0.086 | 0.991 |
| eif2s1 | rpl3 | 1488921 | 1481524 | ENSDARP00000068470 | ENSDARP00000003700 | 0 | 0 | 0 | 0 | 0.157 | 0 | 0.900 | 0 | 0.912 |
| rpl32 | rbm8a | 1487450 | 1483952 | ENSDARP00000060004 | ENSDARP00000026575 | 0 | 0 | 0 | 0 | 0.103 | 0 | 0.900 | 0 | 0.906 |
| spcs1 | rpl11 | 1490634 | 1488101 | ENSDARP00000076814 | ENSDARP00000063869 | 0 | 0 | 0 | 0 | 0.225 | 0 | 0.900 | 0 | 0.921 |
| eef1a1a | rpl36a | 1497414 | 1490292 | ENSDARP00000104468 | ENSDARP00000075363 | 0 | 0 | 0 | 0 | 0.169 | 0 | 0.900 | 0.064 | 0.915 |
| rpl19 | rps23 | 1498085 | 1484618 | ENSDARP00000105649 | ENSDARP00000035273 | 0 | 0 | 0 | 0 | 0.998 | 0.651 | 0.900 | 0.330 | 0.999 |
| eif4eb | rpl5a | 1481876 | 1481770 | ENSDARP00000007117 | ENSDARP00000006085 | 0 | 0 | 0 | 0 | 0.153 | 0 | 0.900 | 0.174 | 0.923 |
| rps24 | rpl28 | 1493019 | 1483702 | ENSDARP00000091586 | ENSDARP00000024189 | 0 | 0 | 0 | 0 | 0.633 | 0 | 0.900 | 0.065 | 0.963 |
| gapdhs | tpi1a | 1487169 | 1484521 | ENSDARP00000058383 | ENSDARP00000033907 | 0.493 | 0.083 | 0 | 0 | 0.608 | 0.320 | 0.957 | 0.518 | 0.996 |
| rbm8a | rpl28 | 1483952 | 1483702 | ENSDARP00000026575 | ENSDARP00000024189 | 0 | 0 | 0 | 0 | 0 | 0 | 0.900 | 0 | 0.900 |
| gapdhs | eno1a | 1487169 | 1481528 | ENSDARP00000058383 | ENSDARP00000003738 | 0.493 | 0 | 0 | 0 | 0.994 | 0.475 | 0 | 0.613 | 0.999 |
| rpl22 | rps16 | 1501438 | 1488590 | ENSDARP00000111487 | ENSDARP00000066897 | 0 | 0 | 0 | 0 | 0.957 | 0.753 | 0.900 | 0.208 | 0.999 |
| MYL3 | tnnc1a | 1488531 | 1483843 | ENSDARP00000066500 | ENSDARP00000025541 | 0 | 0 | 0 | 0.815 | 0 | 0 | 0.900 | 0 | 0.900 |
| rps24 | rps3 | 1493019 | 1488779 | ENSDARP00000091586 | ENSDARP00000067802 | 0 | 0 | 0 | 0 | 0.804 | 0.972 | 0.900 | 0.200 | 0.999 |
| actc1b | ckma | 1486630 | 1484819 | ENSDARP00000055135 | ENSDARP00000037871 | 0 | 0 | 0 | 0 | 0.979 | 0.111 | 0 | 0.097 | 0.981 |
| desma | pdlim7 | 1490431 | 1485440 | ENSDARP00000075994 | ENSDARP00000044908 | 0 | 0 | 0 | 0 | 0.934 | 0 | 0 | 0.160 | 0.942 |
| rpl28 | rpl5a | 1483702 | 1481770 | ENSDARP00000024189 | ENSDARP00000006085 | 0 | 0 | 0 | 0 | 0.998 | 0.849 | 0.900 | 0.654 | 0.999 |
| actc1a | tpm3 | 1495696 | 1481594 | ENSDARP00000100434 | ENSDARP00000004352 | 0 | 0 | 0 | 0 | 0.849 | 0.360 | 0 | 0.453 | 0.943 |
| rpsa | ddost | 1506145 | 1486503 | ENSDARP00000123183 | ENSDARP00000054289 | 0 | 0 | 0 | 0 | 0.096 | 0 | 0.900 | 0 | 0.905 |
| spcs1 | rps16 | 1490634 | 1488590 | ENSDARP00000076814 | ENSDARP00000066897 | 0 | 0 | 0 | 0 | 0.334 | 0 | 0.900 | 0 | 0.930 |
| cox5b2 | cox7c | 1497829 | 1489195 | ENSDARP00000105206 | ENSDARP00000069896 | 0 | 0 | 0 | 0 | 0.960 | 0.295 | 0.381 | 0.925 | 0.998 |
| rps27.2 | rps3a | 1489628 | 1486104 | ENSDARP00000072300 | ENSDARP00000051762 | 0 | 0 | 0 | 0 | 0.995 | 0.801 | 0.900 | 0.364 | 0.999 |
| rpl23 | rpl5a | 1489208 | 1481770 | ENSDARP00000069977 | ENSDARP00000006085 | 0.493 | 0 | 0 | 0 | 0.999 | 0.876 | 0.953 | 0.464 | 0.999 |
| rplp2 | rpl7 | 1483852 | 1483164 | ENSDARP00000025616 | ENSDARP00000018980 | 0 | 0 | 0 | 0 | 0.997 | 0.797 | 0 | 0.174 | 0.999 |
| eif2s1 | rpl32 | 1488921 | 1487450 | ENSDARP00000068470 | ENSDARP00000060004 | 0 | 0 | 0 | 0 | 0.104 | 0 | 0.900 | 0 | 0.906 |
| rpl22 | eif4eb | 1501438 | 1481876 | ENSDARP00000111487 | ENSDARP00000007117 | 0 | 0 | 0 | 0 | 0.149 | 0 | 0.900 | 0 | 0.911 |
| pvalb1 | tpma | 1486619 | 1484960 | ENSDARP00000055061 | ENSDARP00000039656 | 0 | 0 | 0 | 0 | 0.829 | 0.127 | 0 | 0.421 | 0.908 |
| rpl22 | rpl7 | 1501438 | 1483164 | ENSDARP00000111487 | ENSDARP00000018980 | 0 | 0 | 0 | 0 | 0.850 | 0.975 | 0.900 | 0.491 | 0.999 |
| rpsa | rpl36a | 1506145 | 1490292 | ENSDARP00000123183 | ENSDARP00000075363 | 0 | 0 | 0 | 0 | 0.997 | 0 | 0.900 | 0.147 | 0.999 |
| eno2 | aldocb | 1484405 | 1483738 | ENSDARP00000032456 | ENSDARP00000024492 | 0 | 0 | 0 | 0 | 0.750 | 0.070 | 0.800 | 0.459 | 0.971 |
| btf3 | rps23 | 1504780 | 1484618 | ENSDARP00000119683 | ENSDARP00000035273 | 0 | 0 | 0 | 0 | 0.974 | 0 | 0 | 0.069 | 0.976 |
| ddost | rpl3 | 1486503 | 1481524 | ENSDARP00000054289 | ENSDARP00000003700 | 0 | 0 | 0 | 0 | 0.136 | 0 | 0.900 | 0 | 0.909 |
| rps16 | rpl5a | 1488590 | 1481770 | ENSDARP00000066897 | ENSDARP00000006085 | 0.256 | 0 | 0 | 0 | 0.999 | 0.652 | 0.900 | 0.617 | 0.999 |
| eif2s1 | eif4eb | 1488921 | 1481876 | ENSDARP00000068470 | ENSDARP00000007117 | 0 | 0 | 0 | 0 | 0.157 | 0.110 | 0.964 | 0.789 | 0.993 |
| rpl11 | rpl5a | 1488101 | 1481770 | ENSDARP00000063869 | ENSDARP00000006085 | 0.493 | 0 | 0 | 0 | 0.999 | 0.877 | 0.953 | 0.944 | 0.999 |
| atp2a1l | tnnc2 | 1494243 | 1493861 | ENSDARP00000096674 | ENSDARP00000095111 | 0 | 0 | 0 | 0 | 0.883 | 0.079 | 0 | 0.362 | 0.927 |
| rps16 | rpl13 | 1488590 | 1485678 | ENSDARP00000066897 | ENSDARP00000047390 | 0 | 0 | 0 | 0 | 0.999 | 0.875 | 0.900 | 0.143 | 0.999 |
| rpl23 | rpl35 | 1489208 | 1483123 | ENSDARP00000069977 | ENSDARP00000018594 | 0.493 | 0 | 0 | 0 | 0.999 | 0.966 | 0.900 | 0.330 | 0.999 |
| rps3a | eif4eb | 1486104 | 1481876 | ENSDARP00000051762 | ENSDARP00000007117 | 0 | 0 | 0 | 0 | 0.142 | 0 | 0.900 | 0.071 | 0.913 |
| eif4ebp3l | eif4eb | 1487620 | 1481876 | ENSDARP00000060989 | ENSDARP00000007117 | 0 | 0 | 0 | 0 | 0 | 0.859 | 0.527 | 0.738 | 0.981 |
| vbp1 | tcp1 | 1501624 | 1497474 | ENSDARP00000111810 | ENSDARP00000104575 | 0 | 0 | 0 | 0 | 0.188 | 0.459 | 0.900 | 0.233 | 0.962 |
| tnnt3b | tnnc2 | 1497962 | 1493861 | ENSDARP00000105443 | ENSDARP00000095111 | 0 | 0 | 0 | 0 | 0.984 | 0.780 | 0.900 | 0.580 | 0.999 |
| EIF3F | rpl5a | 1495369 | 1481770 | ENSDARP00000099664 | ENSDARP00000006085 | 0 | 0 | 0 | 0 | 0.851 | 0.071 | 0.900 | 0 | 0.985 |
| rpl18a | rpl35 | 1484877 | 1483123 | ENSDARP00000038658 | ENSDARP00000018594 | 0 | 0 | 0 | 0 | 0.994 | 0.957 | 0.900 | 0.118 | 0.999 |
| rps10 | rpl18a | 1485542 | 1484877 | ENSDARP00000045900 | ENSDARP00000038658 | 0 | 0 | 0 | 0 | 0.986 | 0 | 0.900 | 0.137 | 0.998 |
| pdlim7 | mylz3 | 1485440 | 1483092 | ENSDARP00000044908 | ENSDARP00000018197 | 0 | 0 | 0 | 0 | 0.935 | 0.083 | 0 | 0.297 | 0.954 |
| psma1 | psmb3 | 1497161 | 1482604 | ENSDARP00000103895 | ENSDARP00000013624 | 0 | 0 | 0 | 0 | 0.977 | 0.978 | 0.964 | 0.613 | 0.999 |
| spcs1 | rps10 | 1490634 | 1485542 | ENSDARP00000076814 | ENSDARP00000045900 | 0 | 0 | 0 | 0 | 0.167 | 0 | 0.900 | 0 | 0.913 |
| rps27.2 | rpl28 | 1489628 | 1483702 | ENSDARP00000072300 | ENSDARP00000024189 | 0 | 0 | 0 | 0 | 0.994 | 0 | 0.900 | 0.118 | 0.999 |
| ATP5B | atp5l | 1487508 | 1481947 | ENSDARP00000060309 | ENSDARP00000007716 | 0 | 0 | 0 | 0 | 0.158 | 0.897 | 0.931 | 0.361 | 0.995 |
| rps24 | rps23 | 1493019 | 1484618 | ENSDARP00000091586 | ENSDARP00000035273 | 0 | 0 | 0 | 0 | 0.946 | 0.977 | 0.900 | 0.569 | 0.999 |
| rpsa | rps3a | 1506145 | 1486104 | ENSDARP00000123183 | ENSDARP00000051762 | 0.099 | 0 | 0 | 0 | 0.999 | 0.977 | 0.900 | 0.464 | 0.999 |
| psma1 | EIF3F | 1497161 | 1495369 | ENSDARP00000103895 | ENSDARP00000099664 | 0 | 0 | 0 | 0 | 0.900 | 0.323 | 0 | 0.201 | 0.942 |
| tnnt3b | actc1b | 1497962 | 1486630 | ENSDARP00000105443 | ENSDARP00000055135 | 0 | 0 | 0 | 0 | 0.912 | 0.096 | 0 | 0.148 | 0.928 |
| rpl7 | rpl5a | 1483164 | 1481770 | ENSDARP00000018980 | ENSDARP00000006085 | 0.493 | 0 | 0 | 0 | 0.999 | 0.875 | 0.900 | 0.492 | 0.999 |
| rps3 | rpl11 | 1488779 | 1488101 | ENSDARP00000067802 | ENSDARP00000063869 | 0.493 | 0 | 0 | 0 | 0.999 | 0.876 | 0.900 | 0.728 | 0.999 |
| pdlim7 | aldoab | 1485440 | 1485195 | ENSDARP00000044908 | ENSDARP00000042199 | 0 | 0 | 0 | 0 | 0.936 | 0 | 0 | 0 | 0.937 |
| eef1a1l1 | rps3 | 1501580 | 1488779 | ENSDARP00000111742 | ENSDARP00000067802 | 0 | 0 | 0 | 0 | 0.954 | 0.174 | 0 | 0.119 | 0.964 |
| actn2 | tnnc1a | 1494007 | 1483843 | ENSDARP00000095652 | ENSDARP00000025541 | 0 | 0 | 0 | 0 | 0 | 0.079 | 0.900 | 0.121 | 0.911 |
| rpl6 | rpl13 | 1493092 | 1485678 | ENSDARP00000091899 | ENSDARP00000047390 | 0 | 0 | 0 | 0 | 0.999 | 0.973 | 0.900 | 0.238 | 0.999 |
| actc1b | pvalb1 | 1486630 | 1486619 | ENSDARP00000055135 | ENSDARP00000055061 | 0 | 0 | 0 | 0 | 0.783 | 0.245 | 0 | 0.493 | 0.909 |
| fh | MDH2 | 1494425 | 1488058 | ENSDARP00000097494 | ENSDARP00000063661 | 0.242 | 0 | 0 | 0 | 0.537 | 0.449 | 0.957 | 0.584 | 0.996 |
| ckmb | mylz3 | 1487353 | 1483092 | ENSDARP00000059365 | ENSDARP00000018197 | 0 | 0 | 0 | 0 | 0.983 | 0.071 | 0 | 0 | 0.983 |
| rpl36a | rbm8a | 1490292 | 1483952 | ENSDARP00000075363 | ENSDARP00000026575 | 0 | 0 | 0 | 0 | 0.149 | 0 | 0.900 | 0 | 0.913 |
| rps10 | rplp2 | 1485542 | 1483852 | ENSDARP00000045900 | ENSDARP00000025616 | 0 | 0 | 0 | 0 | 0.994 | 0.285 | 0 | 0.151 | 0.996 |
| ckma | myl1 | 1484819 | 1481643 | ENSDARP00000037871 | ENSDARP00000004932 | 0 | 0 | 0 | 0 | 0.983 | 0.071 | 0 | 0.118 | 0.985 |
| eif4a3 | rpl28 | 1484026 | 1483702 | ENSDARP00000027276 | ENSDARP00000024189 | 0 | 0 | 0 | 0 | 0.142 | 0.191 | 0.900 | 0 | 0.926 |
| actc1b | aldoab | 1486630 | 1485195 | ENSDARP00000055135 | ENSDARP00000042199 | 0 | 0 | 0 | 0 | 0.968 | 0.079 | 0 | 0.218 | 0.975 |
| EIF3F | rps27.2 | 1495369 | 1489628 | ENSDARP00000099664 | ENSDARP00000072300 | 0 | 0 | 0 | 0 | 0.221 | 0.070 | 0.900 | 0 | 0.923 |
| rpl18a | rpl5a | 1484877 | 1481770 | ENSDARP00000038658 | ENSDARP00000006085 | 0 | 0 | 0 | 0 | 0.998 | 0.874 | 0.900 | 0.180 | 0.999 |
| rps24 | rpl3 | 1493019 | 1481524 | ENSDARP00000091586 | ENSDARP00000003700 | 0 | 0 | 0 | 0 | 0.543 | 0.810 | 0.900 | 0.088 | 0.991 |
| rps26 | EIF3F | 1497897 | 1495369 | ENSDARP00000105328 | ENSDARP00000099664 | 0 | 0 | 0 | 0 | 0.793 | 0 | 0.900 | 0 | 0.978 |
| rps29 | eef2l2 | 1487533 | 1486009 | ENSDARP00000060443 | ENSDARP00000051080 | 0.493 | 0 | 0 | 0 | 0.761 | 0.837 | 0 | 0.255 | 0.983 |
| rps27.2 | rpl32 | 1489628 | 1487450 | ENSDARP00000072300 | ENSDARP00000060004 | 0 | 0 | 0 | 0 | 0.996 | 0 | 0.900 | 0.330 | 0.999 |
| rpl14 | rpl18a | 1486227 | 1484877 | ENSDARP00000052528 | ENSDARP00000038658 | 0 | 0 | 0 | 0 | 0.994 | 0.968 | 0.900 | 0.362 | 0.999 |
| rps26l | rpl7 | 1501604 | 1483164 | ENSDARP00000111782 | ENSDARP00000018980 | 0 | 0 | 0 | 0 | 0.941 | 0.298 | 0.900 | 0.110 | 0.995 |
| tnnt3b | tnnc1a | 1497962 | 1483843 | ENSDARP00000105443 | ENSDARP00000025541 | 0 | 0 | 0 | 0 | 0.100 | 0.255 | 0.900 | 0.192 | 0.938 |
| LOC567740 | desma | 1505894 | 1490431 | ENSDARP00000122502 | ENSDARP00000075994 | 0 | 0 | 0 | 0 | 0 | 0 | 0.900 | 0.064 | 0.902 |
| gapdh | gpib | 1488083 | 1482688 | ENSDARP00000063799 | ENSDARP00000014578 | 0.142 | 0 | 0 | 0 | 0.275 | 0 | 0.800 | 0.499 | 0.930 |
| EIF3F | rps3 | 1495369 | 1488779 | ENSDARP00000099664 | ENSDARP00000067802 | 0 | 0 | 0 | 0 | 0.637 | 0 | 0.900 | 0 | 0.963 |
| rps29 | eif4a3 | 1487533 | 1484026 | ENSDARP00000060443 | ENSDARP00000027276 | 0 | 0 | 0 | 0 | 0.086 | 0.081 | 0.900 | 0.174 | 0.921 |
| rps27.2 | eif4eb | 1489628 | 1481876 | ENSDARP00000072300 | ENSDARP00000007117 | 0 | 0 | 0 | 0 | 0.117 | 0 | 0.900 | 0 | 0.907 |
| rpl6 | rps27.2 | 1493092 | 1489628 | ENSDARP00000091899 | ENSDARP00000072300 | 0 | 0 | 0 | 0 | 0.964 | 0.395 | 0.900 | 0.112 | 0.997 |
| rpl28 | rpl7 | 1483702 | 1483164 | ENSDARP00000024189 | ENSDARP00000018980 | 0 | 0 | 0 | 0 | 0.924 | 0.957 | 0.900 | 0.204 | 0.999 |
| actc1a | smyhc1 | 1495696 | 1486922 | ENSDARP00000100434 | ENSDARP00000056852 | 0 | 0 | 0 | 0 | 0.794 | 0.294 | 0 | 0.492 | 0.921 |
| tnnc2 | myl10 | 1493861 | 1485924 | ENSDARP00000095111 | ENSDARP00000050204 | 0 | 0 | 0 | 0 | 0.971 | 0.080 | 0 | 0.114 | 0.974 |
| pgk1 | gapdhs | 1489379 | 1487169 | ENSDARP00000070807 | ENSDARP00000058383 | 0.493 | 0 | 0 | 0 | 0.803 | 0.806 | 0.957 | 0.615 | 0.999 |
| aldoab | eno1a | 1485195 | 1481528 | ENSDARP00000042199 | ENSDARP00000003738 | 0 | 0 | 0 | 0 | 0.763 | 0.070 | 0.800 | 0.459 | 0.973 |
| rps23 | rpl5a | 1484618 | 1481770 | ENSDARP00000035273 | ENSDARP00000006085 | 0.493 | 0 | 0 | 0 | 0.996 | 0.322 | 0.900 | 0.537 | 0.999 |
| rps23 | rplp2 | 1484618 | 1483852 | ENSDARP00000035273 | ENSDARP00000025616 | 0 | 0 | 0 | 0 | 0.998 | 0.595 | 0 | 0.087 | 0.999 |
| rps26l | rpl3 | 1501604 | 1481524 | ENSDARP00000111782 | ENSDARP00000003700 | 0 | 0 | 0 | 0 | 0.973 | 0.308 | 0.900 | 0.382 | 0.998 |
| EIF3F | rpl18a | 1495369 | 1484877 | ENSDARP00000099664 | ENSDARP00000038658 | 0 | 0 | 0 | 0 | 0.724 | 0 | 0.900 | 0.148 | 0.975 |
| eif2s1 | rps3 | 1488921 | 1488779 | ENSDARP00000068470 | ENSDARP00000067802 | 0 | 0 | 0 | 0 | 0.116 | 0 | 0.900 | 0.504 | 0.953 |
| eno3 | aldoaa | 1505202 | 1504686 | ENSDARP00000120742 | ENSDARP00000119413 | 0 | 0 | 0 | 0 | 0.750 | 0.070 | 0.800 | 0.624 | 0.980 |
| rps26 | rpl13 | 1497897 | 1485678 | ENSDARP00000105328 | ENSDARP00000047390 | 0 | 0 | 0 | 0 | 0.998 | 0 | 0.900 | 0.470 | 0.999 |
| rpsa | rplp2 | 1506145 | 1483852 | ENSDARP00000123183 | ENSDARP00000025616 | 0 | 0 | 0 | 0 | 0.998 | 0.633 | 0 | 0.115 | 0.999 |
| utp18 | mphosph10 | 1495391 | 1483869 | ENSDARP00000099725 | ENSDARP00000025759 | 0 | 0 | 0 | 0 | 0.714 | 0.447 | 0 | 0.713 | 0.951 |
| pgk1 | eno1a | 1489379 | 1481528 | ENSDARP00000070807 | ENSDARP00000003738 | 0.493 | 0 | 0 | 0 | 0.976 | 0.638 | 0 | 0.681 | 0.998 |
| ldha | gpib | 1487429 | 1482688 | ENSDARP00000059885 | ENSDARP00000014578 | 0.118 | 0 | 0 | 0 | 0.115 | 0 | 0.800 | 0.478 | 0.907 |
| ndufa12 | ndufs4 | 1487803 | 1486007 | ENSDARP00000062277 | ENSDARP00000051054 | 0 | 0 | 0.262 | 0 | 0.758 | 0.700 | 0.934 | 0.231 | 0.996 |
| rpl13 | rps27.1 | 1485678 | 1484170 | ENSDARP00000047390 | ENSDARP00000029079 | 0 | 0 | 0 | 0 | 0.996 | 0.454 | 0.900 | 0.234 | 0.999 |
| rpl22 | ddost | 1501438 | 1486503 | ENSDARP00000111487 | ENSDARP00000054289 | 0 | 0 | 0 | 0 | 0.079 | 0 | 0.900 | 0 | 0.903 |
| tnnt3b | desmb | 1497962 | 1488347 | ENSDARP00000105443 | ENSDARP00000065355 | 0 | 0 | 0 | 0 | 0.066 | 0 | 0.900 | 0 | 0.902 |
| rpl36a | eif4eb | 1490292 | 1481876 | ENSDARP00000075363 | ENSDARP00000007117 | 0 | 0 | 0 | 0 | 0.274 | 0 | 0.900 | 0 | 0.924 |
| rps29 | rpl13 | 1487533 | 1485678 | ENSDARP00000060443 | ENSDARP00000047390 | 0 | 0 | 0 | 0 | 0.992 | 0 | 0.900 | 0.620 | 0.999 |
| myl1 | tpm3 | 1481643 | 1481594 | ENSDARP00000004932 | ENSDARP00000004352 | 0 | 0 | 0 | 0 | 0.910 | 0.134 | 0.900 | 0.087 | 0.992 |
| rps27.2 | rps10 | 1489628 | 1485542 | ENSDARP00000072300 | ENSDARP00000045900 | 0 | 0 | 0 | 0 | 0.766 | 0.792 | 0.900 | 0.195 | 0.995 |
| myl2 | tnni2a.4 | 1503455 | 1484806 | ENSDARP00000116241 | ENSDARP00000037759 | 0 | 0 | 0 | 0 | 0.167 | 0.187 | 0.900 | 0.488 | 0.961 |
| pgk1 | aldoab | 1489379 | 1485195 | ENSDARP00000070807 | ENSDARP00000042199 | 0.378 | 0 | 0 | 0 | 0.478 | 0.177 | 0.408 | 0.503 | 0.907 |
| rplp2 | rpl35 | 1483852 | 1483123 | ENSDARP00000025616 | ENSDARP00000018594 | 0 | 0 | 0 | 0 | 0.995 | 0.797 | 0 | 0.365 | 0.999 |
| MYL3 | tnni2a.4 | 1488531 | 1484806 | ENSDARP00000066500 | ENSDARP00000037759 | 0 | 0 | 0 | 0 | 0.099 | 0.187 | 0.900 | 0.181 | 0.931 |
| rps24 | rps3a | 1493019 | 1486104 | ENSDARP00000091586 | ENSDARP00000051762 | 0.068 | 0 | 0 | 0 | 0.785 | 0.976 | 0.900 | 0.243 | 0.999 |
| ldha | aldoab | 1487429 | 1485195 | ENSDARP00000059885 | ENSDARP00000042199 | 0 | 0 | 0 | 0 | 0.290 | 0 | 0.800 | 0.364 | 0.904 |
| eno3 | tpi1a | 1505202 | 1484521 | ENSDARP00000120742 | ENSDARP00000033907 | 0.493 | 0 | 0 | 0 | 0.941 | 0.286 | 0 | 0.620 | 0.991 |
| rpl19 | rpl28 | 1498085 | 1483702 | ENSDARP00000105649 | ENSDARP00000024189 | 0 | 0 | 0 | 0 | 0.997 | 0.956 | 0.900 | 0.118 | 0.999 |
| psma3 | pomp | 1499751 | 1485465 | ENSDARP00000108560 | ENSDARP00000045123 | 0 | 0 | 0 | 0 | 0.280 | 0.940 | 0 | 0.351 | 0.969 |
| tnnt3b | MYL3 | 1497962 | 1488531 | ENSDARP00000105443 | ENSDARP00000066500 | 0 | 0 | 0 | 0 | 0.098 | 0.176 | 0.900 | 0.095 | 0.923 |
| rps16 | rps3a | 1488590 | 1486104 | ENSDARP00000066897 | ENSDARP00000051762 | 0 | 0 | 0 | 0 | 0.999 | 0.978 | 0.900 | 0.438 | 0.999 |
| rpl36a | rpl7 | 1490292 | 1483164 | ENSDARP00000075363 | ENSDARP00000018980 | 0 | 0 | 0 | 0 | 0.986 | 0.962 | 0.900 | 0.369 | 0.999 |
| tnnt1 | tnni2a.4 | 1485382 | 1484806 | ENSDARP00000044153 | ENSDARP00000037759 | 0 | 0 | 0 | 0 | 0.993 | 0.324 | 0 | 0.765 | 0.998 |
| rps24 | rpl35 | 1493019 | 1483123 | ENSDARP00000091586 | ENSDARP00000018594 | 0 | 0 | 0 | 0 | 0.980 | 0.863 | 0.900 | 0.218 | 0.999 |
| rps27.2 | rbm8a | 1489628 | 1483952 | ENSDARP00000072300 | ENSDARP00000026575 | 0 | 0 | 0 | 0 | 0.097 | 0 | 0.900 | 0 | 0.905 |
| spcs1 | rpl18a | 1490634 | 1484877 | ENSDARP00000076814 | ENSDARP00000038658 | 0 | 0 | 0 | 0 | 0.312 | 0 | 0.900 | 0 | 0.929 |
| rpl11 | rps29 | 1488101 | 1487533 | ENSDARP00000063869 | ENSDARP00000060443 | 0.493 | 0 | 0 | 0 | 0.999 | 0 | 0.900 | 0.402 | 0.999 |
| rps23 | rpl35 | 1484618 | 1483123 | ENSDARP00000035273 | ENSDARP00000018594 | 0.493 | 0 | 0 | 0 | 0.998 | 0.633 | 0.900 | 0.330 | 0.999 |
| rpl36a | rpl18a | 1490292 | 1484877 | ENSDARP00000075363 | ENSDARP00000038658 | 0 | 0 | 0 | 0 | 0.998 | 0.958 | 0.900 | 0.106 | 0.999 |
| eef2l2 | rpl13 | 1486009 | 1485678 | ENSDARP00000051080 | ENSDARP00000047390 | 0 | 0 | 0 | 0 | 0.759 | 0.633 | 0 | 0 | 0.907 |
| rps10 | rps27.1 | 1485542 | 1484170 | ENSDARP00000045900 | ENSDARP00000029079 | 0 | 0 | 0 | 0 | 0.858 | 0.792 | 0.900 | 0.577 | 0.998 |
| rpl23 | eif4eb | 1489208 | 1481876 | ENSDARP00000069977 | ENSDARP00000007117 | 0 | 0 | 0 | 0 | 0.104 | 0 | 0.900 | 0 | 0.906 |
| rock2a | myl2 | 1505939 | 1503455 | ENSDARP00000122621 | ENSDARP00000116241 | 0 | 0 | 0 | 0 | 0 | 0.112 | 0.903 | 0.565 | 0.961 |
| rps27.2 | rpl13 | 1489628 | 1485678 | ENSDARP00000072300 | ENSDARP00000047390 | 0 | 0 | 0 | 0 | 0.965 | 0.454 | 0.900 | 0.234 | 0.998 |
| ATP5B | atp5a1 | 1487508 | 1484089 | ENSDARP00000060309 | ENSDARP00000027947 | 0.493 | 0 | 0.520 | 0.633 | 0.995 | 0.978 | 0.937 | 0.922 | 0.999 |
| MDH2 | sdhb | 1488058 | 1487800 | ENSDARP00000063661 | ENSDARP00000062263 | 0.257 | 0 | 0 | 0 | 0.835 | 0 | 0 | 0.375 | 0.918 |
| rps26 | spcs1 | 1497897 | 1490634 | ENSDARP00000105328 | ENSDARP00000076814 | 0 | 0 | 0 | 0 | 0.278 | 0 | 0.900 | 0 | 0.924 |
| rpl6 | rpl7 | 1493092 | 1483164 | ENSDARP00000091899 | ENSDARP00000018980 | 0.190 | 0 | 0 | 0 | 0.970 | 0.973 | 0.900 | 0.568 | 0.999 |
| rps29 | rbm8a | 1487533 | 1483952 | ENSDARP00000060443 | ENSDARP00000026575 | 0 | 0 | 0 | 0 | 0.098 | 0 | 0.900 | 0 | 0.905 |
| pkmb | ldha | 1505987 | 1487429 | ENSDARP00000122764 | ENSDARP00000059885 | 0.074 | 0 | 0 | 0 | 0.099 | 0.299 | 0.957 | 0.598 | 0.988 |
| actc1b | mylz3 | 1486630 | 1483092 | ENSDARP00000055135 | ENSDARP00000018197 | 0 | 0 | 0 | 0 | 0.969 | 0.180 | 0 | 0.570 | 0.988 |
| rpl23 | rpl11 | 1489208 | 1488101 | ENSDARP00000069977 | ENSDARP00000063869 | 0.493 | 0 | 0 | 0 | 0.999 | 0.978 | 0.953 | 0.364 | 0.999 |
| rpl6 | eif4a3 | 1493092 | 1484026 | ENSDARP00000091899 | ENSDARP00000027276 | 0 | 0 | 0 | 0 | 0.294 | 0.191 | 0.900 | 0 | 0.937 |
| spcs1 | rps3 | 1490634 | 1488779 | ENSDARP00000076814 | ENSDARP00000067802 | 0 | 0 | 0 | 0 | 0.126 | 0 | 0.900 | 0 | 0.910 |
| rps27.2 | rps29 | 1489628 | 1487533 | ENSDARP00000072300 | ENSDARP00000060443 | 0 | 0 | 0 | 0 | 0.997 | 0.797 | 0.900 | 0.234 | 0.999 |
| rpl32 | rpl7 | 1487450 | 1483164 | ENSDARP00000060004 | ENSDARP00000018980 | 0.493 | 0 | 0 | 0 | 0.962 | 0.975 | 0.900 | 0.245 | 0.999 |
| tpma | ckma | 1484960 | 1484819 | ENSDARP00000039656 | ENSDARP00000037871 | 0 | 0 | 0 | 0 | 0.972 | 0.072 | 0 | 0.198 | 0.978 |
| rps26l | rps29 | 1501604 | 1487533 | ENSDARP00000111782 | ENSDARP00000060443 | 0 | 0 | 0 | 0 | 0.992 | 0.861 | 0.900 | 0.491 | 0.999 |
| ddost | rpl14 | 1486503 | 1486227 | ENSDARP00000054289 | ENSDARP00000052528 | 0 | 0 | 0 | 0 | 0.066 | 0 | 0.900 | 0 | 0.902 |
| ckmb | tpma | 1487353 | 1484960 | ENSDARP00000059365 | ENSDARP00000039656 | 0 | 0 | 0 | 0 | 0.984 | 0.072 | 0 | 0.086 | 0.985 |
| me2 | fh | 1500379 | 1494425 | ENSDARP00000109682 | ENSDARP00000097494 | 0.257 | 0 | 0 | 0 | 0.081 | 0 | 0.946 | 0.361 | 0.974 |
| tnni2a.1 | tpm3 | 1484340 | 1481594 | ENSDARP00000031650 | ENSDARP00000004352 | 0 | 0 | 0 | 0 | 0.197 | 0.285 | 0.900 | 0.658 | 0.977 |
| pgk1 | gapdh | 1489379 | 1488083 | ENSDARP00000070807 | ENSDARP00000063799 | 0.493 | 0 | 0 | 0 | 0.690 | 0.806 | 0.957 | 0.616 | 0.999 |
| atp5a1 | atp5l | 1484089 | 1481947 | ENSDARP00000027947 | ENSDARP00000007716 | 0 | 0 | 0 | 0 | 0.106 | 0.897 | 0.931 | 0.218 | 0.994 |
| rpl19 | rbm8a | 1498085 | 1483952 | ENSDARP00000105649 | ENSDARP00000026575 | 0 | 0 | 0 | 0 | 0.096 | 0 | 0.900 | 0 | 0.905 |
| atp5d | atp5l | 1483512 | 1481947 | ENSDARP00000022528 | ENSDARP00000007716 | 0 | 0 | 0 | 0 | 0.975 | 0.365 | 0.931 | 0.364 | 0.999 |
| gapdhs | aldocb | 1487169 | 1483738 | ENSDARP00000058383 | ENSDARP00000024492 | 0.257 | 0 | 0 | 0 | 0.986 | 0.206 | 0.949 | 0.430 | 0.999 |
| rpl36a | rpl5a | 1490292 | 1481770 | ENSDARP00000075363 | ENSDARP00000006085 | 0 | 0 | 0 | 0 | 0.998 | 0.865 | 0.900 | 0.281 | 0.999 |
| rpl14 | rplp2 | 1486227 | 1483852 | ENSDARP00000052528 | ENSDARP00000025616 | 0 | 0 | 0 | 0 | 0.997 | 0.873 | 0 | 0.066 | 0.999 |
| rps29 | ddost | 1487533 | 1486503 | ENSDARP00000060443 | ENSDARP00000054289 | 0 | 0 | 0 | 0 | 0 | 0 | 0.900 | 0 | 0.900 |
| rpl22 | rpl36a | 1501438 | 1490292 | ENSDARP00000111487 | ENSDARP00000075363 | 0 | 0 | 0 | 0 | 0.989 | 0.958 | 0.900 | 0.202 | 0.999 |
| rps24 | rplp2 | 1493019 | 1483852 | ENSDARP00000091586 | ENSDARP00000025616 | 0 | 0 | 0 | 0 | 0.998 | 0.788 | 0 | 0.235 | 0.999 |
| arpc3 | ACTR3B | 1490230 | 1485468 | ENSDARP00000075074 | ENSDARP00000045149 | 0 | 0 | 0 | 0 | 0.066 | 0.880 | 0.570 | 0.614 | 0.978 |
| rps26l | rps23 | 1501604 | 1484618 | ENSDARP00000111782 | ENSDARP00000035273 | 0 | 0 | 0 | 0 | 0.971 | 0.861 | 0.900 | 0.496 | 0.999 |
| rps27.2 | rplp2 | 1489628 | 1483852 | ENSDARP00000072300 | ENSDARP00000025616 | 0 | 0 | 0 | 0 | 0.998 | 0.384 | 0 | 0.086 | 0.999 |
| atp5c1 | sdhb | 1488594 | 1487800 | ENSDARP00000066929 | ENSDARP00000062263 | 0 | 0 | 0 | 0 | 0.875 | 0 | 0 | 0.515 | 0.938 |
| rpsa | rpl7 | 1506145 | 1483164 | ENSDARP00000123183 | ENSDARP00000018980 | 0 | 0 | 0 | 0 | 0.992 | 0.294 | 0.900 | 0.330 | 0.999 |
| rps3a | rpl7 | 1486104 | 1483164 | ENSDARP00000051762 | ENSDARP00000018980 | 0 | 0 | 0 | 0 | 0.997 | 0.874 | 0.900 | 0.466 | 0.999 |
| rps16 | ddost | 1488590 | 1486503 | ENSDARP00000066897 | ENSDARP00000054289 | 0 | 0 | 0 | 0 | 0.117 | 0 | 0.900 | 0 | 0.907 |
| spcs1 | ddost | 1490634 | 1486503 | ENSDARP00000076814 | ENSDARP00000054289 | 0 | 0 | 0 | 0 | 0.168 | 0 | 0.900 | 0 | 0.915 |
| eif4a3 | eif4eb | 1484026 | 1481876 | ENSDARP00000027276 | ENSDARP00000007117 | 0 | 0 | 0 | 0 | 0.098 | 0.460 | 0.949 | 0.764 | 0.993 |
| rpl32 | rps23 | 1487450 | 1484618 | ENSDARP00000060004 | ENSDARP00000035273 | 0 | 0 | 0 | 0 | 0.993 | 0.924 | 0.900 | 0.330 | 0.999 |
| rps26 | rps27.1 | 1497897 | 1484170 | ENSDARP00000105328 | ENSDARP00000029079 | 0 | 0 | 0 | 0 | 0.999 | 0.678 | 0.900 | 0.380 | 0.999 |
| rps3 | rpl3 | 1488779 | 1481524 | ENSDARP00000067802 | ENSDARP00000003700 | 0.493 | 0 | 0 | 0 | 0.999 | 0.874 | 0.900 | 0.371 | 0.999 |
| LOC567740 | tnnt3b | 1505894 | 1497962 | ENSDARP00000122502 | ENSDARP00000105443 | 0 | 0 | 0 | 0 | 0.278 | 0.335 | 0.900 | 0.389 | 0.966 |
| spcs1 | rpl5a | 1490634 | 1481770 | ENSDARP00000076814 | ENSDARP00000006085 | 0 | 0 | 0 | 0 | 0.296 | 0 | 0.900 | 0 | 0.928 |
| eef2l2 | rpl35 | 1486009 | 1483123 | ENSDARP00000051080 | ENSDARP00000018594 | 0.493 | 0 | 0 | 0 | 0.697 | 0.633 | 0 | 0.234 | 0.951 |
| eef2l2 | rpl18a | 1486009 | 1484877 | ENSDARP00000051080 | ENSDARP00000038658 | 0 | 0 | 0 | 0 | 0.777 | 0.633 | 0 | 0.088 | 0.918 |
| atp5c1 | atp5l | 1488594 | 1481947 | ENSDARP00000066929 | ENSDARP00000007716 | 0 | 0 | 0 | 0 | 0.907 | 0 | 0.931 | 0.362 | 0.995 |
| rpl6 | rpl14 | 1493092 | 1486227 | ENSDARP00000091899 | ENSDARP00000052528 | 0 | 0 | 0 | 0 | 0.986 | 0.967 | 0.900 | 0.612 | 0.999 |
| rps29 | rplp2 | 1487533 | 1483852 | ENSDARP00000060443 | ENSDARP00000025616 | 0 | 0 | 0 | 0 | 0.994 | 0 | 0 | 0.623 | 0.997 |
| pgk1 | eno2 | 1489379 | 1484405 | ENSDARP00000070807 | ENSDARP00000032456 | 0.493 | 0 | 0.204 | 0 | 0.929 | 0.638 | 0 | 0.611 | 0.995 |
| snrpb | sf3b3 | 1483602 | 1483086 | ENSDARP00000023337 | ENSDARP00000018100 | 0 | 0 | 0 | 0 | 0.197 | 0.842 | 0 | 0.309 | 0.909 |
| tnnc2 | tpma | 1493861 | 1484960 | ENSDARP00000095111 | ENSDARP00000039656 | 0 | 0 | 0 | 0 | 0.983 | 0.127 | 0 | 0.658 | 0.994 |
| rps27.1 | rbm8a | 1484170 | 1483952 | ENSDARP00000029079 | ENSDARP00000026575 | 0 | 0 | 0 | 0 | 0.097 | 0 | 0.900 | 0 | 0.905 |
| rpl35 | eif4eb | 1483123 | 1481876 | ENSDARP00000018594 | ENSDARP00000007117 | 0 | 0 | 0 | 0 | 0.105 | 0 | 0.900 | 0 | 0.906 |
| tnni2a.4 | myl1 | 1484806 | 1481643 | ENSDARP00000037759 | ENSDARP00000004932 | 0 | 0 | 0 | 0 | 0.214 | 0.187 | 0.900 | 0.372 | 0.956 |
| myl2 | desma | 1503455 | 1490431 | ENSDARP00000116241 | ENSDARP00000075994 | 0 | 0 | 0 | 0 | 0 | 0.071 | 0.900 | 0 | 0.903 |
| MYL3 | myl1 | 1488531 | 1481643 | ENSDARP00000066500 | ENSDARP00000004932 | 0 | 0 | 0 | 0.948 | 0 | 0 | 0.900 | 0.236 | 0.901 |
| aldoaa | gpib | 1504686 | 1482688 | ENSDARP00000119413 | ENSDARP00000014578 | 0 | 0 | 0 | 0 | 0.691 | 0 | 0.800 | 0.460 | 0.963 |
| rpl23 | rpl32 | 1489208 | 1487450 | ENSDARP00000069977 | ENSDARP00000060004 | 0.493 | 0 | 0 | 0 | 0.996 | 0.975 | 0.900 | 0.330 | 0.999 |
| pkmb | me2 | 1505987 | 1500379 | ENSDARP00000122764 | ENSDARP00000109682 | 0.077 | 0 | 0 | 0 | 0.143 | 0 | 0.932 | 0.596 | 0.976 |
| LOC567740 | myl1 | 1505894 | 1481643 | ENSDARP00000122502 | ENSDARP00000004932 | 0 | 0 | 0 | 0 | 0.098 | 0.203 | 0.900 | 0.231 | 0.937 |
| rps3a | rps10 | 1486104 | 1485542 | ENSDARP00000051762 | ENSDARP00000045900 | 0 | 0 | 0 | 0 | 0.992 | 0.977 | 0.900 | 0.351 | 0.999 |
| rpl11 | rpl13 | 1488101 | 1485678 | ENSDARP00000063869 | ENSDARP00000047390 | 0 | 0 | 0 | 0 | 0.999 | 0.976 | 0.900 | 0.492 | 0.999 |
| rpl22 | rps3a | 1501438 | 1486104 | ENSDARP00000111487 | ENSDARP00000051762 | 0 | 0 | 0 | 0 | 0.934 | 0.748 | 0.900 | 0.152 | 0.998 |
| psma3 | smurf1 | 1499751 | 1481527 | ENSDARP00000108560 | ENSDARP00000003721 | 0 | 0 | 0 | 0 | 0 | 0.070 | 0.900 | 0 | 0.903 |
| rps27.2 | rps27.1 | 1489628 | 1484170 | ENSDARP00000072300 | ENSDARP00000029079 | 0 | 0 | 0.531 | 0.987 | 0.337 | 0 | 0.900 | 0 | 0.931 |
| rpl19 | rplp2 | 1498085 | 1483852 | ENSDARP00000105649 | ENSDARP00000025616 | 0 | 0 | 0 | 0 | 0.998 | 0.796 | 0 | 0.174 | 0.999 |
| EIF3F | rpl11 | 1495369 | 1488101 | ENSDARP00000099664 | ENSDARP00000063869 | 0 | 0 | 0 | 0 | 0.861 | 0 | 0.900 | 0 | 0.985 |
| rps3a | rpl18a | 1486104 | 1484877 | ENSDARP00000051762 | ENSDARP00000038658 | 0 | 0 | 0 | 0 | 0.999 | 0.875 | 0.900 | 0.201 | 0.999 |
| LOC567740 | tnnc1a | 1505894 | 1483843 | ENSDARP00000122502 | ENSDARP00000025541 | 0 | 0 | 0 | 0 | 0 | 0.200 | 0.900 | 0.491 | 0.955 |
| rpl6 | rps29 | 1493092 | 1487533 | ENSDARP00000091899 | ENSDARP00000060443 | 0.098 | 0 | 0 | 0 | 0.924 | 0.278 | 0.900 | 0.655 | 0.998 |
| rpl11 | rplp2 | 1488101 | 1483852 | ENSDARP00000063869 | ENSDARP00000025616 | 0 | 0 | 0 | 0 | 0.999 | 0.797 | 0 | 0.175 | 0.999 |
| eif2s1 | rps16 | 1488921 | 1488590 | ENSDARP00000068470 | ENSDARP00000066897 | 0 | 0 | 0 | 0 | 0.148 | 0 | 0.900 | 0 | 0.911 |
| tnnc2 | myl1 | 1493861 | 1481643 | ENSDARP00000095111 | ENSDARP00000004932 | 0 | 0 | 0 | 0.810 | 0.994 | 0 | 0.900 | 0.177 | 0.999 |
| myl2 | actn2 | 1503455 | 1494007 | ENSDARP00000116241 | ENSDARP00000095652 | 0 | 0 | 0 | 0 | 0.064 | 0.103 | 0.900 | 0.438 | 0.947 |
| LOC567740 | tnni2a.1 | 1505894 | 1484340 | ENSDARP00000122502 | ENSDARP00000031650 | 0 | 0 | 0 | 0 | 0.117 | 0.188 | 0.900 | 0.513 | 0.960 |
| rpl22 | rpl32 | 1501438 | 1487450 | ENSDARP00000111487 | ENSDARP00000060004 | 0 | 0 | 0 | 0 | 0.717 | 0.958 | 0.900 | 0.150 | 0.998 |
| rpl19 | rpl32 | 1498085 | 1487450 | ENSDARP00000105649 | ENSDARP00000060004 | 0.493 | 0 | 0 | 0 | 0.990 | 0.974 | 0.900 | 0.564 | 0.999 |
| psma1 | pomp | 1497161 | 1485465 | ENSDARP00000103895 | ENSDARP00000045123 | 0 | 0 | 0 | 0 | 0.702 | 0.939 | 0 | 0.491 | 0.990 |
| rps26l | rpl28 | 1501604 | 1483702 | ENSDARP00000111782 | ENSDARP00000024189 | 0 | 0 | 0 | 0 | 0.999 | 0 | 0.900 | 0.188 | 0.999 |
| smyhc1 | mylz3 | 1486922 | 1483092 | ENSDARP00000056852 | ENSDARP00000018197 | 0 | 0 | 0 | 0 | 0.449 | 0.202 | 0.900 | 0.209 | 0.960 |
| rpl19 | rpl7 | 1498085 | 1483164 | ENSDARP00000105649 | ENSDARP00000018980 | 0.493 | 0 | 0 | 0 | 0.986 | 0.978 | 0.900 | 0.489 | 0.999 |
| rps24 | spcs1 | 1493019 | 1490634 | ENSDARP00000091586 | ENSDARP00000076814 | 0 | 0 | 0 | 0 | 0.165 | 0 | 0.900 | 0 | 0.912 |
| psmb3 | smurf1 | 1482604 | 1481527 | ENSDARP00000013624 | ENSDARP00000003721 | 0 | 0 | 0 | 0 | 0 | 0.069 | 0.900 | 0 | 0.902 |
| me2 | MDH2 | 1500379 | 1488058 | ENSDARP00000109682 | ENSDARP00000063661 | 0.248 | 0 | 0 | 0 | 0.130 | 0.215 | 0.934 | 0.464 | 0.979 |
| ddost | rpl7 | 1486503 | 1483164 | ENSDARP00000054289 | ENSDARP00000018980 | 0 | 0 | 0 | 0 | 0.067 | 0 | 0.900 | 0 | 0.902 |
| atp5c1 | atp5d | 1488594 | 1483512 | ENSDARP00000066929 | ENSDARP00000022528 | 0.493 | 0 | 0 | 0 | 0.998 | 0.880 | 0.938 | 0.823 | 0.999 |
| rps26l | rpl11 | 1501604 | 1488101 | ENSDARP00000111782 | ENSDARP00000063869 | 0 | 0 | 0 | 0 | 0.987 | 0.292 | 0.900 | 0.370 | 0.999 |
| rps26l | rps10 | 1501604 | 1485542 | ENSDARP00000111782 | ENSDARP00000045900 | 0 | 0 | 0 | 0 | 0.952 | 0.839 | 0.900 | 0.507 | 0.999 |
| rps3 | eef2l2 | 1488779 | 1486009 | ENSDARP00000067802 | ENSDARP00000051080 | 0.493 | 0 | 0 | 0 | 0.949 | 0.837 | 0 | 0.360 | 0.996 |
| rps27.1 | rplp2 | 1484170 | 1483852 | ENSDARP00000029079 | ENSDARP00000025616 | 0 | 0 | 0 | 0 | 0.998 | 0.384 | 0 | 0.086 | 0.999 |
| rpl36a | ddost | 1490292 | 1486503 | ENSDARP00000075363 | ENSDARP00000054289 | 0 | 0 | 0 | 0 | 0 | 0 | 0.900 | 0 | 0.900 |
| gabarapa | atg4a | 1486066 | 1483184 | ENSDARP00000051546 | ENSDARP00000019179 | 0 | 0 | 0 | 0 | 0.084 | 0.640 | 0 | 0.768 | 0.917 |
| atp5h | atp5d | 1484892 | 1483512 | ENSDARP00000038799 | ENSDARP00000022528 | 0 | 0 | 0 | 0 | 0.942 | 0.879 | 0.932 | 0.585 | 0.999 |
| rps26 | rps23 | 1497897 | 1484618 | ENSDARP00000105328 | ENSDARP00000035273 | 0 | 0 | 0 | 0 | 0.988 | 0.861 | 0.900 | 0.673 | 0.999 |
| rbm8a | ybx1 | 1483952 | 1482235 | ENSDARP00000026575 | ENSDARP00000010482 | 0 | 0 | 0 | 0 | 0 | 0 | 0.900 | 0.174 | 0.913 |
| myl2 | tnnt3b | 1503455 | 1497962 | ENSDARP00000116241 | ENSDARP00000105443 | 0 | 0 | 0 | 0 | 0.167 | 0.176 | 0.900 | 0.185 | 0.936 |
| hsp90aa1.2 | zgc:86598 | 1483899 | 1483766 | ENSDARP00000026065 | ENSDARP00000024748 | 0 | 0 | 0 | 0 | 0.091 | 0.872 | 0 | 0.727 | 0.965 |
| psma3 | psmb3 | 1499751 | 1482604 | ENSDARP00000108560 | ENSDARP00000013624 | 0 | 0 | 0 | 0 | 0.748 | 0.974 | 0.964 | 0.464 | 0.999 |
| rps3 | rpl35 | 1488779 | 1483123 | ENSDARP00000067802 | ENSDARP00000018594 | 0.493 | 0 | 0 | 0 | 0.999 | 0 | 0.900 | 0.330 | 0.999 |
| rps26l | rpl18a | 1501604 | 1484877 | ENSDARP00000111782 | ENSDARP00000038658 | 0 | 0 | 0 | 0 | 0.998 | 0.363 | 0.900 | 0.120 | 0.999 |
| eef1a1a | rpl35 | 1497414 | 1483123 | ENSDARP00000104468 | ENSDARP00000018594 | 0 | 0 | 0 | 0 | 0.168 | 0 | 0.900 | 0 | 0.913 |
| psma2 | psmb3 | 1487227 | 1482604 | ENSDARP00000058679 | ENSDARP00000013624 | 0 | 0 | 0 | 0 | 0.994 | 0.978 | 0.964 | 0.606 | 0.999 |
| rps27.1 | eif4eb | 1484170 | 1481876 | ENSDARP00000029079 | ENSDARP00000007117 | 0 | 0 | 0 | 0 | 0.117 | 0 | 0.900 | 0 | 0.907 |
| pkmb | tpi1a | 1505987 | 1484521 | ENSDARP00000122764 | ENSDARP00000033907 | 0.069 | 0 | 0 | 0 | 0.818 | 0 | 0 | 0.610 | 0.928 |
| rpl7 | rpl35 | 1483164 | 1483123 | ENSDARP00000018980 | ENSDARP00000018594 | 0.493 | 0 | 0 | 0 | 0.999 | 0.978 | 0.900 | 0.749 | 0.999 |
| desmb | tnni2a.1 | 1488347 | 1484340 | ENSDARP00000065355 | ENSDARP00000031650 | 0 | 0 | 0 | 0 | 0 | 0 | 0.900 | 0.068 | 0.902 |
| rps10 | eif4eb | 1485542 | 1481876 | ENSDARP00000045900 | ENSDARP00000007117 | 0 | 0 | 0 | 0 | 0.165 | 0.072 | 0.900 | 0 | 0.915 |
| rbm8a | rpl3 | 1483952 | 1481524 | ENSDARP00000026575 | ENSDARP00000003700 | 0 | 0 | 0 | 0 | 0.092 | 0 | 0.900 | 0.402 | 0.941 |
| eno3 | gapdh | 1505202 | 1488083 | ENSDARP00000120742 | ENSDARP00000063799 | 0.493 | 0 | 0 | 0 | 0.874 | 0.475 | 0 | 0.612 | 0.985 |
| eno3 | gpib | 1505202 | 1482688 | ENSDARP00000120742 | ENSDARP00000014578 | 0.098 | 0 | 0 | 0 | 0.775 | 0.132 | 0.800 | 0.612 | 0.984 |
| eef1a1a | rps16 | 1497414 | 1488590 | ENSDARP00000104468 | ENSDARP00000066897 | 0 | 0 | 0 | 0 | 0.357 | 0.155 | 0.900 | 0 | 0.940 |
| eif2s1 | rpl35 | 1488921 | 1483123 | ENSDARP00000068470 | ENSDARP00000018594 | 0 | 0 | 0 | 0 | 0.132 | 0 | 0.900 | 0 | 0.909 |
| psma1 | psma2 | 1497161 | 1487227 | ENSDARP00000103895 | ENSDARP00000058679 | 0 | 0 | 0.268 | 0.841 | 0.985 | 0.978 | 0.964 | 0.733 | 0.999 |
| smyhc1 | myl1 | 1486922 | 1481643 | ENSDARP00000056852 | ENSDARP00000004932 | 0 | 0 | 0 | 0 | 0.697 | 0.203 | 0.900 | 0.231 | 0.979 |
| tcp1 | zgc:65894 | 1497474 | 1481340 | ENSDARP00000104575 | ENSDARP00000002175 | 0 | 0 | 0 | 0 | 0 | 0.278 | 0.900 | 0.132 | 0.931 |
| rps24 | rpl36a | 1493019 | 1490292 | ENSDARP00000091586 | ENSDARP00000075363 | 0 | 0 | 0 | 0 | 0.975 | 0.784 | 0.900 | 0.118 | 0.999 |
| rpsa | rps29 | 1506145 | 1487533 | ENSDARP00000123183 | ENSDARP00000060443 | 0.080 | 0 | 0 | 0 | 0.979 | 0.975 | 0.900 | 0.493 | 0.999 |
| eno3 | gapdhs | 1505202 | 1487169 | ENSDARP00000120742 | ENSDARP00000058383 | 0.493 | 0 | 0 | 0 | 0.702 | 0.475 | 0 | 0.612 | 0.965 |
| tpma | tnni2a.4 | 1484960 | 1484806 | ENSDARP00000039656 | ENSDARP00000037759 | 0 | 0 | 0 | 0 | 0.525 | 0.285 | 0 | 0.764 | 0.916 |
| NDUFC2 | ndufs4 | 1500789 | 1486007 | ENSDARP00000110392 | ENSDARP00000051054 | 0 | 0 | 0 | 0 | 0.767 | 0 | 0.933 | 0.228 | 0.987 |
| eif4a3 | rbm8a | 1484026 | 1483952 | ENSDARP00000027276 | ENSDARP00000026575 | 0 | 0 | 0 | 0 | 0.479 | 0.872 | 0.900 | 0.232 | 0.994 |
| tnni2a.1 | myl1 | 1484340 | 1481643 | ENSDARP00000031650 | ENSDARP00000004932 | 0 | 0 | 0 | 0 | 0.480 | 0.187 | 0.900 | 0.210 | 0.962 |
| rps26l | rpl36a | 1501604 | 1490292 | ENSDARP00000111782 | ENSDARP00000075363 | 0 | 0 | 0 | 0 | 0.999 | 0 | 0.900 | 0.136 | 0.999 |
| rpl36a | rpl28 | 1490292 | 1483702 | ENSDARP00000075363 | ENSDARP00000024189 | 0 | 0 | 0 | 0 | 0.999 | 0.916 | 0.900 | 0.712 | 0.999 |
| rps27.2 | rpl11 | 1489628 | 1488101 | ENSDARP00000072300 | ENSDARP00000063869 | 0 | 0 | 0 | 0 | 0.866 | 0.450 | 0.900 | 0.358 | 0.994 |
| rpl32 | eef2l2 | 1487450 | 1486009 | ENSDARP00000060004 | ENSDARP00000051080 | 0 | 0 | 0 | 0 | 0.735 | 0.633 | 0 | 0.247 | 0.920 |
| ndufs4 | atp5l | 1486007 | 1481947 | ENSDARP00000051054 | ENSDARP00000007716 | 0 | 0 | 0 | 0 | 0.918 | 0 | 0 | 0.067 | 0.922 |
| rps24 | eif4eb | 1493019 | 1481876 | ENSDARP00000091586 | ENSDARP00000007117 | 0 | 0 | 0 | 0 | 0.116 | 0 | 0.900 | 0 | 0.907 |
| rps26l | rpl19 | 1501604 | 1498085 | ENSDARP00000111782 | ENSDARP00000105649 | 0 | 0 | 0 | 0 | 0.993 | 0.309 | 0.900 | 0.365 | 0.999 |
| rpl22 | rpl11 | 1501438 | 1488101 | ENSDARP00000111487 | ENSDARP00000063869 | 0 | 0 | 0 | 0 | 0.974 | 0.976 | 0.900 | 0.698 | 0.999 |
| rps23 | eif4a3 | 1484618 | 1484026 | ENSDARP00000035273 | ENSDARP00000027276 | 0 | 0 | 0 | 0 | 0.182 | 0.073 | 0.900 | 0.085 | 0.923 |
| rpl22 | rpl35 | 1501438 | 1483123 | ENSDARP00000111487 | ENSDARP00000018594 | 0 | 0 | 0 | 0 | 0.979 | 0.958 | 0.900 | 0.148 | 0.999 |
| rpsa | rpl3 | 1506145 | 1481524 | ENSDARP00000123183 | ENSDARP00000003700 | 0.088 | 0 | 0.232 | 0 | 0.999 | 0.327 | 0.900 | 0.330 | 0.999 |
| ATP5B | atp5d | 1487508 | 1483512 | ENSDARP00000060309 | ENSDARP00000022528 | 0.493 | 0 | 0 | 0 | 0.941 | 0.940 | 0.938 | 0.734 | 0.999 |
| gapdh | aldoab | 1488083 | 1485195 | ENSDARP00000063799 | ENSDARP00000042199 | 0.257 | 0 | 0 | 0 | 0.427 | 0.206 | 0.949 | 0.473 | 0.990 |
| rpl11 | rpl18a | 1488101 | 1484877 | ENSDARP00000063869 | ENSDARP00000038658 | 0 | 0 | 0 | 0 | 0.989 | 0.972 | 0.900 | 0.096 | 0.999 |
| smyhc1 | tnni2a.4 | 1486922 | 1484806 | ENSDARP00000056852 | ENSDARP00000037759 | 0 | 0 | 0 | 0 | 0.117 | 0.188 | 0.900 | 0.802 | 0.984 |
| rpsa | rpl19 | 1506145 | 1498085 | ENSDARP00000123183 | ENSDARP00000105649 | 0 | 0 | 0 | 0 | 0.996 | 0.230 | 0.900 | 0.330 | 0.999 |
| rps23 | rpl3 | 1484618 | 1481524 | ENSDARP00000035273 | ENSDARP00000003700 | 0.493 | 0 | 0 | 0 | 0.964 | 0.476 | 0.900 | 0.330 | 0.999 |
| tnnc2 | MYL3 | 1493861 | 1488531 | ENSDARP00000095111 | ENSDARP00000066500 | 0 | 0 | 0 | 0.777 | 0 | 0 | 0.900 | 0 | 0.900 |
| ddost | rpl13 | 1486503 | 1485678 | ENSDARP00000054289 | ENSDARP00000047390 | 0 | 0 | 0 | 0 | 0.096 | 0 | 0.900 | 0 | 0.905 |
| rpl6 | rpl32 | 1493092 | 1487450 | ENSDARP00000091899 | ENSDARP00000060004 | 0.103 | 0 | 0 | 0 | 0.991 | 0.961 | 0.900 | 0.118 | 0.999 |
| smyhc1 | myl10 | 1486922 | 1485924 | ENSDARP00000056852 | ENSDARP00000050204 | 0 | 0 | 0 | 0 | 0.974 | 0.288 | 0 | 0.485 | 0.990 |
| tpma | mylz3 | 1484960 | 1483092 | ENSDARP00000039656 | ENSDARP00000018197 | 0 | 0 | 0 | 0 | 0.982 | 0.134 | 0 | 0.254 | 0.988 |
| eno2 | gpib | 1484405 | 1482688 | ENSDARP00000032456 | ENSDARP00000014578 | 0.098 | 0 | 0 | 0 | 0.742 | 0.132 | 0.800 | 0.612 | 0.981 |
| rpl18a | rplp2 | 1484877 | 1483852 | ENSDARP00000038658 | ENSDARP00000025616 | 0.099 | 0 | 0 | 0 | 0.998 | 0.797 | 0 | 0.064 | 0.999 |
| rpl13 | rplp2 | 1485678 | 1483852 | ENSDARP00000047390 | ENSDARP00000025616 | 0 | 0 | 0 | 0 | 0.999 | 0.797 | 0 | 0 | 0.999 |
| actc1a | pdlim7 | 1495696 | 1485440 | ENSDARP00000100434 | ENSDARP00000044908 | 0 | 0 | 0 | 0 | 0.905 | 0.071 | 0 | 0 | 0.907 |
| rps16 | rpl32 | 1488590 | 1487450 | ENSDARP00000066897 | ENSDARP00000060004 | 0 | 0 | 0 | 0 | 0.996 | 0.877 | 0.900 | 0.467 | 0.999 |
| spcs1 | rpl7 | 1490634 | 1483164 | ENSDARP00000076814 | ENSDARP00000018980 | 0 | 0 | 0 | 0 | 0.181 | 0 | 0.900 | 0 | 0.916 |
| eif2s1 | rps23 | 1488921 | 1484618 | ENSDARP00000068470 | ENSDARP00000035273 | 0 | 0 | 0 | 0 | 0.104 | 0 | 0.900 | 0.091 | 0.911 |
| rps10 | eif4a3 | 1485542 | 1484026 | ENSDARP00000045900 | ENSDARP00000027276 | 0 | 0 | 0 | 0 | 0.204 | 0 | 0.900 | 0 | 0.918 |
| rpl23 | rpl14 | 1489208 | 1486227 | ENSDARP00000069977 | ENSDARP00000052528 | 0.098 | 0 | 0 | 0 | 0.994 | 0.972 | 0.900 | 0.238 | 0.999 |
| aldoaa | eno1a | 1504686 | 1481528 | ENSDARP00000119413 | ENSDARP00000003738 | 0 | 0 | 0 | 0 | 0.865 | 0.070 | 0.800 | 0.626 | 0.989 |
| gapdh | ckmb | 1488083 | 1487353 | ENSDARP00000063799 | ENSDARP00000059365 | 0 | 0 | 0 | 0 | 0.884 | 0.210 | 0 | 0.160 | 0.918 |
| ckma | slc25a4 | 1484819 | 1484291 | ENSDARP00000037871 | ENSDARP00000030881 | 0 | 0 | 0 | 0 | 0.905 | 0.080 | 0.191 | 0.064 | 0.924 |
| tnnc2 | desmb | 1493861 | 1488347 | ENSDARP00000095111 | ENSDARP00000065355 | 0 | 0 | 0 | 0 | 0 | 0.071 | 0.900 | 0 | 0.903 |
| tnnt3b | slc25a4 | 1497962 | 1484291 | ENSDARP00000105443 | ENSDARP00000030881 | 0 | 0 | 0 | 0 | 0.981 | 0 | 0 | 0 | 0.981 |
| rps29 | rps23 | 1487533 | 1484618 | ENSDARP00000060443 | ENSDARP00000035273 | 0.493 | 0 | 0 | 0 | 0.987 | 0.978 | 0.900 | 0.596 | 0.999 |
| rpl36a | rpl11 | 1490292 | 1488101 | ENSDARP00000075363 | ENSDARP00000063869 | 0 | 0 | 0 | 0 | 0.997 | 0.965 | 0.900 | 0.405 | 0.999 |
| rps27.1 | rpl3 | 1484170 | 1481524 | ENSDARP00000029079 | ENSDARP00000003700 | 0 | 0 | 0 | 0 | 0.970 | 0 | 0.900 | 0.330 | 0.997 |
| atp5a1 | hsp90aa1.2 | 1484089 | 1483899 | ENSDARP00000027947 | ENSDARP00000026065 | 0 | 0 | 0 | 0 | 0.100 | 0.965 | 0 | 0.344 | 0.977 |
| eef1a1a | rps23 | 1497414 | 1484618 | ENSDARP00000104468 | ENSDARP00000035273 | 0.252 | 0 | 0 | 0 | 0.351 | 0.234 | 0.900 | 0 | 0.957 |
| pkmb | aldocb | 1505987 | 1483738 | ENSDARP00000122764 | ENSDARP00000024492 | 0.069 | 0 | 0 | 0 | 0.693 | 0 | 0.800 | 0.608 | 0.974 |
| gapdhs | gpib | 1487169 | 1482688 | ENSDARP00000058383 | ENSDARP00000014578 | 0.142 | 0 | 0 | 0 | 0.275 | 0 | 0.800 | 0.504 | 0.931 |
| rpl23 | rps23 | 1489208 | 1484618 | ENSDARP00000069977 | ENSDARP00000035273 | 0.493 | 0 | 0 | 0 | 0.998 | 0.676 | 0.900 | 0.330 | 0.999 |
| rpl35 | rpl3 | 1483123 | 1481524 | ENSDARP00000018594 | ENSDARP00000003700 | 0.493 | 0 | 0 | 0 | 0.990 | 0.965 | 0.900 | 0.841 | 0.999 |
| EIF3F | rps16 | 1495369 | 1488590 | ENSDARP00000099664 | ENSDARP00000066897 | 0 | 0 | 0 | 0 | 0.754 | 0 | 0.900 | 0 | 0.975 |
| nnt | IDH2 | 1484423 | 1484037 | ENSDARP00000032730 | ENSDARP00000027389 | 0 | 0 | 0 | 0 | 0.111 | 0 | 0.900 | 0.234 | 0.925 |
| rps23 | rps27.1 | 1484618 | 1484170 | ENSDARP00000035273 | ENSDARP00000029079 | 0 | 0 | 0 | 0 | 0.976 | 0.800 | 0.900 | 0.569 | 0.999 |
| eif2s1 | rps3a | 1488921 | 1486104 | ENSDARP00000068470 | ENSDARP00000051762 | 0 | 0 | 0 | 0 | 0.159 | 0 | 0.900 | 0 | 0.912 |
| myl10 | tpm3 | 1485924 | 1481594 | ENSDARP00000050204 | ENSDARP00000004352 | 0 | 0 | 0 | 0 | 0.960 | 0.122 | 0 | 0.152 | 0.968 |
| rps3a | rps27.1 | 1486104 | 1484170 | ENSDARP00000051762 | ENSDARP00000029079 | 0 | 0 | 0 | 0 | 0.999 | 0.801 | 0.900 | 0.364 | 0.999 |
| rps3a | eif4a3 | 1486104 | 1484026 | ENSDARP00000051762 | ENSDARP00000027276 | 0 | 0 | 0 | 0 | 0.257 | 0.198 | 0.900 | 0 | 0.936 |
| EIF3F | rps23 | 1495369 | 1484618 | ENSDARP00000099664 | ENSDARP00000035273 | 0 | 0 | 0 | 0 | 0.909 | 0 | 0.900 | 0 | 0.990 |
| ybx1 | u2af1 | 1482235 | 1481720 | ENSDARP00000010482 | ENSDARP00000005582 | 0 | 0 | 0 | 0 | 0 | 0 | 0.900 | 0.136 | 0.909 |
| NDUFC2 | atp5l | 1500789 | 1481947 | ENSDARP00000110392 | ENSDARP00000007716 | 0 | 0 | 0 | 0 | 0.965 | 0 | 0 | 0.178 | 0.971 |
| rpl19 | eif2s1 | 1498085 | 1488921 | ENSDARP00000105649 | ENSDARP00000068470 | 0 | 0 | 0 | 0 | 0.118 | 0 | 0.900 | 0.212 | 0.924 |
| rps24 | rpl5a | 1493019 | 1481770 | ENSDARP00000091586 | ENSDARP00000006085 | 0 | 0 | 0 | 0 | 0.781 | 0.633 | 0.900 | 0.741 | 0.997 |
| rps3a | rpl13 | 1486104 | 1485678 | ENSDARP00000051762 | ENSDARP00000047390 | 0 | 0 | 0 | 0 | 0.999 | 0.875 | 0.900 | 0.088 | 0.999 |
| NDUFC2 | atp5d | 1500789 | 1483512 | ENSDARP00000110392 | ENSDARP00000022528 | 0 | 0 | 0 | 0 | 0.979 | 0 | 0 | 0.142 | 0.981 |
| desma | tnnc1a | 1490431 | 1483843 | ENSDARP00000075994 | ENSDARP00000025541 | 0 | 0 | 0 | 0 | 0 | 0.071 | 0.900 | 0.138 | 0.912 |
| rps27.2 | rpl18a | 1489628 | 1484877 | ENSDARP00000072300 | ENSDARP00000038658 | 0 | 0 | 0 | 0 | 0.996 | 0 | 0.900 | 0.166 | 0.999 |
| rpsa | rpl18a | 1506145 | 1484877 | ENSDARP00000123183 | ENSDARP00000038658 | 0 | 0 | 0 | 0 | 0.999 | 0.288 | 0.900 | 0.151 | 0.999 |
| rps16 | rpl28 | 1488590 | 1483702 | ENSDARP00000066897 | ENSDARP00000024189 | 0 | 0 | 0 | 0 | 0.999 | 0 | 0.900 | 0.234 | 0.999 |
| eef1a1a | rpl6 | 1497414 | 1493092 | ENSDARP00000104468 | ENSDARP00000091899 | 0 | 0 | 0 | 0 | 0.493 | 0.163 | 0.900 | 0.071 | 0.955 |
| rpl22 | EIF3F | 1501438 | 1495369 | ENSDARP00000111487 | ENSDARP00000099664 | 0 | 0 | 0 | 0 | 0.774 | 0 | 0.900 | 0 | 0.977 |
| rps26 | rpl3 | 1497897 | 1481524 | ENSDARP00000105328 | ENSDARP00000003700 | 0 | 0 | 0 | 0 | 0.990 | 0.308 | 0.900 | 0.382 | 0.999 |
| rpsa | rpl35 | 1506145 | 1483123 | ENSDARP00000123183 | ENSDARP00000018594 | 0 | 0 | 0 | 0 | 0.992 | 0.639 | 0.900 | 0.330 | 0.999 |
| rpl28 | rpl35 | 1483702 | 1483123 | ENSDARP00000024189 | ENSDARP00000018594 | 0 | 0 | 0 | 0 | 0.996 | 0.957 | 0.900 | 0.375 | 0.999 |
| rpl13 | eif4a3 | 1485678 | 1484026 | ENSDARP00000047390 | ENSDARP00000027276 | 0 | 0 | 0 | 0 | 0.221 | 0.200 | 0.900 | 0 | 0.933 |
| rps29 | rps10 | 1487533 | 1485542 | ENSDARP00000060443 | ENSDARP00000045900 | 0 | 0 | 0 | 0 | 0.984 | 0.978 | 0.900 | 0.717 | 0.999 |
| rpsa | rps3 | 1506145 | 1488779 | ENSDARP00000123183 | ENSDARP00000067802 | 0.082 | 0 | 0.203 | 0 | 0.999 | 0.977 | 0.900 | 0.360 | 0.999 |
| tcp1 | pfdn2 | 1497474 | 1484617 | ENSDARP00000104575 | ENSDARP00000035261 | 0 | 0 | 0 | 0 | 0.118 | 0.128 | 0.900 | 0.218 | 0.931 |
| rps27.2 | rpl3 | 1489628 | 1481524 | ENSDARP00000072300 | ENSDARP00000003700 | 0 | 0 | 0 | 0 | 0.918 | 0 | 0.900 | 0.330 | 0.994 |
| rpl32 | rps3a | 1487450 | 1486104 | ENSDARP00000060004 | ENSDARP00000051762 | 0 | 0 | 0 | 0 | 0.995 | 0.877 | 0.900 | 0.340 | 0.999 |
| tcp1 | mob4 | 1497474 | 1489802 | ENSDARP00000104575 | ENSDARP00000072975 | 0 | 0 | 0 | 0 | 0 | 0.978 | 0 | 0 | 0.978 |
| tnni2a.1 | mylz3 | 1484340 | 1483092 | ENSDARP00000031650 | ENSDARP00000018197 | 0 | 0 | 0 | 0 | 0.099 | 0.187 | 0.900 | 0.181 | 0.931 |
| rpl23 | eef2l2 | 1489208 | 1486009 | ENSDARP00000069977 | ENSDARP00000051080 | 0.493 | 0 | 0 | 0 | 0.797 | 0.663 | 0 | 0.330 | 0.973 |
| uqcrc1 | ndufv1 | 1499876 | 1486296 | ENSDARP00000108798 | ENSDARP00000052929 | 0 | 0 | 0 | 0 | 0.745 | 0 | 0.372 | 0.523 | 0.920 |
| desmb | mylz3 | 1488347 | 1483092 | ENSDARP00000065355 | ENSDARP00000018197 | 0 | 0 | 0 | 0 | 0 | 0.113 | 0.900 | 0 | 0.907 |
| rps26 | rpl11 | 1497897 | 1488101 | ENSDARP00000105328 | ENSDARP00000063869 | 0 | 0 | 0 | 0 | 0.992 | 0.292 | 0.900 | 0.626 | 0.999 |
| rpsa | rpl23 | 1506145 | 1489208 | ENSDARP00000123183 | ENSDARP00000069977 | 0.081 | 0 | 0 | 0 | 0.999 | 0.484 | 0.900 | 0.330 | 0.999 |
| rpl22 | rpl19 | 1501438 | 1498085 | ENSDARP00000111487 | ENSDARP00000105649 | 0 | 0 | 0 | 0 | 0.768 | 0.975 | 0.900 | 0.392 | 0.999 |
| rpl6 | ddost | 1493092 | 1486503 | ENSDARP00000091899 | ENSDARP00000054289 | 0 | 0 | 0 | 0 | 0.097 | 0 | 0.900 | 0 | 0.905 |
| pkmb | aldoaa | 1505987 | 1504686 | ENSDARP00000122764 | ENSDARP00000119413 | 0.069 | 0 | 0 | 0 | 0.693 | 0 | 0.800 | 0.608 | 0.974 |
| rpl6 | rpl35 | 1493092 | 1483123 | ENSDARP00000091899 | ENSDARP00000018594 | 0.075 | 0 | 0 | 0 | 0.950 | 0.975 | 0.900 | 0.150 | 0.999 |
| cox7c | atp5l | 1489195 | 1481947 | ENSDARP00000069896 | ENSDARP00000007716 | 0 | 0 | 0 | 0 | 0.931 | 0 | 0 | 0.771 | 0.984 |
| aldoaa | pgm1 | 1504686 | 1481818 | ENSDARP00000119413 | ENSDARP00000006510 | 0 | 0 | 0 | 0 | 0.543 | 0 | 0.800 | 0.270 | 0.930 |
| desmb | tpm3 | 1488347 | 1481594 | ENSDARP00000065355 | ENSDARP00000004352 | 0 | 0 | 0 | 0 | 0 | 0 | 0.900 | 0 | 0.900 |
| rpl36a | rpl23 | 1490292 | 1489208 | ENSDARP00000075363 | ENSDARP00000069977 | 0 | 0 | 0 | 0 | 0.999 | 0.965 | 0.900 | 0.071 | 0.999 |
| rps27.1 | rpl35 | 1484170 | 1483123 | ENSDARP00000029079 | ENSDARP00000018594 | 0 | 0 | 0 | 0 | 0.998 | 0.079 | 0.900 | 0.064 | 0.999 |
| hsp90aa1.2 | zgc:65894 | 1483899 | 1481340 | ENSDARP00000026065 | ENSDARP00000002175 | 0 | 0 | 0 | 0 | 0.069 | 0.073 | 0.900 | 0.489 | 0.949 |
| rps16 | rps10 | 1488590 | 1485542 | ENSDARP00000066897 | ENSDARP00000045900 | 0 | 0 | 0 | 0 | 0.984 | 0.978 | 0.900 | 0.507 | 0.999 |
| rpl11 | rpl28 | 1488101 | 1483702 | ENSDARP00000063869 | ENSDARP00000024189 | 0 | 0 | 0 | 0 | 0.978 | 0.957 | 0.900 | 0.151 | 0.999 |
| tnnt1 | tnni2a.1 | 1485382 | 1484340 | ENSDARP00000044153 | ENSDARP00000031650 | 0 | 0 | 0 | 0 | 0.993 | 0.454 | 0 | 0.765 | 0.999 |
| rps3 | rpl5a | 1488779 | 1481770 | ENSDARP00000067802 | ENSDARP00000006085 | 0.493 | 0 | 0 | 0 | 0.999 | 0.757 | 0.900 | 0.684 | 0.999 |
| rpl19 | eef2l2 | 1498085 | 1486009 | ENSDARP00000105649 | ENSDARP00000051080 | 0 | 0 | 0 | 0 | 0.743 | 0.662 | 0 | 0.247 | 0.928 |
| desmb | myl1 | 1488347 | 1481643 | ENSDARP00000065355 | ENSDARP00000004932 | 0 | 0 | 0 | 0 | 0 | 0.113 | 0.900 | 0 | 0.907 |
| rpl19 | spcs1 | 1498085 | 1490634 | ENSDARP00000105649 | ENSDARP00000076814 | 0 | 0 | 0 | 0 | 0.196 | 0 | 0.900 | 0 | 0.918 |
| EIF3F | rps29 | 1495369 | 1487533 | ENSDARP00000099664 | ENSDARP00000060443 | 0 | 0 | 0 | 0 | 0.818 | 0 | 0.900 | 0 | 0.981 |
| ddost | rps3a | 1486503 | 1486104 | ENSDARP00000054289 | ENSDARP00000051762 | 0 | 0 | 0 | 0 | 0.097 | 0 | 0.900 | 0 | 0.905 |
| rpl32 | rpl13 | 1487450 | 1485678 | ENSDARP00000060004 | ENSDARP00000047390 | 0 | 0 | 0 | 0 | 0.987 | 0.962 | 0.900 | 0.165 | 0.999 |
| rpl19 | ddost | 1498085 | 1486503 | ENSDARP00000105649 | ENSDARP00000054289 | 0 | 0 | 0 | 0 | 0.071 | 0 | 0.900 | 0 | 0.903 |
| actn2 | myl1 | 1494007 | 1481643 | ENSDARP00000095652 | ENSDARP00000004932 | 0 | 0 | 0 | 0 | 0.067 | 0.070 | 0.900 | 0.133 | 0.916 |
| gapdh | eno2 | 1488083 | 1484405 | ENSDARP00000063799 | ENSDARP00000032456 | 0.493 | 0 | 0 | 0 | 0.702 | 0.475 | 0 | 0.603 | 0.964 |
| rpl6 | rpl28 | 1493092 | 1483702 | ENSDARP00000091899 | ENSDARP00000024189 | 0 | 0 | 0 | 0 | 0.968 | 0.954 | 0.900 | 0.800 | 0.999 |
| rpl19 | rps3 | 1498085 | 1488779 | ENSDARP00000105649 | ENSDARP00000067802 | 0.493 | 0 | 0 | 0 | 0.999 | 0.874 | 0.900 | 0.418 | 0.999 |
| LOC567740 | tnnc2 | 1505894 | 1493861 | ENSDARP00000122502 | ENSDARP00000095111 | 0 | 0 | 0 | 0 | 0.099 | 0.200 | 0.900 | 0.491 | 0.958 |
| tnnc2 | smyhc1 | 1493861 | 1486922 | ENSDARP00000095111 | ENSDARP00000056852 | 0 | 0 | 0 | 0 | 0.888 | 0.200 | 0.900 | 0.721 | 0.997 |
| ldha | eno1a | 1487429 | 1481528 | ENSDARP00000059885 | ENSDARP00000003738 | 0.088 | 0 | 0 | 0 | 0.755 | 0 | 0 | 0.615 | 0.910 |
| rps27.2 | rpl23 | 1489628 | 1489208 | ENSDARP00000072300 | ENSDARP00000069977 | 0 | 0 | 0 | 0 | 0.987 | 0.450 | 0.900 | 0.330 | 0.999 |
| rps27.2 | rpl5a | 1489628 | 1481770 | ENSDARP00000072300 | ENSDARP00000006085 | 0 | 0 | 0 | 0 | 0.960 | 0.345 | 0.900 | 0.359 | 0.998 |
| pgm1 | eno1a | 1481818 | 1481528 | ENSDARP00000006510 | ENSDARP00000003738 | 0 | 0 | 0 | 0 | 0.931 | 0 | 0 | 0.352 | 0.955 |
| tnnc2 | pvalb1 | 1493861 | 1486619 | ENSDARP00000095111 | ENSDARP00000055061 | 0 | 0 | 0 | 0 | 0.899 | 0 | 0 | 0.471 | 0.944 |
| MYL3 | mylz3 | 1488531 | 1483092 | ENSDARP00000066500 | ENSDARP00000018197 | 0 | 0 | 0 | 0.965 | 0 | 0 | 0.900 | 0 | 0.900 |
| rpl6 | rps3a | 1493092 | 1486104 | ENSDARP00000091899 | ENSDARP00000051762 | 0 | 0 | 0 | 0 | 0.987 | 0.796 | 0.900 | 0.561 | 0.999 |
| rpl19 | eef1a1a | 1498085 | 1497414 | ENSDARP00000105649 | ENSDARP00000104468 | 0 | 0 | 0 | 0 | 0.421 | 0 | 0.900 | 0 | 0.939 |
| rps24 | rps10 | 1493019 | 1485542 | ENSDARP00000091586 | ENSDARP00000045900 | 0 | 0 | 0 | 0 | 0.764 | 0.977 | 0.900 | 0.693 | 0.999 |
| rpl6 | rps23 | 1493092 | 1484618 | ENSDARP00000091899 | ENSDARP00000035273 | 0 | 0 | 0 | 0 | 0.963 | 0.874 | 0.900 | 0.150 | 0.999 |
| rpl23 | rps3 | 1489208 | 1488779 | ENSDARP00000069977 | ENSDARP00000067802 | 0.493 | 0 | 0 | 0 | 0.999 | 0.708 | 0.900 | 0.358 | 0.999 |
| rps26 | rpl36a | 1497897 | 1490292 | ENSDARP00000105328 | ENSDARP00000075363 | 0 | 0 | 0 | 0 | 0.999 | 0 | 0.900 | 0.136 | 0.999 |
| cox5b2 | cox4i2 | 1497829 | 1493898 | ENSDARP00000105206 | ENSDARP00000095260 | 0 | 0 | 0 | 0 | 0.711 | 0.446 | 0.345 | 0.498 | 0.941 |
| tnnt3b | tnni2a.1 | 1497962 | 1484340 | ENSDARP00000105443 | ENSDARP00000031650 | 0 | 0 | 0 | 0 | 0.993 | 0.635 | 0.900 | 0.772 | 0.999 |
| actn2 | mylz3 | 1494007 | 1483092 | ENSDARP00000095652 | ENSDARP00000018197 | 0 | 0 | 0 | 0 | 0 | 0.070 | 0.900 | 0.091 | 0.908 |
| rps26l | rpl5a | 1501604 | 1481770 | ENSDARP00000111782 | ENSDARP00000006085 | 0 | 0 | 0 | 0 | 0.991 | 0.141 | 0.900 | 0.611 | 0.999 |
| rpl11 | rpl32 | 1488101 | 1487450 | ENSDARP00000063869 | ENSDARP00000060004 | 0.493 | 0 | 0 | 0 | 0.958 | 0.965 | 0.900 | 0.150 | 0.999 |
| rpl18a | rpl7 | 1484877 | 1483164 | ENSDARP00000038658 | ENSDARP00000018980 | 0 | 0 | 0 | 0 | 0.983 | 0.973 | 0.900 | 0.162 | 0.999 |
| rpl14 | rps10 | 1486227 | 1485542 | ENSDARP00000052528 | ENSDARP00000045900 | 0 | 0 | 0 | 0 | 0.958 | 0.629 | 0.900 | 0.184 | 0.998 |
| myl2 | tpm3 | 1503455 | 1481594 | ENSDARP00000116241 | ENSDARP00000004352 | 0 | 0 | 0 | 0 | 0.103 | 0.122 | 0.900 | 0.152 | 0.924 |
| rpl23 | rplp2 | 1489208 | 1483852 | ENSDARP00000069977 | ENSDARP00000025616 | 0 | 0 | 0 | 0 | 0.999 | 0.875 | 0 | 0.080 | 0.999 |
| eif3m | rpl5a | 1483698 | 1481770 | ENSDARP00000024129 | ENSDARP00000006085 | 0 | 0 | 0 | 0 | 0.920 | 0 | 0 | 0 | 0.922 |
| rps29 | eif4eb | 1487533 | 1481876 | ENSDARP00000060443 | ENSDARP00000007117 | 0 | 0 | 0 | 0 | 0.100 | 0 | 0.900 | 0.069 | 0.908 |
| rps3 | eif4eb | 1488779 | 1481876 | ENSDARP00000067802 | ENSDARP00000007117 | 0 | 0 | 0 | 0 | 0.278 | 0 | 0.900 | 0.119 | 0.930 |
| rpl36a | rpl32 | 1490292 | 1487450 | ENSDARP00000075363 | ENSDARP00000060004 | 0 | 0 | 0 | 0 | 0.996 | 0.964 | 0.900 | 0.375 | 0.999 |
| vcp | ubxn6 | 1482419 | 1482205 | ENSDARP00000012048 | ENSDARP00000010246 | 0 | 0 | 0 | 0 | 0 | 0.978 | 0.800 | 0.915 | 0.999 |
| rpl22 | rpl28 | 1501438 | 1483702 | ENSDARP00000111487 | ENSDARP00000024189 | 0 | 0 | 0 | 0 | 0.874 | 0.927 | 0.900 | 0.184 | 0.999 |
| rps23 | rpl28 | 1484618 | 1483702 | ENSDARP00000035273 | ENSDARP00000024189 | 0 | 0 | 0 | 0 | 0.998 | 0.121 | 0.900 | 0 | 0.999 |
| actc1a | actc1b | 1495696 | 1486630 | ENSDARP00000100434 | ENSDARP00000055135 | 0 | 0 | 0 | 0.990 | 0.960 | 0 | 0 | 0 | 0.960 |
| ampd3b | adss | 1485793 | 1484109 | ENSDARP00000048721 | ENSDARP00000028137 | 0.071 | 0 | 0 | 0 | 0 | 0 | 0.900 | 0.361 | 0.935 |
| rps24 | rpl14 | 1493019 | 1486227 | ENSDARP00000091586 | ENSDARP00000052528 | 0 | 0 | 0 | 0 | 0.878 | 0.825 | 0.900 | 0.216 | 0.998 |
| gpib | pgm1 | 1482688 | 1481818 | ENSDARP00000014578 | ENSDARP00000006510 | 0.246 | 0 | 0 | 0 | 0.770 | 0 | 0.939 | 0.617 | 0.995 |
| rpl22 | rpl18a | 1501438 | 1484877 | ENSDARP00000111487 | ENSDARP00000038658 | 0 | 0 | 0 | 0 | 0.667 | 0.974 | 0.900 | 0.244 | 0.999 |
| rpl36a | rpl35 | 1490292 | 1483123 | ENSDARP00000075363 | ENSDARP00000018594 | 0 | 0 | 0 | 0 | 0.999 | 0.965 | 0.900 | 0.507 | 0.999 |
| eif2s1 | rpl13 | 1488921 | 1485678 | ENSDARP00000068470 | ENSDARP00000047390 | 0 | 0 | 0 | 0 | 0.135 | 0.134 | 0.900 | 0 | 0.918 |
| rpl22 | rps24 | 1501438 | 1493019 | ENSDARP00000111487 | ENSDARP00000091586 | 0 | 0 | 0 | 0 | 0.953 | 0.700 | 0.900 | 0.548 | 0.999 |
| rpsa | rpl13 | 1506145 | 1485678 | ENSDARP00000123183 | ENSDARP00000047390 | 0 | 0 | 0 | 0 | 0.999 | 0.692 | 0.900 | 0.149 | 0.999 |
| rpl36a | rps29 | 1490292 | 1487533 | ENSDARP00000075363 | ENSDARP00000060443 | 0 | 0 | 0 | 0 | 0.999 | 0 | 0.900 | 0.502 | 0.999 |
| rps26l | rpl14 | 1501604 | 1486227 | ENSDARP00000111782 | ENSDARP00000052528 | 0 | 0 | 0 | 0 | 0.996 | 0.398 | 0.900 | 0.236 | 0.999 |
| rps29 | rpl35 | 1487533 | 1483123 | ENSDARP00000060443 | ENSDARP00000018594 | 0.493 | 0 | 0 | 0 | 0.999 | 0 | 0.900 | 0.728 | 0.999 |
| rps26 | rpl14 | 1497897 | 1486227 | ENSDARP00000105328 | ENSDARP00000052528 | 0 | 0 | 0 | 0 | 0.985 | 0.398 | 0.900 | 0.236 | 0.999 |
| rpl22 | rbm8a | 1501438 | 1483952 | ENSDARP00000111487 | ENSDARP00000026575 | 0 | 0 | 0 | 0 | 0.103 | 0 | 0.900 | 0 | 0.906 |
| rpl22 | rps23 | 1501438 | 1484618 | ENSDARP00000111487 | ENSDARP00000035273 | 0 | 0 | 0 | 0 | 0.722 | 0.662 | 0.900 | 0.141 | 0.991 |
| pomp | psmb3 | 1485465 | 1482604 | ENSDARP00000045123 | ENSDARP00000013624 | 0 | 0 | 0 | 0 | 0.722 | 0.940 | 0 | 0.118 | 0.984 |
| atp5c1 | ATP5B | 1488594 | 1487508 | ENSDARP00000066929 | ENSDARP00000060309 | 0.493 | 0 | 0.516 | 0 | 0.968 | 0.978 | 0.938 | 0.796 | 0.999 |
| btf3 | rpl36a | 1504780 | 1490292 | ENSDARP00000119683 | ENSDARP00000075363 | 0 | 0 | 0 | 0 | 0.967 | 0 | 0 | 0 | 0.968 |
| rps29 | rpl3 | 1487533 | 1481524 | ENSDARP00000060443 | ENSDARP00000003700 | 0.493 | 0 | 0 | 0 | 0.979 | 0 | 0.900 | 0.647 | 0.999 |
| rps26 | rpl32 | 1497897 | 1487450 | ENSDARP00000105328 | ENSDARP00000060004 | 0 | 0 | 0 | 0 | 0.970 | 0.435 | 0.900 | 0.118 | 0.998 |
| rps29 | rpl32 | 1487533 | 1487450 | ENSDARP00000060443 | ENSDARP00000060004 | 0.493 | 0 | 0 | 0 | 0.992 | 0 | 0.900 | 0.236 | 0.999 |
| rps16 | eef2l2 | 1488590 | 1486009 | ENSDARP00000066897 | ENSDARP00000051080 | 0.090 | 0 | 0 | 0 | 0.735 | 0.861 | 0 | 0.330 | 0.974 |
| rpl13 | rpl35 | 1485678 | 1483123 | ENSDARP00000047390 | ENSDARP00000018594 | 0 | 0 | 0 | 0 | 0.997 | 0.962 | 0.900 | 0.842 | 0.999 |
| eif4eb | rpl3 | 1481876 | 1481524 | ENSDARP00000007117 | ENSDARP00000003700 | 0 | 0 | 0 | 0 | 0.165 | 0 | 0.900 | 0.065 | 0.915 |
| uqcrc1 | ATP5B | 1499876 | 1487508 | ENSDARP00000108798 | ENSDARP00000060309 | 0 | 0 | 0 | 0 | 0.720 | 0.265 | 0 | 0.571 | 0.906 |
| rpl11 | rpl35 | 1488101 | 1483123 | ENSDARP00000063869 | ENSDARP00000018594 | 0.493 | 0 | 0 | 0 | 0.998 | 0.978 | 0.900 | 0.330 | 0.999 |
| rps24 | rps29 | 1493019 | 1487533 | ENSDARP00000091586 | ENSDARP00000060443 | 0 | 0 | 0 | 0 | 0.846 | 0.966 | 0.900 | 0.397 | 0.999 |
| rps26l | eef1a1a | 1501604 | 1497414 | ENSDARP00000111782 | ENSDARP00000104468 | 0 | 0 | 0 | 0 | 0.305 | 0 | 0.900 | 0 | 0.927 |
| actn2 | tnni2a.1 | 1494007 | 1484340 | ENSDARP00000095652 | ENSDARP00000031650 | 0 | 0 | 0 | 0 | 0.098 | 0.080 | 0.900 | 0.118 | 0.917 |
| eef1a1a | rpl13 | 1497414 | 1485678 | ENSDARP00000104468 | ENSDARP00000047390 | 0 | 0 | 0 | 0 | 0.283 | 0 | 0.900 | 0 | 0.925 |
| rpsa | rps16 | 1506145 | 1488590 | ENSDARP00000123183 | ENSDARP00000066897 | 0.493 | 0 | 0 | 0 | 0.999 | 0.978 | 0.900 | 0.330 | 0.999 |
| rps26 | rpl28 | 1497897 | 1483702 | ENSDARP00000105328 | ENSDARP00000024189 | 0 | 0 | 0 | 0 | 0.999 | 0 | 0.900 | 0.188 | 0.999 |
| tmem38a | myl1 | 1484755 | 1481643 | ENSDARP00000037150 | ENSDARP00000004932 | 0 | 0 | 0 | 0 | 0.918 | 0 | 0 | 0 | 0.918 |
| uqcrc1 | atp5c1 | 1499876 | 1488594 | ENSDARP00000108798 | ENSDARP00000066929 | 0 | 0 | 0 | 0 | 0.876 | 0.179 | 0 | 0.175 | 0.910 |
| rps3 | eif4a3 | 1488779 | 1484026 | ENSDARP00000067802 | ENSDARP00000027276 | 0 | 0 | 0 | 0 | 0.392 | 0.192 | 0.900 | 0.180 | 0.955 |
| rpl32 | rpl3 | 1487450 | 1481524 | ENSDARP00000060004 | ENSDARP00000003700 | 0.493 | 0 | 0 | 0 | 0.982 | 0.966 | 0.900 | 0.330 | 0.999 |
| rpl23 | rbm8a | 1489208 | 1483952 | ENSDARP00000069977 | ENSDARP00000026575 | 0 | 0 | 0 | 0 | 0.141 | 0 | 0.900 | 0 | 0.912 |
| rpl6 | rpl23 | 1493092 | 1489208 | ENSDARP00000091899 | ENSDARP00000069977 | 0.098 | 0 | 0 | 0 | 0.954 | 0.974 | 0.900 | 0.201 | 0.999 |
| aldoab | pgm1 | 1485195 | 1481818 | ENSDARP00000042199 | ENSDARP00000006510 | 0 | 0 | 0 | 0 | 0.507 | 0 | 0.800 | 0.201 | 0.916 |
| rpl14 | rpl13 | 1486227 | 1485678 | ENSDARP00000052528 | ENSDARP00000047390 | 0 | 0 | 0 | 0 | 0.998 | 0.973 | 0.900 | 0.246 | 0.999 |
| pdlim7 | myl1 | 1485440 | 1481643 | ENSDARP00000044908 | ENSDARP00000004932 | 0 | 0 | 0 | 0 | 0.976 | 0.083 | 0 | 0.065 | 0.978 |
| rps27.2 | eif4a3 | 1489628 | 1484026 | ENSDARP00000072300 | ENSDARP00000027276 | 0 | 0 | 0 | 0 | 0.137 | 0 | 0.900 | 0.118 | 0.917 |
| rps23 | eif4eb | 1484618 | 1481876 | ENSDARP00000035273 | ENSDARP00000007117 | 0 | 0 | 0 | 0 | 0.105 | 0 | 0.900 | 0.227 | 0.924 |
| spcs1 | rpl14 | 1490634 | 1486227 | ENSDARP00000076814 | ENSDARP00000052528 | 0 | 0 | 0 | 0 | 0.206 | 0 | 0.900 | 0 | 0.919 |
| rps16 | rpl18a | 1488590 | 1484877 | ENSDARP00000066897 | ENSDARP00000038658 | 0 | 0 | 0 | 0 | 0.999 | 0.874 | 0.900 | 0.207 | 0.999 |
| pvalb1 | slc25a4 | 1486619 | 1484291 | ENSDARP00000055061 | ENSDARP00000030881 | 0 | 0 | 0 | 0 | 0.935 | 0 | 0 | 0 | 0.935 |
| rpsa | eef1a1l1 | 1506145 | 1501580 | ENSDARP00000123183 | ENSDARP00000111742 | 0 | 0 | 0 | 0 | 0.969 | 0.157 | 0 | 0.067 | 0.974 |
| eef1a1a | rpl5a | 1497414 | 1481770 | ENSDARP00000104468 | ENSDARP00000006085 | 0 | 0 | 0 | 0 | 0.702 | 0.144 | 0.900 | 0.088 | 0.973 |
| ckmb | myl1 | 1487353 | 1481643 | ENSDARP00000059365 | ENSDARP00000004932 | 0 | 0 | 0 | 0 | 0.983 | 0.071 | 0 | 0.112 | 0.985 |
| ATP5B | hsp90aa1.2 | 1487508 | 1483899 | ENSDARP00000060309 | ENSDARP00000026065 | 0 | 0 | 0 | 0 | 0.157 | 0.880 | 0 | 0.163 | 0.907 |
| myl2 | mylz3 | 1503455 | 1483092 | ENSDARP00000116241 | ENSDARP00000018197 | 0 | 0 | 0 | 0 | 0.116 | 0.072 | 0.900 | 0.159 | 0.921 |
| eef1a1a | rps27.2 | 1497414 | 1489628 | ENSDARP00000104468 | ENSDARP00000072300 | 0 | 0 | 0 | 0 | 0.249 | 0 | 0.900 | 0 | 0.921 |
| rpl6 | eef2l2 | 1493092 | 1486009 | ENSDARP00000091899 | ENSDARP00000051080 | 0 | 0 | 0 | 0 | 0.717 | 0.664 | 0 | 0 | 0.903 |
| tnnc1a | myl1 | 1483843 | 1481643 | ENSDARP00000025541 | ENSDARP00000004932 | 0 | 0 | 0 | 0.782 | 0.118 | 0 | 0.900 | 0.088 | 0.909 |
| rps3 | rpl32 | 1488779 | 1487450 | ENSDARP00000067802 | ENSDARP00000060004 | 0.493 | 0 | 0 | 0 | 0.996 | 0.640 | 0.900 | 0.330 | 0.999 |
| EIF3F | rpl14 | 1495369 | 1486227 | ENSDARP00000099664 | ENSDARP00000052528 | 0 | 0 | 0 | 0 | 0.903 | 0 | 0.900 | 0 | 0.990 |
| rpl11 | rpl7 | 1488101 | 1483164 | ENSDARP00000063869 | ENSDARP00000018980 | 0.493 | 0 | 0 | 0 | 0.999 | 0.977 | 0.900 | 0.489 | 0.999 |
| actc1b | pdlim7 | 1486630 | 1485440 | ENSDARP00000055135 | ENSDARP00000044908 | 0 | 0 | 0 | 0 | 0.970 | 0.071 | 0 | 0 | 0.970 |
| rpsa | rpl28 | 1506145 | 1483702 | ENSDARP00000123183 | ENSDARP00000024189 | 0 | 0 | 0 | 0 | 0.997 | 0 | 0.900 | 0.146 | 0.999 |
| rpl22 | rpl5a | 1501438 | 1481770 | ENSDARP00000111487 | ENSDARP00000006085 | 0 | 0 | 0 | 0 | 0.990 | 0.875 | 0.900 | 0.662 | 0.999 |
| rps27.2 | rps23 | 1489628 | 1484618 | ENSDARP00000072300 | ENSDARP00000035273 | 0 | 0 | 0 | 0 | 0.972 | 0.800 | 0.900 | 0.569 | 0.999 |
| MDH2 | IDH2 | 1488058 | 1484037 | ENSDARP00000063661 | ENSDARP00000027389 | 0.257 | 0 | 0 | 0 | 0.793 | 0.411 | 0 | 0.913 | 0.991 |
| pkmb | eno1a | 1505987 | 1481528 | ENSDARP00000122764 | ENSDARP00000003738 | 0.257 | 0 | 0 | 0 | 0.994 | 0.128 | 0.957 | 0.608 | 0.999 |
