# Supplementary material for: Transcriptomic Characterization of Tambaqui (Colossoma macropomum, Cuvier, 1818) Exposed to Three Climate Change Scenarios
Source: PLoS One. 2016 Mar 28;11(3):e0152366. doi: 10.1371/journal.pone.0152366 (PMC4809510; doi:10.1371/journal.pone.0152366)
Supplement: S4 Table — (DOCX) [file pone.0152366.s009.docx]

Table S4: Genes of tambaqui after five and fifteen days of B1, A1B and A2 climate scenarios exposure grouped in five clusters using STRING software (v. 10).

| **STRING protein name** | **STRING id** | **Gene name and description** |  |
| --- | --- | --- | --- |
| **Cluster 1** | | |  |
| mob4 | 7955.ENSDARP00000072975 | MOB family member 4, phocein |  |
| tcp1 | 7955.ENSDARP00000104575 | t-complex polypeptide 1 |  |
| vbp1 | 7955.ENSDARP00000111810 | von Hippel-Lindau binding protein 1 |  |
| pfdn2 | 7955.ENSDARP00000035261 | prefoldin subunit 2 |  |
| zgc:65894 | 7955.ENSDARP00000002175 | zgc:65894; Tubulin is the major constituent of microtubules. It binds two moles of GTP, one at an exchangeable site on the beta chain and one at a non-exchangeable site on the alpha chain (By similarity) |  |
| hsp90aa1.1 | 7955.ENSDARP00000022302 | heat shock protein 90, alpha (cytosolic), class A member 1, tandem duplicate 1; Molecular chaperone that promotes the maturation, structural maintenance and proper regulation of specific target proteins involved for instance in cell cycle control and signal transduction. Undergoes a functional cycle that is linked to its ATPase activity. This cycle probably induces conformational changes in the client proteins, thereby causing their activation. Interacts dynamically with various co-chaperones that modulate its substrate recognition, ATPase cycle and chaperone function (By similarity). [...] |  |
| dnaja2 | 7955.ENSDARP00000028641 | DnaJ (Hsp40) homolog, subfamily A, member 2 |  |
| hsp90aa1.2 | 7955.ENSDARP00000026065 | heat shock protein 90, alpha (cytosolic), class A member 1, tandem duplicate 2 |  |
| zgc:86598 | 7955.ENSDARP00000024748 | zgc:86598 |  |
| **Cluster 2** | | |  |
| ATP5B | 7955.ENSDARP00000060309 | ATP synthase, H+ transporting, mitochondrial F1 complex, beta polypeptide; Produces ATP from ADP in the presence of a proton gradient across the membrane (By similarity) |  |
| atp5a1 | 7955.ENSDARP00000027947 | ATP synthase, H+ transporting, mitochondrial F1 complex, alpha subunit 1, cardiac muscle; Produces ATP from ADP in the presence of a proton gradient across the membrane (By similarity) |  |
| atp5h | 7955.ENSDARP00000038799 | ATP synthase, H+ transporting, mitochondrial F0 complex, subunit d |  |
| atp5l | 7955.ENSDARP00000007716 | ATP synthase, H+ transporting, mitochondrial F0 complex, subunit g |  |
| atp5d | 7955.ENSDARP00000022528 | ATP synthase, H+ transporting, mitochondrial F1 complex, delta subunit |  |
| atp5c1 | 7955.ENSDARP00000066929 | ATP synthase, H+ transporting, mitochondrial F1 complex, gamma polypeptide 1; Mitochondrial membrane ATP synthase (F(1)F(0) ATP synthase or Complex V) produces ATP from ADP in the presence of a proton gradient across the membrane which is generated by electron transport complexes of the respiratory chain. F-type ATPases consist of two structural domains, F(1) - containing the extramembraneous catalytic core, and F(0) - containing the membrane proton channel, linked together by a central stalk and a peripheral stalk. During catalysis, ATP synthesis in the catalytic domain of F(1) is cou [...] |  |
| ndufs4 | 7955.ENSDARP00000051054 | NADH dehydrogenase (ubiquinone) Fe-S protein 4, (NADH-coenzyme Q reductase) |  |
| ndufv1 | 7955.ENSDARP00000052929 | NADH dehydrogenase (ubiquinone) flavoprotein 1 |  |
| ndufa12 | 7955.ENSDARP00000062277 | NADH dehydrogenase (ubiquinone) 1 alpha subcomplex, 12 |  |
| NDUFC2 | 7955.ENSDARP00000110392 | NADH dehydrogenase (ubiquinone) 1, subcomplex unknown, 2, 14.5kDa; Accessory subunit of the mitochondrial membrane respiratory chain NADH dehydrogenase (Complex I), that is believed not to be involved in catalysis. Complex I functions in the transfer of electrons from NADH to the respiratory chain. The immediate electron acceptor for the enzyme is believed to be ubiquinone (By similarity) |  |
| uqcrc1 | 7955.ENSDARP00000108798 | ubiquinol-cytochrome c reductase core protein I |  |
| cox7c | 7955.ENSDARP00000069896 | cytochrome c oxidase, subunit VIIc |  |
| cox4i2 | 7955.ENSDARP00000095260 | cytochrome c oxidase subunit IV isoform 2 |  |
| cox5b2 | 7955.ENSDARP00000105206 | cytochrome c oxidase subunit Vb 2 |  |
| **Cluster 3** | | |  |
| aldoaa | 7955.ENSDARP00000119413 | aldolase a, fructose-bisphosphate, a |  |
| aldocb | 7955.ENSDARP00000024492 | aldolase C, fructose-bisphosphate, b |  |
| pgm1 | 7955.ENSDARP00000006510 | phosphoglucomutase 1 |  |
| gapdhs | 7955.ENSDARP00000058383 | glyceraldehyde-3-phosphate dehydrogenase, spermatogenic; Glyceraldehyde-3-phosphate dehydrogenase is a key enzyme in glycolysis that catalyzes the first step of the pathway by converting D-glyceraldehyde 3-phosphate (G3P) into 3-phospho-D- glyceroyl phosphate (By similarity) |  |
| eno3 | 7955.ENSDARP00000120742 | enolase 3, (beta, muscle) |  |
| gpib | 7955.ENSDARP00000014578 | glucose phosphate isomerase b |  |
| Gapdh | 7955.ENSDARP00000063799 | glyceraldehyde-3-phosphate dehydrogenase; Has both glyceraldehyde-3-phosphate dehydrogenase and nitrosylase activities, thereby playing a role in glycolysis and nuclear functions, respectively. Glyceraldehyde-3-phosphate dehydrogenase is a key enzyme in glycolysis that catalyzes the first step of the pathway by converting D-glyceraldehyde 3- phosphate (G3P) into 3-phospho-D-glyceroyl phosphate. Modulates the organization and assembly of the cytoskeleton. Also participates in nuclear events including transcription, RNA transport, DNA replication and apoptosis. Nuclear functions are prob [...] |  |
| pgk1 | 7955.ENSDARP00000070807 | phosphoglycerate kinase 1 |  |
| eno1a | 7955.ENSDARP00000003738 | enolase 1a, (alpha) |  |
| ldha | 7955.ENSDARP00000059885 | lactate dehydrogenase A4 |  |
| eno2 | 7955.ENSDARP00000032456 | enolase 2 |  |
| tpi1a | 7955.ENSDARP00000033907 | triosephosphate isomerase 1a |  |
| pkmb | 7955.ENSDARP00000122764 | pyruvate kinase, muscle, b |  |
| aldoab | 7955.ENSDARP00000042199 | aldolase a, fructose-bisphosphate, b |  |
| **Cluster 4** | | |  |
| ns:zf-e68 | 7955.ENSDARP00000111326 | myosin, heavy polypeptide 1.3, skeletal muscle |  |
| myl10 | 7955.ENSDARP00000050204 | myosin, light chain 10, regulatory |  |
| actc1a | 7955.ENSDARP00000100434 | hm:zewp0073 |  |
| tpma | 7955.ENSDARP00000039656 | alpha-tropomyosin; Binds to actin filaments in muscle and non-muscle cells. Plays a central role, in association with the troponin complex, in the calcium dependent regulation of vertebrate striated muscle contraction. Smooth muscle contraction is regulated by interaction with caldesmon. In non-muscle cells is implicated in stabilizing cytoskeleton actin filaments |  |
| actc1b | 7955.ENSDARP00000055135 | actin, alpha, cardiac muscle 1b |  |
| ckma | 7955.ENSDARP00000037871 | creatine kinase, muscle a |  |
| ckmb | 7955.ENSDARP00000059365 | creatine kinase, muscle b |  |
| pdlim7 | 7955.ENSDARP00000044908 | PDZ and LIM domain 7 |  |
| tnnc2 | 7955.ENSDARP00000095111 | troponin C type 2 (fast) |  |
| myl1 | 7955.ENSDARP00000004932 | myosin, light chain 1, alkali; skeletal, fast |  |
| mylz3 | 7955.ENSDARP00000018197 | myosin, light polypeptide 3, skeletal muscle |  |
| smyhc1 | 7955.ENSDARP00000056852 | slow myosin heavy chain 1 |  |
| tpm3 | 7955.ENSDARP00000004352 | tropomyosin 3 |  |
| slc25a4 | 7955.ENSDARP00000030881 | solute carrier family 25 (mitochondrial carrier; adenine nucleotide translocator), member 4 |  |
| atp2a1l | 7955.ENSDARP00000096674 | ATPase, Ca++ transporting, cardiac muscle, fast twitch 1 like |  |
| desma | 7955.ENSDARP00000075994 | desmin a |  |
| tnnt3b | 7955.ENSDARP00000105443 | troponin T3b, skeletal, fast |  |
| mylpfb | 7955.ENSDARP00000023063 | myosin light chain, phosphorylatable, fast skeletal muscle b |  |
| rock2a | 7955.ENSDARP00000122621 | rho-associated, coiled-coil containing protein kinase 2a |  |
| myl2 | 7955.ENSDARP00000116241 | myosin, light polypeptide 2, regulatory, cardiac, slow |  |
| LOC567740 | 7955.ENSDARP00000122502 | Uncharacterized protein |  |
| tnni2a.4 | 7955.ENSDARP00000037759 | troponin I, skeletal, fast 2a.4 |  |
| MYL3 | 7955.ENSDARP00000066500 | myosin, light chain 3, alkali; ventricular, skeletal, slow |  |
| pvalb1 | 7955.ENSDARP00000055061 | parvalbumin 1 |  |
| tmem38a | 7955.ENSDARP00000037150 | transmembrane protein 38A; Monovalent cation channel required for maintenance of rapid intracellular calcium release. May act as a potassium counter-ion channel that functions in synchronization with calcium release from intracellular stores (By similarity) |  |
| tnnc1b | 7955.ENSDARP00000054663 | troponin C type 1b (slow) |  |
| actn2 | 7955.ENSDARP00000095652 | actinin, alpha 2 |  |
| desmb | 7955.ENSDARP00000065355 | desmin b |  |
| tnnc1a | 7955.ENSDARP00000025541 | troponin C type 1a (slow) |  |
| tnni2a.1 | 7955.ENSDARP00000031650 | troponin I, skeletal, fast 2a.1 |  |
| calm1a | 7955.ENSDARP00000092307 | calmodulin 1b; Calmodulin mediates the control of a large number of enzymes, ion channels and other proteins by Ca(2+). Among the enzymes to be stimulated by the calmodulin-Ca(2+) complex are a number of protein kinases and phosphatases |  |
| tnnt1 | 7955.ENSDARP00000044153 | troponin T2c, cardiac |  |
| **Cluster 5** | | |  |
| eif4ebp3l | 7955.ENSDARP00000060989 | eukaryotic translation initiation factor 4E binding protein 3, like; Regulates eif4e1a activity by preventing its assembly into the eIF4F complex (By similarity) |  |
| eif2s1 | 7955.ENSDARP00000068470 | eukaryotic translation initiation factor 2, subunit 1 alpha |  |
| EIF3F | 7955.ENSDARP00000099664 | eukaryotic translation initiation factor 3, subunit F; Component of the eukaryotic translation initiation factor 3 (eIF-3) complex, which is involved in protein synthesis and, together with other initiation factors, stimulates binding of mRNA and methionyl-tRNAi to the 40S ribosome (By similarity) |  |
| eef1a1a | 7955.ENSDARP00000104468 | eukaryotic translation elongation factor 1 alpha 1a; This protein promotes the GTP-dependent binding of aminoacyl-tRNA to the A-site of ribosomes during protein biosynthesis (By similarity) |  |
| rpl11 | 7955.ENSDARP00000063869 | ribosomal protein L11 |  |
| rpl5a | 7955.ENSDARP00000006085 | ribosomal protein L5a |  |
| rpl7 | 7955.ENSDARP00000018980 | ribosomal protein L7 |  |
| rps26 | 7955.ENSDARP00000105328 | ribosomal protein S26 |  |
| rps3a | 7955.ENSDARP00000051762 | ribosomal protein S3A |  |
| rpsa | 7955.ENSDARP00000123183 | ribosomal protein SA; Required for the assembly and/or stability of the 40S ribosomal subunit. Required for the processing of the 20S rRNA- precursor to mature 18S rRNA in a late step of the maturation of 40S ribosomal subunits. Also functions as a cell surface receptor for laminin. Plays a role in cell adhesion to the basement membrane and in the consequent activation of signaling transduction pathways. May play a role in cell fate determination and tissue morphogenesis (By similarity) |  |
| rps29 | 7955.ENSDARP00000060443 | ribosomal protein S29 |  |
| rps26l | 7955.ENSDARP00000111782 | ribosomal protein S26, like |  |
| eif4a3 | 7955.ENSDARP00000027276 | eukaryotic translation initiation factor 4A, isoform 3; ATP-dependent RNA helicase. Component of a splicing- dependent multiprotein exon junction complex (EJC) deposited at splice junction on mRNAs. The EJC is a dynamic structure consisting of a few core proteins and several more peripheral nuclear and cytoplasmic associated factors that join the complex only transiently either during EJC assembly or during subsequent mRNA metabolism. Core components of the EJC, that remains bound to spliced mRNAs throughout all stages of mRNA metabolism, functions to mark the position of the exon-exon [...] |  |
| rps3 | 7955.ENSDARP00000067802 | ribosomal protein S3 |  |
| eef2l2 | 7955.ENSDARP00000051080 | eukaryotic translation elongation factor 2, like 2 |  |
| rps27.1 | 7955.ENSDARP00000029079 | ribosomal protein S27, isoform 1 |  |
| rps23 | 7955.ENSDARP00000035273 | ribosomal protein S23 |  |
| rpl18a | 7955.ENSDARP00000038658 | ribosomal protein L18a |  |
| rps27.2 | 7955.ENSDARP00000072300 | ribosomal protein S27, isoform 2 |  |
| rpl35 | 7955.ENSDARP00000018594 | ribosomal protein L35; Plays an essential role in early embryonic development. May act as a haploinsufficient tumor supressor |  |
| rpl28 | 7955.ENSDARP00000024189 | ribosomal protein L28 |  |
| rpl22 | 7955.ENSDARP00000111487 | ribosomal protein L22 |  |
| eef1a1l1 | 7955.ENSDARP00000111742 | eukaryotic translation elongation factor 1 alpha 1, like 1; This protein promotes the GTP-dependent binding of aminoacyl-tRNA to the A-site of ribosomes during protein biosynthesis (By similarity) |  |
| rpl3 | 7955.ENSDARP00000003700 | ribosomal protein L3 |  |
| rpl32 | 7955.ENSDARP00000060004 | ribosomal protein L32 |  |
| ddost | 7955.ENSDARP00000054289 | dolichyl-diphosphooligosaccharide-protein glycosyltransferase; Essential subunit of the N-oligosaccharyl transferase (OST) complex which catalyzes the transfer of a high mannose oligosaccharide from a lipid-linked oligosaccharide donor to an asparagine residue within an Asn-X-Ser/Thr consensus motif in nascent polypeptide chains (By similarity) |  |
| rplp2 | 7955.ENSDARP00000025616 | ribosomal protein, large P2 |  |
| spcs1 | 7955.ENSDARP00000076814 | signal peptidase complex subunit 1 homolog (S. cerevisiae) |  |
| rbm8a | 7955.ENSDARP00000026575 | RNA binding motif protein 8A; Component of a splicing-dependent multiprotein exon junction complex (EJC) deposited at splice junction on mRNAs. The EJC is a dynamic structure consisting of a few core proteins and several more peripheral nuclear and cytoplasmic associated factors that join the complex only transiently either during EJC assembly or during subsequent mRNA metabolism. Core components of the EJC, that remains bound to spliced mRNAs throughout all stages of mRNA metabolism, functions to mark the position of the exon-exon junction in the mature mRNA and thereby influences dow [...] |  |
| rpl36a | 7955.ENSDARP00000075363 | ribosomal protein L36A |  |
| rpl14 | 7955.ENSDARP00000052528 | ribosomal protein L14 |  |
| rpl19 | 7955.ENSDARP00000105649 | ribosomal protein L19 |  |
| rps10 | 7955.ENSDARP00000045900 | ribosomal protein S10 |  |
| rps16 | 7955.ENSDARP00000066897 | ribosomal protein S16 |  |
| rpl6 | 7955.ENSDARP00000091899 | ribosomal protein L6 |  |
| rpl13 | 7955.ENSDARP00000047390 | ribosomal protein L13 |  |
| rpl23 | 7955.ENSDARP00000069977 | ribosomal protein L23 |  |
| rps24 | 7955.ENSDARP00000091586 | ribosomal protein S24 |  |
